# COMplot, A Graphical Presentation of Complication Profiles and Adverse Effects for the Curative Treatment of Gastric Cancer: A Systematic Review and Meta-Analysis

**DOI:** 10.3389/fonc.2019.00684

**Published:** 2019-07-25

**Authors:** Tom van den Ende, Frank A. Abe Nijenhuis, Héctor G. van den Boorn, Emil ter Veer, Maarten C. C. M. Hulshof, Suzanne S. Gisbertz, Martijn G. H. van Oijen, Hanneke W. M. van Laarhoven

**Affiliations:** ^1^Department of Medical Oncology, Cancer Center Amsterdam, Amsterdam University Medical Centers (UMC), University of Amsterdam, Amsterdam, Netherlands; ^2^Department of Radiotherapy, Cancer Center Amsterdam, Amsterdam University Medical Centers (UMC), University of Amsterdam, Amsterdam, Netherlands; ^3^Department of Surgery, Cancer Center Amsterdam, Amsterdam University Medical Centers (UMC), University of Amsterdam, Amsterdam, Netherlands

**Keywords:** gastric cancer, chemotherapy, curative, toxicity, meta-analysis

## Abstract

**Background:** For the curative treatment of gastric cancer, several neoadjuvant, and adjuvant treatment-regimens are available which have shown to improve overall survival. No overview is available regarding toxicity and surgery related outcomes. Our aim was to construct a novel graphical method concerning adverse events (AEs) associated with multimodality treatment and perform a meta-analysis to compare different clinically relevant cytotoxic regimens with each other.

**Methods:** The PubMed, EMBASE, CENTRAL, and ASCO/ESMO databases were searched up to May 2019 for randomized controlled trials investigating curative treatment regimens for gastric cancer. To construct single and bidirectional bar-charts (COMplots), grade 1–2 and grade 3–5 AEs were extracted per cytotoxic regimen. For surgery-related outcomes a pre-specified set of complications was used. Thereafter, treatment-arms comparing the same regimens were combined in a single-arm random-effects meta-analysis and pooled-proportions were calculated with 95% confidence-intervals. Comparative meta-analyses were performed based on clinical relevance and compound similarity.

**Results:** In total 16 RCTs (*n* = 4,526 patients) were included investigating pre-operative-therapy and 39 RCTs investigating adjuvant-therapy (*n* = 13,732 patients). Pre-operative COMplots were created for among others; 5-fluorouracil/leucovorin-oxaliplatin-docetaxel (FLOT), epirubicin-cisplatin-fluoropyrimidine (ECF), cisplatin-fluoropyrimidine (CF), and oxaliplatin-fluoropyrimidine (FOx). Pre-operative FLOT showed a minor increase in grade 1–2 and grade 3–4 AEs compared to pre-operative ECF, CF, and FOx. A pooled analysis of patients who had received pre-operative therapy compared to patients who underwent direct surgery did not reveal any significant difference in surgery related morbidity/mortality. When we compared three commonly used adjuvant regimens; S-1 had the lowest amount of grade 3–4 AEs compared to capecitabine with oxaliplatin (CAPOX) and 5-FU with radiotherapy (5-FU+RT).

**Conclusion:** COMplot provides a novel tool to visualize and compare treatment related AEs for gastric cancer. Based on our comparisons, pre-operative FLOT had a manageable toxicity profile compared to other pre-operative doublet or triplet regimens. We found no evidence indicating surgical outcomes might be hampered by pre-operative therapy. Adjuvant S-1 had a more favorable toxicity profile compared to CAPOX and 5-FU+RT.

## Introduction

Gastric cancer treated with curative intent has a poor prognosis with a 5-year survival varying between 30 and 40% (1–3). There are several different treatment strategies for gastric cancer which have showed overall survival benefit in the perioperative, neoadjuvant or adjuvant setting, for example, the perioperative FLOT regimen (5-fluorouracil, leucovorin, oxaliplatin, and docetaxel) and the MAGIC regimen (epirubicin, 5-fluorouracil, and cisplatin) (2, 3), adjuvant chemotherapy, i.e., S-1 alone or capecitabine with oxaliplatin (4, 5) and adjuvant chemoradiotherapy, i.e., 5-fluorouracil with radiotherapy (6). Clinical practice varies between geographical regions due to local preferences and possibly differences in tumor biology. Perioperative chemotherapy is the preferred strategy in Europe, adjuvant chemotherapy is preferred in Asia and in the United States adjuvant chemo(radio)therapy is given with or without neoadjuvant chemotherapy (7–9).

Treatment related adverse events (AEs) during multimodality treatment may encompass toxicity due to conventional cytotoxic therapy but also surgery related mortality/morbidity. Toxicity is usually scored according to the Common Terminology Criteria for Adverse Events (CTCAE), in which AEs are graded from mild (grade 1 or 2) to severe (grade 3 or 4) and fatal (grade 5) (10). Furthermore, the occurrence of AEs may not only affect the period during systemic treatment with chemo(radio)therapy, but may also influence post-operative complications. As physicians may offer several curative treatment options to patients with gastric cancer, systemic treatment related AEs will play an important role in shared decision making between patients and physicians.

Well-informed decisions concerning treatment can improve adherence and quality of life (11). Currently, no graphical overview is available of systemic treatment related AEs pooled from multiple studies in the curative setting for gastric cancer. Our aim was to construct a comprehensive graphical overview of multimodality related AEs for the curative treatment of gastric cancer in the neo(adjuvant) setting and compare different clinically relevant regimens with each other (COMplots). Therefore, we conducted a systematic review with meta-analysis.

## Methods

### Literature Search

PubMed, EMBASE, the Cochrane Central Register of Controlled Trials (CENTRAL), and the meeting abstracts from the American Society of Clinical Oncology (ASCO) and European Society for Medical Oncology (ESMO) were searched from 1977 up to May 2019. The search strategy consisted of medical subject headings (MeSH) and text words for gastric cancer and esophageal cancer (Supplementary Methods). Articles with esophageal adenocarcinoma patients where included if at least 20% of the total amount of patients had tumors located in the stomach. Two authors (TvdE, FaN) screened the titles, abstracts, and full articles independently. Article citations were cross-referenced to identify potentially missing articles. Discrepancies were discussed with a third arbiter (EtV or HvL) until consensus was reached.

### Study Selection and Quality Assessment

Prospective phase II or III randomized controlled trials (RCTs) on the curative treatment of gastric cancer were included. Studies were eligible if patients were treated with one of the following intravenous or oral cytotoxic agents: a fluoropyrimidine (5-fluorouracil, capecitabine, UFT, tegafur, or S-1), a platinum compound (either cisplatin or oxaliplatin), a taxane (either docetaxel or paclitaxel), an anthracycline (either epirubicin or doxorubicin), irinotecan, mitomycin C, or methotrexate. Treatment could be administered in the neoadjuvant, perioperative, or adjuvant setting. Studies which investigated chemoradiotherapy were also included. Only studies that reported data on grade 1–2 or grade 3–5 AEs and/or on surgical morbidity/mortality were included. Trials which included patients with metastatic disease at baseline were excluded. The quality of the studies was assessed using the Cochrane Risk of Bias tool (version 5.1.0). Items were scored as low, high, or unknown risk of bias.

### Data Extraction and Statistical Analysis

The incidence and severity of treatment related AEs, including the total number of patients who started treatment, were extracted from the study reports for each individual treatment arm. Moreover, surgery related complications were extracted. A pre-specified set of AEs was constructed based on the available data to enable cross study comparisons based on individual regimens.

All statistical analyses were performed with the metafor 2.0-0 package in R version 3.5.1. For each treatment arm, the incidence of AEs or surgical complications was analyzed through meta-analysis with a random-effects model after application of the logit transformation. This resulted in pooled proportions with 95% confidence intervals (95% CI). A graphical representation of the data for each treatment arm was visualized in a bar chart with the 95% CI (COMplot). For each treatment arm, individual bar charts were constructed using the number of patients, number of events and the number of trials. Clinically relevant regimens were selected based on international guidelines and individual RCTs showing significant overall survival benefit compared to surgery alone (3–9, 12–14). AEs of RCTs investigating pre-operative regimens (as part of a neoadjuvant or perioperative scheme) were pooled together if they investigated the same regimen. The AEs of post-operative therapy as part of a perioperative scheme were pooled separately from purely adjuvant RCTs due to the inclusion of different patients groups (e.g., amount of patients with R0 resection, prior exposure to cytotoxic therapy). Relevant pre-operative regimens were cisplatin or oxaliplatin with a fluoropyrimidine (CF or FOx), 5-FU, leucovorin, oxaliplatin, and docetaxel (FLOT), taxane, cisplatin, and a fluoropyrimidine (TCF) or epirubicin, cisplatin, and a fluoropyrimidine (ECF). Clinically relevant post-operative regimens as part of a perioperative scheme were CF, FOx, FLOT, TCF, ECF, and CF with radiotherapy (CF+RT). Relevant adjuvant regimens were a fluoropyrimidine singlet (F), a fluoroprimidine doublet with either cisplatin (CF), oxaliplatin (FOx), or a taxane (TF). Relevant chemoradiotherapy regimens were 5-FU (5-FU+RT) and cisplatin with 5-FU (CF+RT). Less relevant regimens were included in the (Supplementary Figures).

Surgery related morbidity was grouped according to the following categories: total morbidity, any medical complication, any reintervention, abscess, anastomotic leakage, bleeding, infection, intestinal occlusion, pulmonary complications, sepsis, and wound healing disorders. Surgery related mortality was defined as death up to 90 days after surgery, depending on data presented.

Differences in adverse events proportions between several clinically relevant regimens were tested with a Wald test. Additional two-sided *post-hoc* testing, with Holm correction for multiple comparison, was performed if the Wald test was significant (*p* < 0.05). Comparisons between regimens were represented with bidirectional COMplot charts.

## Results

**Figure 1:**
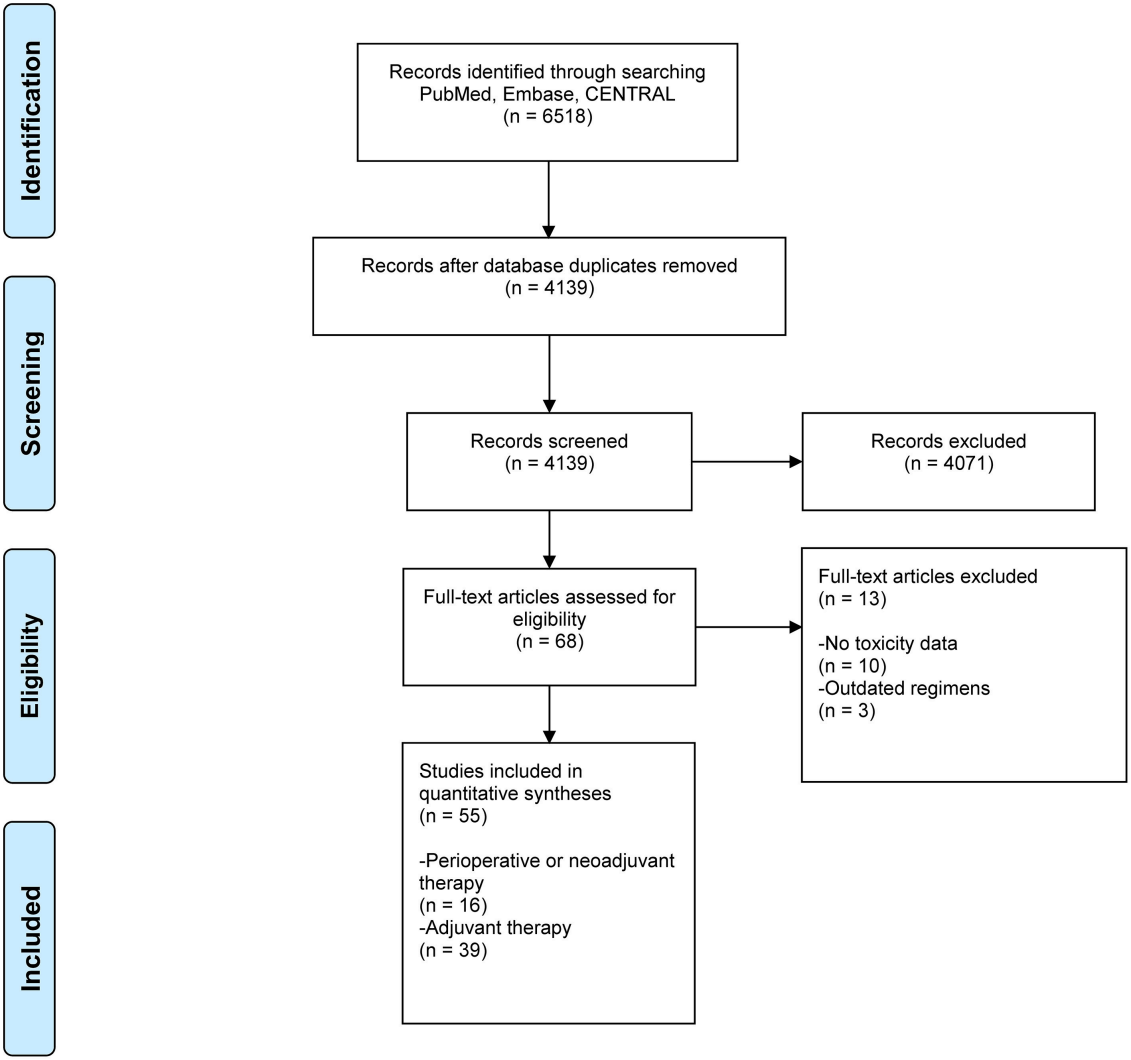
PRISMA flowchart of included studies. CENTRAL, Cochrane Central Register of Controlled Trials; PRISMA, Preferred Reporting Items for Systematic Reviews and Meta-Analyses.

In total 4,139 unique references were retrieved from the PubMed, Embase, and CENTRAL databases. Sixty-eight references were selected for full text assessment after title and abstract screening. From the ASCO and ESMO conference meeting abstracts no additional data was identified as the publications of large RCTs were available in full text (e.g., FLOT-4, CRITICS). Finally, 55 original RCTs could be included. Sixteen studies (2, 3, 12, 15–27) investigated perioperative or neoadjuvant therapy and 39 only adjuvant therapy (Figure 1) (4–6, 28–63). An overview of all included studies including dosage of study medication is presented in (Supplementary Table 1).

### Risk of Bias

The Cochrane Risk of bias tool was used to assess study quality (Supplementary Figure 1).

In 27 (49%) studies there was no risk of bias on any domain. Twelve (22%) studies were rated as unclear risk of bias on one domain and 10 (18%) studies on two domains. In six (11%) studies risk of bias was deemed unclear on three or more domains. There were no studies rated as having a high risk of bias.

### COMplot for Pre-operative and Post-operative Therapy

**Figure 2:**
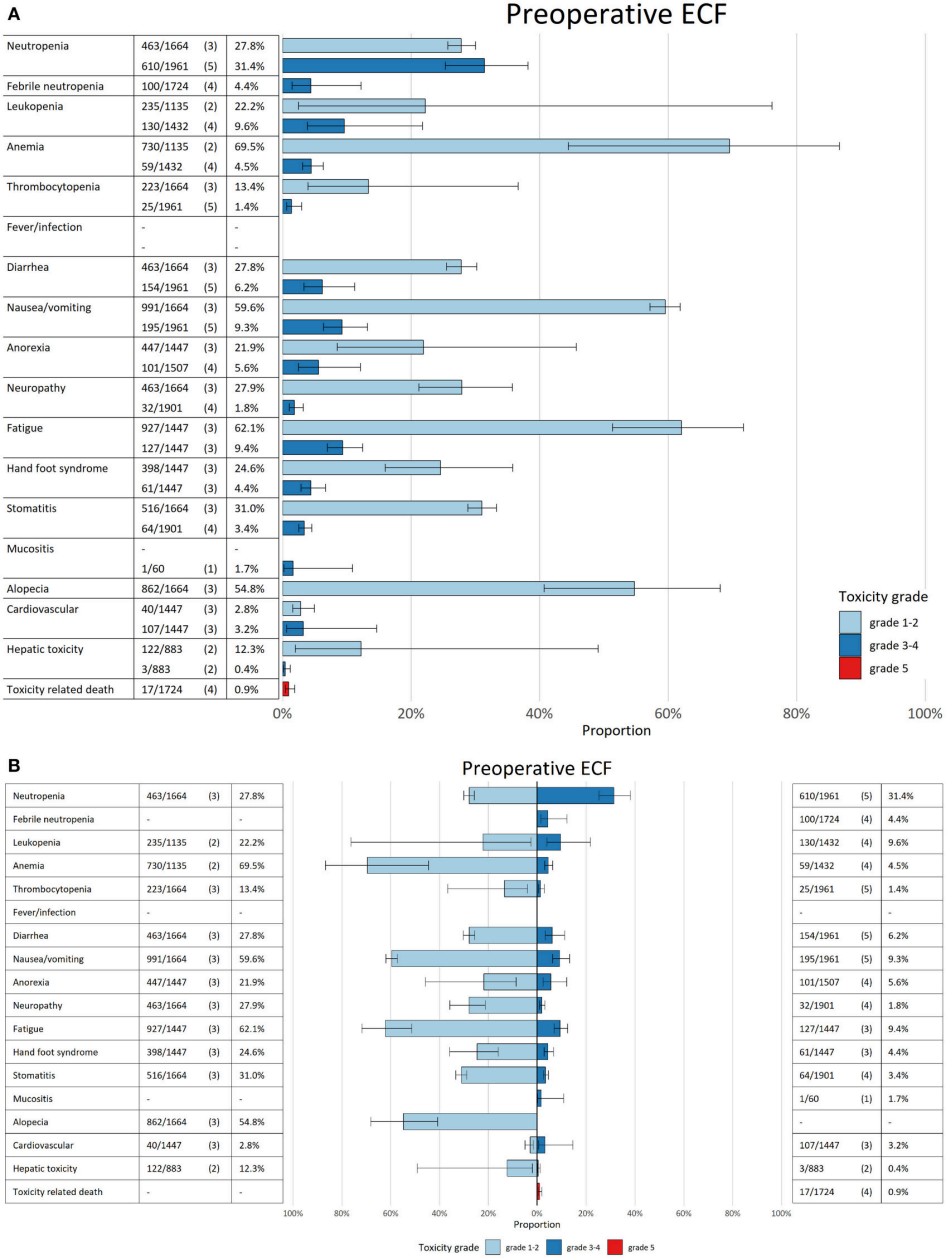
**(A)** Bar chart for pre-operative epirubicin, cisplatin, and a fluoropyrimidine (ECF). In the first column the adverse events are mentioned per row. In the second column the amount of patients with the AE per grade are mentioned compared to the total amount of patients who were treated with the regimen. In brackets the amount of studies are mentioned. The pooled estimated incidence for each AE is mentioned in the third column. The bars in the figure give the pooled estimate with 95% CI (line in black in the bar). Every bar has a specific color which corresponds with the grade of the AE (light blue grade 1–2, dark blue grade 3–4, and red grade 5). **(B)** Bidirectional bar chart for pre-operative epirubicin, cisplatin and a fluoropyrimidine (ECF). In the first column the adverse events are mentioned per row. In the second column the amount of patients with the AE per grade are mentioned compared to the total amount of patients who were treated with the regimen. In brackets the amount of studies are mentioned. The pooled estimated incidence for each AE is mentioned in the third column. The bars in the figure give the pooled estimate with 95% CI (line in black in the bar). Every bar has a specific color which corresponds with the grade of the AE (light blue grade 1–2, dark blue grade 3–4 and red grade 5).

**Figure 3:**
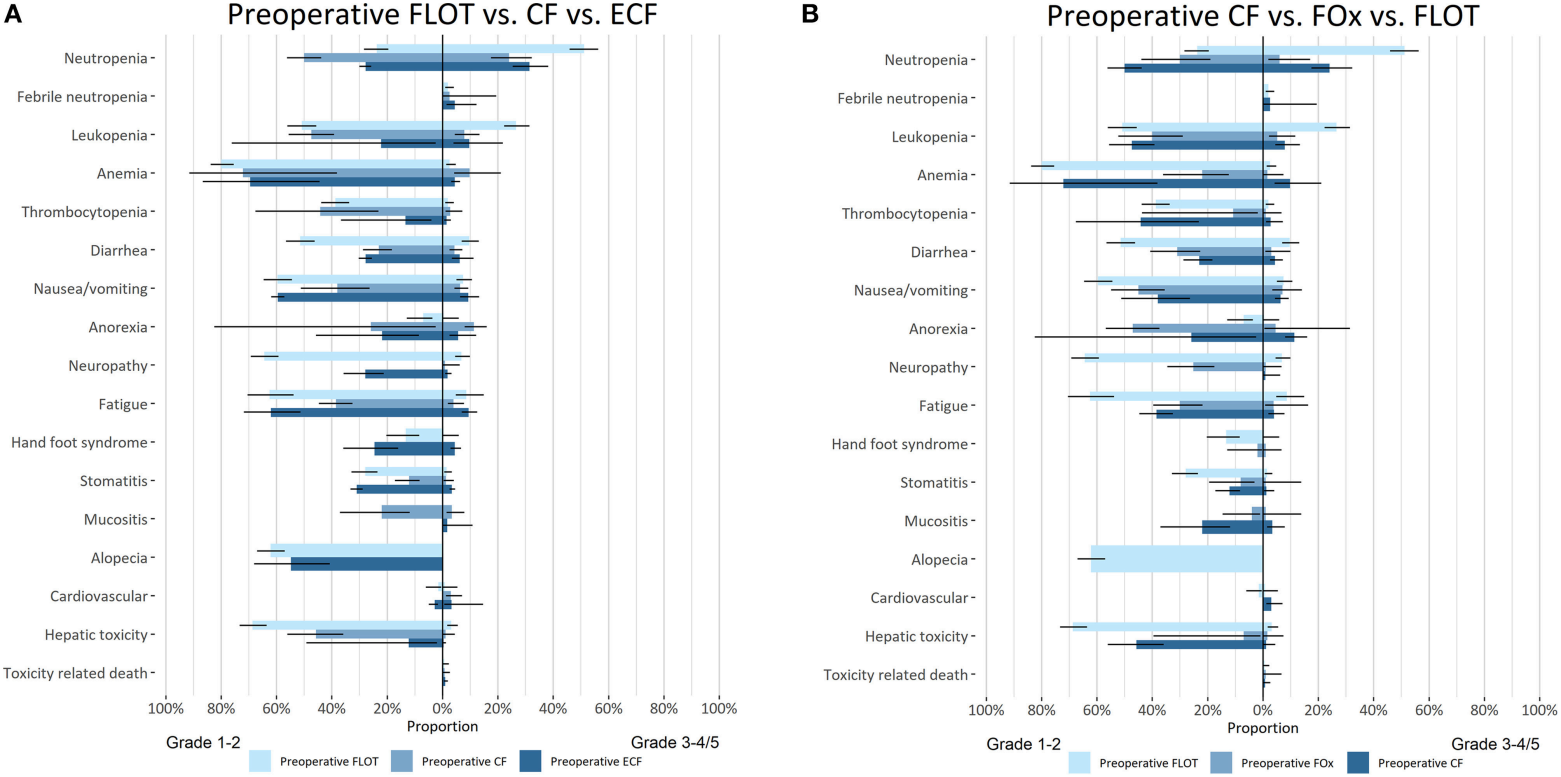
**(A)** Bidirectional comparative meta-analysis of pre-operative 5-fluorouracil/leucovorin-oxaliplatin-docetaxel (FLOT), epirubicin-cisplatin-fluoropyrimidine (ECF), and cisplatin-fluoropyrimidine (CF). In the column on the left of the figure the adverse events are mentioned per group of bar charts. The bars in the figure give the pooled estimate with 95% CI (line in black in the bar). Grade 1–2 AEs are depicted on the left of the figure and grade 3–4/5 on the right of the figure. The color of the bar chart indicates which regimen is depicted. **(B)** Bidirectional comparative meta-analysis of pre-operative 5-fluorouracil/leucovorin-oxaliplatin-docetaxel (FLOT), cisplatin-fluoropyrimidine (CF), and oxaliplatin-fluoropyrimidine (FOx). In the column on the left of the figure the adverse events are mentioned per group of bar charts. The bars in the figure give the pooled estimate with 95% CI (line in black in the bar). Grade 1–2 AEs are depicted on the left of the figure and grade 3–4/5 on the right of the figure. The color of the bar chart indicates which regimen is depicted.

For five clinically relevant pre-operative regimens, we constructed barcharts with confidence intervals and bidirectional charts with confidence intervals for adverse events (Figures 2A,B). The adverse events associated with systemic treatment, were subdivided for perioperative chemotherapy into different figures for pre-operative and post-operative therapy, if it was possible to identify this from individual RCTs. The AEs of trials investigating neoadjuvant therapy were pooled with the pre-operative arms of perioperative RCTs, if they investigated the same regimen. Comparisons were made between pre-operative FLOT, TCF, ECF, and two pre-operative fluoropyrmidine doublets; FOx and CF to identify any significant differences between grade 1–5 AEs (Figures 3A,B). In terms of grade 3–4 AEs, FLOT showed a minor increase in grade 3–4 AEs compared to ECF, CF, and FOx (mainly hematological toxicity: neutropenia and leukopenia). FLOT showed higher incidences of grade 1–2 AEs compared to CF and FOx (mainly gastrointestinal toxicity, stomatitis, and fatigue). FLOT also showed a higher amount of grade 1–2 AEs compared to ECF (diarrhea and neuropathy). Pre-operative TCF was associated with a higher incidence of grade 3–4 AEs compared to FLOT (anemia, febrile neutropenia, anorexia). Grade 1–2 AEs were higher with the FLOT regimen (mainly gastrointestinal toxicity). A full overview of significant differences in toxicity between the aforementioned regimens can be found in (Supplementary Tables 2, 3).

Post-operative ECF was not associated with an increase in grade 3–4 adverse events compared to CF+RT. However, post-operative CF+RT showed less grade 1–2 toxicity (neutropenia, mucositis, hand foot syndrome) compared to post-operative ECF. There was no toxicity data available on post-operative treatment with FLOT, TCF, FOx or CF.

**Figure 4:**
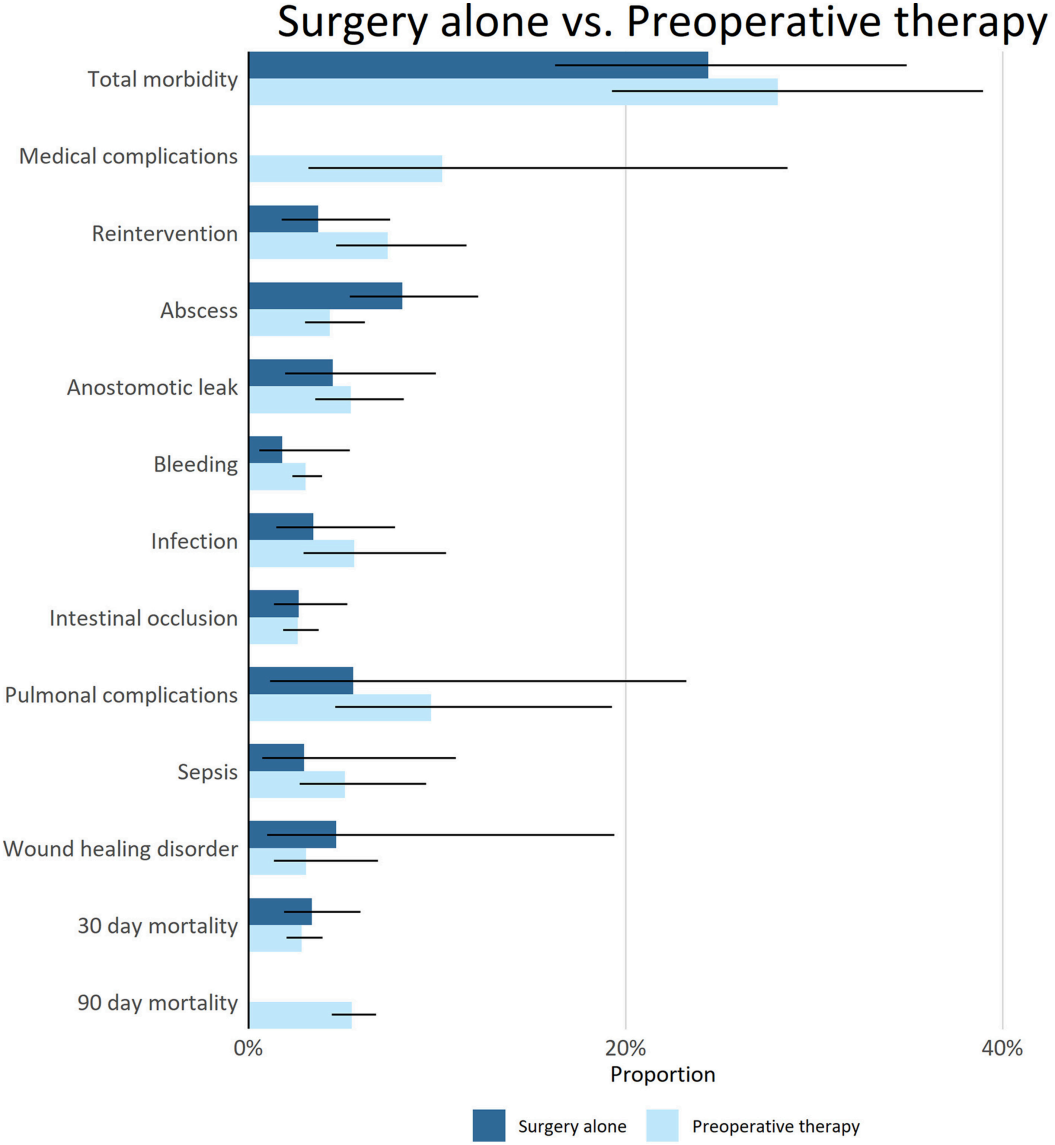
Surgical morbidity and mortality in patients treated with pre-operative therapy or with surgery alone. In the column on the left of the figure surgical outcomes are mentioned per group of bar charts. The bars in the figure give the pooled estimate with 95% CI (line in black in the bar). The color of the bar chart indicates if patients received pre-operative therapy before surgery. There was not enough information to make a distinction in severity of the surgical complications. For surgery alone there was no information available on the amount of medical complications and 90 day mortality.

Overall, a pooled analysis of patients randomized to a pre-operative therapy arm did not reveal any significant increase in surgery related morbidity/mortality compared to patients who underwent immediate surgery (Figure 4). An exploratory analysis was performed between several different pre-operative regimens; patients who received pre-operative CF experienced significantly less total surgery related morbidity compared to pre-operative FLOT and pre-operative ECF.

### TOXplot for Adjuvant Therapy

For 19 adjuvant regimens, we constructed bar charts with confidence intervals for AEs. Comparisons were made between FOx and CF, F, F+RT, or TF. Compared to FOx, the regimens CF and F+RT showed higher incidences of grade 3–4 AEs (stomatitis, anorexia, fatigue, neutropenia). Compared to F monotherapy and the doublet TF, the doublet FOx showed higher incidences of grade 3–4 adverse events (hematological toxicity and neuropathy). TF was also associated with a reduction in grade 1–2 AEs compared to FOx (Supplementary Table 4).

**Figure 5:**
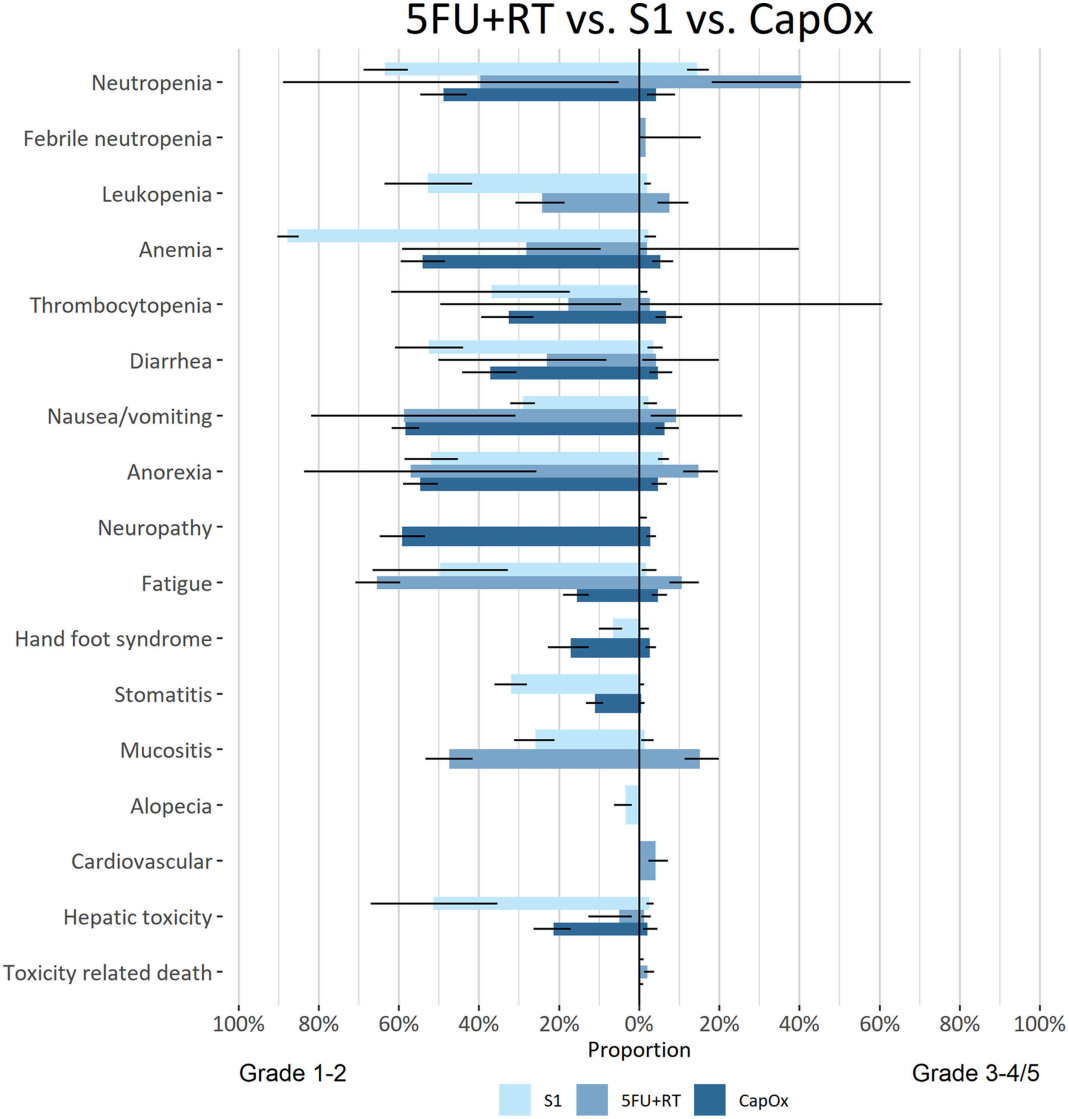
Bidirectional comparative meta-analysis of adjuvant S-1, CAPOX or 5-FU+RT in patients who did not receive pre-operative therapy. In the column on the left of the figure the adverse events are mentioned per group of bar charts. The bars in the figure give the pooled estimate with 95% CI (line in black in the bar). Grade 1–2 AEs are depicted on the left of the figure and grade 3–4/5 on the right of the figure. The color of the bar chart indicates which regimen is depicted.

To investigate regimens based on individual compounds; S-1 monotherapy, 5-FU+RT, and CAPOX were separately compared (Figure 5). In terms of grade 3–4 adverse events, 5-FU+RT was significantly more toxic than CAPOX and S-1 monotherapy (hematological toxicity, anorexia fatigue, mucositis). Treatment with S-1 monotherapy was associated with more grade 1–2 adverse events compared to CAPOX and 5-FU+RT (Supplementary Table 5).

### Heterogeneity

For several pooled proportions with more than one RCT significant (*p* < 0.05) heterogeneity was observed using the *Q*-test. The *I*^2^ values of these pooled AEs in the individual or bidirectional comparative COMplots ranged from 53 to 99%.

## Discussion

In this article, we have presented a novel overview of toxicity and surgical complications for the curative treatment of gastric cancer. The method in this paper is based on an article we published earlier on the toxicity profiles of first line chemotherapy in advanced gastroesophageal cancer (64). We conducted multiple random effect meta-analyses, based on individual treatment arms from RCTs. Using COMplot, we constructed a graphical presentation with pooled proportions and confidence intervals. Based on the performed analyses, we conclude that pre-operative therapy is not associated with an increase in surgery related morbidity or mortality compared to surgery alone. Pre-operative treatment with FLOT chemotherapy is not associated with a large increase in grade 3–4 AEs compared to pre-operative ECF, CF, or FOx. For adjuvant regimens, S-1 is associated with fewer grade 3–4 adverse events compared to CAPOX and 5-FU+RT.

A systematic review on shared decision making, across multiple types of cancer, found that in 19 out of 22 studies patients preferred a more active role regarding treatment decisions (65). The review highlighted that innovative interventions regarding improvement of shared decision making are lacking (65). For clinicians and patients, shared decision making can result in improved satisfaction with oncology care and communication with the physician (11). Unexpected and unrealistic views of patients on adverse effects of systemic treatment and surgical complications can result in decreased confidence in medical care, negative coping, and a deterioration in quality of life. It is well-known from phase I trials, that patients underestimate the potential toxicities that could result from oncological therapy (66). To improve awareness, recent efforts have focused on incorporating online information tools in oncology care. For example, an interactive online decision tool developed for breast cancer patients improved knowledge and preparation regarding treatment decisions (67). COMplots provide the physician with a graphical tool that could potentially facilitate the exchange of information on treatment effects between physician and patient. The data from COMplots and the method of analysis could be used in future online decision tools (68).

Graphical presentation of adverse effects of multimodality treatment is not yet available for use during consultation. COMplots provide pooled proportions with confidence intervals to give realistic estimates of the chance of occurrence of a certain adverse event. For clinicians, it can help in giving realistic estimates of the expected AEs (morbidity and mortality) regarding treatment with chemotherapy and surgery. Higher grade adverse events are deemed more acceptable to achieve curation. Therefore, clinicians might underestimate the value of informed decision making in the curative setting. However, even elderly curatively treated patients prefer an active or shared role above a passive role in oncological treatment decisions (69). Moreover, patients with a low health related quality of life reported more interest in shared decision making regarding cancer treatment (70). Clinicians can potentially use COMplots to actively engage patients in multimodality treatment decisions. Especially, for patients with co-morbidity or elderly patients COMplot can provide the means to weigh benefit of treatment, between regimens, or estimate the risks of undergoing major surgery. However, for this specific patient group, it should be realized that the estimates of adverse events from this pooled analysis are overall estimates and are not corrected for age or co-morbidity, these factors generally lead to an increase in toxicity.

In our COMplots we have performed several meta-analyses based on data from RCTs regarding the curative treatment of gastric cancer. Pre-operative therapy was not associated with an increase in surgery related morbidity or mortality compared to surgery alone. In several types of cancer, including esophageal and pancreatic cancer, neoadjuvant therapy was not associated with an increase in surgery related morbidity or mortality (71–73). Although, there is also evidence indicating the location and extent of the planning target volume of pre-operative radiotherapy might increase post-operative morbidity in esophageal cancer (74, 75). Ongoing pre-operative trials for gastric cancer like the CRITICS-2 trial (NCT02931890) should take this into account when pre-operative chemoradiotherapy is given. Our meta-analysis primarily included pre-operative chemotherapy studies and only one pre-operative chemoradiotherapy study and could thus not effectively rule out an effect of chemoradiotherapy on post-operative morbidity. Moreover, due to the high degree of heterogeneity in studies: Asian vs. Western, surgical techniques, extent of lymph node dissection, no definite conclusions can be drawn on the impact of individual regimens on surgical outcomes. Therefore, our finding that pre-operative CF was associated with less surgery related morbidity compared to FLOT and ECF should be regarded as exploratory and should be further investigated.

Treatment with pre-operative FLOT chemotherapy was associated with a small increase in AEs compared to pre-operative ECF, FOx, and CF in the COMplot meta-analysis. In the FLOT-4 trial perioperative FLOT substantially improved overall survival compared to perioperative ECF for gastric cancer (13). Therefore, patients treated with curative intent in good condition should receive perioperative FLOT over ECF, FOx, and CF, as only a minor increase in AEs was found. Pre-operative doublet chemotherapy should be reserved for patients with treatment limiting co-morbidity.

Patients who receive an immediate resection and are eligible for adjuvant treatment experience less grade 3–4 AEs with S-1 monotherapy compared to CAPOX and 5-FU with radiotherapy. Therefore, S-1 monotherapy might be more attractive for patients with co-morbidity. However, it must be noted adjuvant S-1 has only been investigated in curatively resected Asian patients. Effectivity in Western patients or patients with co-morbidity has not yet been investigated.

## Strengths and Limitations

The main strength of COMplot is the graphical presentation of toxicity and surgery related outcomes through pooled proportion meta-analysis with confidence intervals. Moreover, data can easily be interpreted as the number of studies and patients is given for each pooled treatment arm. Data on which the individual COMplots are based, have been obtained from RCTs were adverse events have been systematically scored, using the CTCAE classification.

However, COMplots also have several limitations. First, the adverse events are scored according to their maximum grade in the RCTs (76). There is no information available on the duration of an adverse event and the impact on quality of life. A recent paper incorporated longitudinal data in graphic tables and histograms of two RCTs (77). For COMplots this was not possible as the included RCTs do not provide longitudinal data on toxicity over time. In the future, RCTs should include toxicity over time analyses and provide data on quality of life, also during curative treatment.

Second, trials only report adverse events which occur over a certain threshold (for example in 5% of all patients) and surgery related morbidity was, for most trials, only reported within 30 days after surgery. For a small amount of toxicity events, the COMplots underestimate occurrence. Moreover, the long-term morbidity or deterioration of quality of life is not incorporated in the COMplots. Large prospective cohorts can provide more accurate incidences of adverse events and provide data on long term morbidity after surgery (78).

Third, cross-study comparisons between perioperative and adjuvant trials was not possible due to heterogeneity in baseline characteristics. For example, patients in adjuvant trials were mostly included after a R0 resection. Patients receiving post-operative chemotherapy in a perioperative trial were already pre-exposed to chemotherapy which could increase the likelihood of experiencing an AE.

## Conclusion

COMplots were constructed for clinically relevant regimens for the curative treatment of gastric cancer. The COMplots could potentially be used to inform patients about adverse events related to multimodality treatment.

Based on our meta-analysis, pre-operative FLOT only showed a minor increase in AEs compared to pre-operative doublet or triplet regimens. Therefore, pre-operative FLOT should be the preferred regimen in the perioperative setting for fit patients. Surgical outcomes are not impaired by pre-operative chemotherapy and can thus be safely administered. Ongoing trials will shed more light on the impact of pre-operative chemoradiotherapy on surgical outcomes as there is not enough data on this yet. In the adjuvant setting, S-1 monotherapy had a more favorable toxicity profile compared to CAPOX and 5-FU with RT and could thus be an more attractive option for patients with co-morbidity limiting more intensive treatment.

## Data Availability

Publicly available datasets were analyzed in this study. This data can be found here https://www.ncbi.nlm.nih.gov/pubmed/.

## Author Contributions

TvdE and FA performed the search for the review, extracted data, and analyzed the results. HvdB and EtV devised the method of analysis. HvdB created the R software package to create the COMplots and perform a meta-analysis. TvdE wrote the manuscript. EtV, MvO, and HvL provided intellectual guidance and corrected several draft versions. SG and MH provided intellectual input and corrected the final draft.

### Conflict of Interest Statement

SG has served as a consultant for Medtronic and has received an unrestricted research grant from Olympus. MvO has received unrestricted research grants from Bayer, Lilly, Merck Serono, and Roche. HvL has served as a consultant for Philips, Celgene, Lilly, and Nordic, and has received unrestricted research funding from Philips, Bayer, BMS, Celgene, Lilly, Merck Serono, MSD, Nordic, and Roche. The remaining authors declare that the research was conducted in the absence of any commercial or financial relationships that could be construed as a potential conflict of interest.
