# Supplementary material for: COMplot, A Graphical Presentation of Complication Profiles and Adverse Effects for the Curative Treatment of Gastric Cancer: A Systematic Review and Meta-Analysis
Source: Front Oncol. 2019 Jul 25;9:684. doi: 10.3389/fonc.2019.00684 (PMC6677173; doi:10.3389/fonc.2019.00684)
Supplement: Supplementary file 1 [file Data_Sheet_1.docx]

Supplementary Appendix

This appendix has been provided by the authors to give readers additional information about their work.

Supplement to: van den Ende T, Abe Nijenhuis FA, van den Boorn HG, et al.

# **Supplementary Methods**

**Cochrane Central Register of Controlled Trials (CENTRAL)**

| # | Searches |
| --- | --- |
| 1 | MeSH descriptor: [Esophageal Neoplasms] explode all trees |
| 2 | MeSH descriptor: [Stomach Neoplasms] explode all trees |
| 3 | (gastric or stomach or esophagus or oesophagus or esophageal or oesophageal) near (neoplasm* or cancer* or carcinoma or adenocarcino* or tumo?r* or malign* neoplasm* neoplasm*):ti,ab,kw (Word variations have been searched) |
| 4 | #1 or #2 or #3 |
| 5 | (chemotherapy or radiotherapy or chemoradiotherapy or radiation or radiochemotherapy) near/1 (adjuvant or neoadjuvant or neo-adjuvant or combined or perioperative or peri-operative or preoperative or pre-operative or postoperative or post-operative):ti,ab,kw (Word variations have been searched) |
| 6 | MeSH descriptor: [Gastrectomy] explode all trees |
| 7 | MeSH descriptor: [Esophagectomy] explode all trees |
| 8 | MeSH descriptor: [Chemotherapy, Adjuvant] explode all trees |
| 9 | MeSH descriptor: [Radiotherapy, Adjuvant] explode all trees |
| 10 | MeSH descriptor: [Chemoradiotherapy] explode all trees |
| 11 | MeSH descriptor: [Radiotherapy] explode all trees |
| 12 | #5 or #6 or #7 or #8 or #9 or #10 or #11 |
| 13 | MeSH descriptor: [Treatment Outcome] explode all trees |
| 14 | MeSH descriptor: [Disease-Free Survival] explode all trees |
| 15 | MeSH descriptor: [Mortality] explode all trees |
| 16 | survival or safe* or mortality or quality of life or QOL:ti,ab,kw (Word variations have been searched) |
| 17 | MeSH descriptor: [Quality of Life] explode all trees |
| 18 | #13 or #14 or #15 or #16 or #17 |
| 19 | #4 and #12 and #18 in Trials |

**EMBASE via Ovid**

| **#** | **Searches** |
| --- | --- |
| 1 | exp *esophagus tumor/ or exp *stomach tumor/ or ((gastric or stomach or esophagus or oesophagus or esophageal or oesophageal) adj (neoplasm* or cancer* or carcinoma or adenocarcino* or tumo?r* or malign* neoplasm* neoplasm*)).ti,ab,kw. |
| 2 | gastrectomy/ or *esophagus resection/ or *lymph node dissection/ or exp cancer adjuvant therapy/ or adjuvant chemoradiotherapy/ or cancer radiotherapy/ or gastrectomy.ti,ab,kw. or (exp chemoradiotherapy/ and adjuvant therapy/) or ((chemotherapy or radiotherapy or chemoradiotherapy or radiation or radiochemotherapy) adj1 (adjuvant or neoadjuvant or perioperative or peri-operative or preoperative or pre-operative or postoperative or post-operative)).ti,ab,kw. |
| 3 | controlled clinical trial/ or randomized controlled trial/ or "clinical trial (topic)"/ or (randomized or randomised or randomly).ti,ab,kw. or (trial or effecti*).ti. |
| 4 | exp treatment outcome/ or exp "quality of life"/ or disease-free survival/ or exp mortality/ or (survival or safe* or mortality or quality of life or QOL).ti,ab,kw. |
| 5 | 1 and 2 and 3 |
| 6 | "review"/ not "clinical trial (topic)"/ |
| 7 | lung.ti. |
| 8 | (1 and 2 and 3 and 4) not 6 not 7 |
| 9 | limit 8 to (dutch or english) |

**Medline via PubMed**

| **#** | **Searches** |
| --- | --- |
| 1 | esophageal neoplasms/ or stomach neoplasms/ or ((gastric or stomach or esophagus or oesophagus or esophageal or oesophageal) adj (neoplasm* or cancer* or carcinoma or adenocarcino* or tumo?r* or malign* neoplasm* neoplasm*)).ti,ab,kw. |
| 2 | exp Gastrectomy/ or Esophagectomy/ or Lymph Node Excision/ or Chemotherapy, Adjuvant/ or Radiotherapy, Adjuvant/ or gastrectomy.ti,ab,kw. or ((exp Chemoradiotherapy/ or exp Radiotherapy/) and (Neoadjuvant Therapy/ or adjuvant or neoadjuvant or neo-adjuvant).ti,ab,kw.) or ((chemotherapy or radiotherapy or chemoradiotherapy or radiation or radiochemotherapy) adj1 (adjuvant or neoadjuvant or neo-adjuvant or combined or perioperative or peri-operative or preoperative or pre-operative or postoperative or post-operative)).ti,ab,kw. |
| 3 | exp treatment outcome/ or "Quality of Life"/ or disease-free survival/ or exp Mortality/ or (survival or safe* or mortality or quality of life or QOL).ti,ab,kw. |
| 4 | controlled clinical trial/ or randomized controlled trial/ or Clinical Trials as Topic/ or (randomi?ed or randomly).ti,ab,kw. or (trial or effecti*).ti. |
| 5 | 1 and 2 and 3 and 4 |
| 6 | "review"/ not Clinical Trials as Topic/ |
| 7 | lung.ti. |
| 8 | (1 and 2 and 3 and 4) not 6 not 7 |
| 9 | limit 8 to (dutch or english) |

**Conference search: American Society of Clinical Oncology**

Searching journal content for gastric or esophageal (all words) in title or abstract and random* in full text, from earliest publication date through May 2019.

**Conference search: European Society of Medical Oncology**

Searching journal content for gastric or esophageal (all words) in title or abstract and random* in full text, from earliest publication date through May 2019.

**Supplementary table 1.** Overview of included studies with dosing of cytotoxic-agents and radiation therapy dosage

| **Studies** | **Dose Arm A** | **Dose Arm B** | **Dose Arm C and/or Arm D** | **Concurrent radiotherapy** |
| --- | --- | --- | --- | --- |
| *Al-Batran 2019[1]* | Six perioperative cycles: epirubicin 50 mg/m² day 1; cisplatin 60 mg/m² day 1; 5-FU 200 mg/m² days 1-21 or capecitabine 1250 mg/m² days 1-21 | Eight perioperative cycles: docetaxel 50 mg/m² day 1; oxaliplatin 85 mg/m² day 1; leucovorin 200 mg/m² day 1; 5-FU 2600 mg/m² day 1 |  |  |
| *Aoyama 2017[2]* | Two preoperative cycles: S-1 80 mg/m^2^ daily for 21 days every 4 weeks, cisplatin 60 mg/m^2^ day 8 every 4 weeks | Four preoperative cycles: S-1 80 mg/m^2^ daily for 21 days every 4 weeks, cisplatin 60 mg/m^2^ day 8 every 4 weeks | Arm C: Two preoperative cycles: S-1 80 mg/m^2^ daily for 14 days every 4 weeks, cisplatin 60 mg/m^2^ day 1 every 4 weeks, docetaxel 40 mg/m^2^ day 1, every 4 weeks  Arm D: Four preoperative cycles: S-1 80 mg/m^2^ daily for 14 days every 4 weeks, cisplatin 60 mg/m^2^ day 1 every 4 weeks, docetaxel 40 mg/m^2^ day 1, every 4 weeks |  |
| *Bajetta 2002[3]* | Two cycles: etoposide 120 mg/m² days 4-6; adriamycin 20 mg/m² days 1, 7; cisplatin 40 mg/m² days 2, 8, followed by two cycles: 5-FU 375 mg/m² days 1-5; leucovorin 100 mg/m² days 1-5 |  |  |  |
| *Bajetta 2014 [4]* | Four cycles: 5-FU 400-600 mg/m² days 1-2; leucovorin 100 mg/m² days 1-2; irinotecan 180 mg/m² day 1, followed by three cycles: docetaxel 75 mg/m² day 1; cisplatin 75 mg/m² day 1 | Nine cycles: 5-FU 400-600 mg/m² days 1-2; leucovorin 100 mg/m² days 1-2 |  |  |
| *Bamias 2010[5]* | Six cycles: docetaxel 75 mg/m² day 1; cisplatin 75 mg/m² or carboplatin (AUC5) | Six cycles: docetaxel 75 mg/m² day 1; cisplatin 75 mg/m² or carboplatin (AUC5) with RT |  | ARM B: 1.8 Gy days 1-5: total 45 Gy |
| *Bartolomeo 2006[6]* | Four cycles: 5-FU 400-600 mg/m² days 1-2; leucovorin 100 mg/m² days 1-2; irinotecan 180 mg/m² day 1, followed by three cycles: docetaxel 75 mg/m² day 1; cisplatin 75 mg/m² day 1 | Four cycles: mytomycin C 8 mg/m² days 1-2 |  |  |
| *Bouche 2005[7]* | 5-FU 800 mg/m² days 1-5, followed by four cycles 5-FU 1000 mg/m² days 1-5; cisplatin 100 mg/m² day 2 |  |  |  |
| *Cascinu 2007[8]* | Six cycles: 5-FU 375 mg/m² days 1-5; leucovorin 20 mg/m² days 1-5 | Eight cycles: cisplatin 40 mg/m² day 1; 5-FU 500 mg/m² day 1; leucovorin 250 mg/m² day 1 |  |  |
| *Cats 2018[9]* | Six perioperative cycles: epirubicin 50 mg/m² day 1; cisplatin 60 mg/m² or oxaliplatin 130 mg/m² day 1; capecitabine 2000 mg/m² days 1-14 | Three preoperative cycles: epirubicin 50 mg/m² day 1; cisplatin 60 mg/m² or oxaliplatin 130 mg/m² day 1; capecitabine 2000 mg/m² days 1-14; followed by postoperative capecitabine 1150 mg/m² daily; cisplatin 20 mg/m² day 1 with RT |  | ARM B: 1.8 Gy days 1-5: total 45 Gy |
| *Chang 2002[10]* | Twelve cycles: 5-FU 500 mg/m² days 1-5 | Six cycles: 5-FU 350 mg/m² days 1-5; mytomycin C 10 mg/m² every other day 1 | ARM C: Six cycles: 5-FU 600 mg/m² days 1, 8, 29, 35; doxorubicin 30 mg/m² days 1, 29; mytomycin C 10 mg/m² every other day 1 |  |
|  |  |  | ARM D: Six cycles: 5-FU 600 mg/m² days 1, 8, 29, 35; doxorubicin 30 mg/m² days 1, 29; mytomycin C 10 mg/m² every other day 1 |  |
|  |  |  |  |  |
|  |  |  |  |  |
| *Chipponi 2004[11]* | Nine cycles: 5-FU 375-500 mg/m² days 1-5; leucovorin 200 mg/m² days 1-5; cisplatin 15 mg/m² days 1-5 |  |  |  |
| *Cirera 1999[12]* | Three cycles: mytomycin C 20 mg/m² day 1; tegafur 400 mg/m² days 1-30 |  |  |  |
| *Coombes 1990[13]* | Six cycles: 5-FU 600mg/m² days 1, 8, 29, 36; doxorubicin 30 mg/m² days 1, 29; mytomycin C 10 mg/m² day 1 |  |  |  |
| *Cunningham 2006[14]* | Six perioperative cycles: epirubicin 50 mg/m² day 1; cisplatin 60 mg/m² day 1; capecitabine 1250 mg/m² days 1-21 |  |  |  |
| *Cunningham 2017[15]* | Six perioperative cycles: epirubicin 50 mg/m² day 1; cisplatin 60 mg/m² day 1; 5-FU 200 mg/m² days 1-21 | Six perioperative cycles: epirubicin 50 mg/m² day 1; cisplatin 60 mg/m² day 1; 5-FU 200 mg/m² days 1-21, with twelve cycles bevacizumab 7.5 mg/kg day 1 |  |  |
| *De Vita 2007[16]* | Six cycles: 5-FU 375 mg/m² days 1-5; leucovorin 100 mg/m² days 1-5; epirubicin 60 mg/m² day 1; etoposide 80 mg/m² days 1-3 |  |  |  |
| *Di Costanzo 2008[17]* | Four cycles: 5-FU 300 mg/m² days 1-4; leucovorin 100 mg/m² days 1-4; cisplatin 40 mg/m² days 1, 5; epirubicin 30 mg/m² days 1, 5 |  |  |  |
| *Fazio 2016[18]* | Four preoperative cycles: 5-FU 300 mg/m² days 1-14; docetaxel 75 mg/m² day 1; cisplatin 75 mg/m² day 1 | Four adjuvant cycles: 5-FU 300 mg/m² days 1-14; docetaxel 75 mg/m² day 1; cisplatin 75 mg/m² day 1 |  |  |
| *Feng 2015[19]* | Eight cycles: oxaliplatin 130 mg/m² day 1; capecitabine 2000 mg/m² days 1-14 | Sixteen cycles: oxaliplatin 130 mg/m² day 1, cycles 1-8; capecitabine 2000 mg/m² days 1-14, cycles 1-16 |  |  |
| *Fuchs 2017[20]* | Three cycles: 5-FU 425 mg/m² days 1-5; leucovorin 20 mg/m² days 1-5 | Three cycles: 5-FU 200 mg/m² days 1-21; epirubicin 50 mg/m² day 1; cisplatin 60 mg/m² day 1 with RT |  | ARM B: 1.8 Gy days 1-5: total 45 Gy |
| *Hartgrink 2004[21]* | Four preoperative cycles: methotrexate 1500 mg/m² day 1; 5-FU 1500 mg/m² day 1; leucovorin 120 mg/m² days 2-3; doxorubicin 30 mg/m² day 15 |  |  |  |
| *Imano 2010[22]* | One preoperative cycle: 5-FU 330 mg/m²/24h for 72h | Three preoperative cycles: cisplatin 6 mg/m² | One preoperative cycle: 5-FU 330 mg/m²/24h for 72h Three preoperative cycles: cisplatin 6 mg/m² |  |
| *Kang 2013[23]* | Three cycles: doxifluridine 460-600 mg/m² days 1-28; mytomycin C 20 mg/m² day 1 | Twelve cycles: doxifluridine 460-600 mg/m² days 1-28; cycles 1-6: mytomycin C 20 mg/m² day 1; cisplatin 60 mg/m² day 1 |  |  |
| *Kim 2012[24]* | Five cycles: 5-FU 425 mg/m² days 1-5; leucovorin 20 mg/m² days 1-5 | Five cycles: 5-FU 400-425 mg/m² days 1-5; leucovorin 20 mg/m² days 1-5 with RT |  | ARM B: 1.8 Gy days 1-5: total 45 Gy |
| *Krook 1991[25]* | Three cycles: 5-FU 350 mg/m² days 1-5; doxorubicin 40 mg/m² day 1 |  |  |  |
| *Kulig 2010[26]* | Three cycles: doxorubicin 20 mg/m² days 1, 7; cisplatin 40 mg/m² days 2, 8; etoposide 120 mg/m² days 4-6 |  |  |  |
| *Kwon 2010[27]* | Six cycles: 5-FU 1000 mg/m² days 1-5; cisplatin 60 mg/m² day 1 | Six cycles: 5-FU 1000 mg/m² days 1-5; cisplatin 60 mg/m² day 1, interrupted by five cycles RT+capecitabine 3300 mg/m² days 1-5 |  | ARM B: 1.8 Gy days 1-5: total 45 Gy |
| *Lee 2004[28]* | Six cycles: 5-FU 500 mg/m² days 1-4 | Two cycles: 5-FU 300 mg/m² days 1-4; leucovorin days 1-4; cisplatin 40 mg/m² days 1, 5; epirubicin 30 mg/m² days 1, 5 followed by four cycles: epirubicin 75 mg/m² day 1; 5-FU 450 mg/m² days 1-3; leucovorin 20 mg/m² days 1-3 |  |  |
| *Lee 2018[29]* | Eight cycles: docetaxel 35 mg/m² day 1, 8; S-1 70 mg/m² days 1-14 | Eight cycles: cisplatin 60 mg/m² day 1, 8; S-1 70 mg/m² days 1-14 |  |  |
| *Leong 2017[30]* | Six perioperative cycles: epirubicin 50 mg/m² day 1; cisplatin 60 mg/m² day 1; 5-FU 200 mg/m² day 1-21 | Two preoperative with RT and three postoperative cycles: epirubicin 50 mg/m² day 1; cisplatin 60 mg/m² day 1; 5-FU 200 mg/m² day 1-21, preoperative followed by five cycles: 5-FU 200 mg/m² daily |  | ARM B: preoperative 1.8 Gy days 1-5: total 45 Gy |
| *Lise 1995[31]* | Seven cycles: 5-FU 400 mg/m² days 1-3, 22-24; doxorubicin 40 mg/m² days 2, 23; mytomycin C 10 mg/m² day 1 |  |  |  |
| *Nakajima 1999[32]* | Three cycles: 5-FU 166.7 mg/m² twice weekly; mytomycin C 1.4 mg/m² twice weekly, followed by UFT 300 mg/m² daily for 18 months |  |  |  |
| *Nakajima 2007[33]* | 64 cycles: UFT 360 mg/m² days 1-5 |  |  |  |
| *Neri 2001[34]* | Seven cycles: 5-FU 450 mg/m² days 1-3; leucovorin 200 mg/m² days 1-3; epirubicin 75 mg/m² day 1 |  |  |  |
| *Nitti 2006[35]* | Six cycles: 5-FU 1500 mg/m² days 2-4; leucovorin 15 mg/m² days 2-4; doxorubicin 30 mg/m² day 15; methotrexate 1500 mg/m² day 1 | Six cycles: 5-FU 1500 mg/m² days 2-4; leucovorin 15 mg/m² days 2-4; epirubicin 70 mg/m² day 15; methotrexate 1500 mg/m² day 1 |  |  |
| *Noh 2014[36]* | Eight cycles: oxaliplatin 130 mg/m² day 1; capecitabine 2000 mg/m² days 1-14 |  |  |  |
| *Park 2015[37]* | Six cycles: cisplatin 60 mg/m² day 1; capecitabine 2000 mg/m² days 1-14 | Two cycles: cisplatin 60 mg/m² day 1; capecitabine 2000 mg/m² days 1-14, followed by five cycles capecitabine 1650 mg/m², followed by two cycles cisplatin 60 mg/m² day 1; capecitabine 2000 mg/m² days 1-14 with RT |  | ARM B: 1.8 Gy days 1-5: total 45 Gy |
| *Sasako 2011[38]* | Eight cycles: S-1 80-120 mg/m² days 1-28 |  |  |  |
| *Schuhmacher 2010[39]* | Two preoperative cycles: 5-FU 2000 mg/m² + leucovorin 500 mg/m² days 1, 8, 15, 22, 29, 36; cisplatin 50 mg/m² days 1, 15, 29 |  |  |  |
| *Schwartz 2009[40]* | Two cycles: 5-FU 600 mg/m² days 1-5, 29-33; cisplatin 15 mg/m² days 1-5, 29-33; paclitaxel 175 mg/m² days 1, 29, followed by five cycles 5-FU 300 mg/² days 1-5; paclitaxel 45 mg/m² day 1 with RT | Two cycles: cisplatin 75 mg/m² days 1, 29; paclitaxel 175 mg/m² days 1, 29, followed by five cycles: paclitaxel 60 mg/m² days 1-5; cisplatin 30 mg/m² day 1 with RT |  | ARM A+B: 1.8 Gy days 1-5: total 45 Gy |
| *Smalley 2012[41]* | Four cycles: 5-FU 400-425 mg/m² days 1-5; leucovorin 20 mg/m² days 1-5 with RT |  |  | ARM A: 1.8 Gy days 1-5: total 45 Gy |
| *Tentes 2006[42]* | Three cycles: 5-FU 600 mg/m² day 1; doxorubicin 15 mg/m² day 2; mytomycin C 7 mg/m² day 3 |  |  |  |
| *Terashima 2019[43]* | Adjuvant S-1 day 80-120 mg/m^2^ days 1-28 of 6 week cycle | Preoperative: S-1 80-120 mg/m^2^ days 1-21 of a 4 week course, cisplatin 60 mg/m^2^ on day 8. Adjuvant S-1 day 80-120 mg/m^2^ days 1-28 of 6 week cycle |  |  |
| *Tsavaris 1996[44]* | Three cycles: 5-FU 600 mg/m² day 1, 8, 29, 36; epirubicin 45 mg/m² day 1, 29; mytomycin C 10 mg/m² day 1 |  |  |  |
| *Tsuburaya 2014[45]* | Twelve cycles: UFT 267 mg/m² days 1-28 | Sixteen cycles: S-1 360 mg/m² days 1-14 | ARM C: Three cycles: paclitaxel 80 mg/m² days 1, 8, 15; followed by nine cycles of UFT 267 mg/m² days 1-28 |  |
|  |  |  | ARM D: Three cycles: paclitaxel 80 mg/m² days 1, 8, 15; followed by twelve cycles of S-1 180 mg/m² days 1-14 |  |
|  |  |  |  |  |
|  |  |  |  |  |
| *Tsujinaka 2000[46]* | Four cycles mytomycin C: 60 mg/m² day 1; Oral FU 200 mg days 1-365 | Four cycles: epirubicin 30 mg/m² day 1; Oral FU 200 mg days 1-365 | Oral FU 200 mg days 1-365 |  |
| *Xue 2018[47]* | Eight perioperative cycles: S-1 80 mg/m^2^ day 1-14, oxaliplatin 130 mg/m^2^ day 1 | Eight perioperative cycles: capecitabine 1000 mg/m^2^ day 1-14, oxaliplatin 130 mg/m^2^ day 1 | ARM C: Eight adjuvant cycles: S-1 80 mg/m^2^ day 1-14, oxaliplatin 130 mg/m^2^ day 1  Arm D: Eight adjuvant cycles: capecitabine 1000 mg/m^2^ day 1-14, oxaliplatin 130 mg/m^2^ day 1 |  |
| *Ychou 2011[48]* | Two to three preoperative cycles: 5-FU 800 mg/m² days 1-5; cisplatin 100 mg/m² day 1 followed by three to four postoperative cycles: 5-FU 600 mg/m² days 1-5; cisplatin 100 mg/m² day 1 |  |  |  |
| *Yoshikawa 2016[49]* | Two or four preoperative cycles: S-1 80 mg/m² days 1-21; cisplatin 60 mg/m² day 8 | Two or four preoperative cycles: paclitaxel 80 mg/m² days 1, 8, 15; cisplatin 25 mg/m² day 1, 8, 15 |  |  |
| *Yoshikawa 2019[50]* | Eight cycles: S-1 80-120 mg/m² days 1-28 | Four cycles: S-1 80-120 mg/m² days 1-28 |  |  |
| *Yu 2012[51]* | Five cycles: 5-FU 400-425 mg/m² days 1-5; leucovorin 25 mg/m² days 1-5 | Four cycles: 5-FU 400-425 mg/m² days 1-5; leucovorin 20 mg/m² days 1-5 with RT |  | ARM B: 1.8 Gy days 1-5: total 45 Gy |
| *Zhang 2011[52]* | 5-FU 400-600 mg/m² days 1-2; leucovorin 200 mg/m² days 1-2; oxaliplatin 85 mg/m² day 1 | 5-FU 400-600 mg/m² days 1-2; leucovorin 200 mg/m² days 1-2; |  |  |
| *Zhao 2006[53]* | One cycle preoperative: doxifluridine 800-1200 mg days 3-5 | One cycle preoperative: 5-FU 500 mg days 3-5; 200 mg CF days 3-5 |  |  |
| *Zhao 2017[54]* | Eight perioperative cycles: S-1 40-60 mg/m^2^ day 1-14, oxaliplatin 130 mg/m^2^ on day 1 | Adjuvant chemotherapy: S-1 40-60 mg/m^2^ day 1-14, oxaliplatin 130 mg/m^2^ on day 1 |  |  |
| *Zhu 2012[55]* | Four cycles: 5-FU 400 mg/m² days 1-5; leucovorin 20 mg/m² days 1-5 | Four cycles: 5-FU 400 mg/m² days 1-5; leucovorin 20 mg/m² days 1-5 with RT |  | ARM B: 1.8 Gy days 1-5: total 45 Gy |


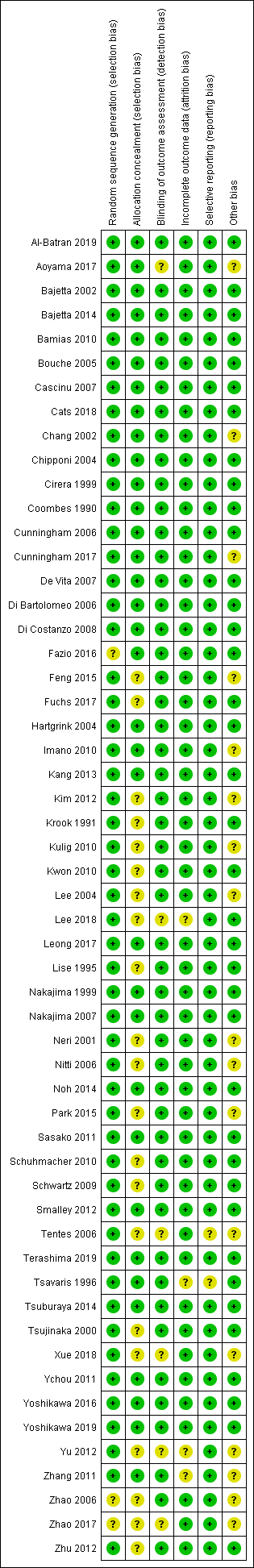
**Supplementary figure 1.** Risk of bias overview

Single-centre studies were scored as unknown risk of bias on the item ‘other biases’. += Low risk of bias; ?= unknown risk of bias

**Supplementary Table 2.** Significant differences between preoperative chemotherapy regimens (p<0.05).

|  | **Preoperative CF vs. FLOT** | | **Preoperative ECF vs. FLOT** | | **Preoperative CF vs. ECF** | |
| --- | --- | --- | --- | --- | --- | --- |
|  | CF>FLOT | FLOT>CF | ECF>FLOT | FLOT>ECF | CF>ECF | ECF>CF |
| Grade 1-2 | Neutropenia | Diarrhea | Hand foot syndrome | Diarrhea | Neutropenia | Fatigue |
|  |  | Fatigue |  | Neuropathy |  | Nausea/vomiting |
|  |  | Nausea/vomiting |  | Hepatic toxicity |  | Stomatitis |
|  |  | Stomatitis |  |  |  |  |
|  |  | Hepatic toxicity |  |  |  |  |
| Grade 3-4 | Anemia | Leukopenia |  | Leukopenia |  |  |
|  | Anorexia | Neutropenia |  | Neutropenia |  |  |
|  |  | Diarrhea |  | Hepatic toxicity |  |  |
|  |  |  |  | Neuropathy |  |  |

The greater than (>) sign indicates which regimen shows a significantly higher incidence in a specific adverse event compared to the other regimen based on the distinction in the first column grade 1-2 or grade 3-4. For example in column 2 CF showed a higher incidence in grade 1-2 neutropenia compared to FLOT.

Abbreviations: C, cisplatin; E, epirubicin; F, fluoropyrimidine; L, leucovorin; Ox, oxaliplatin; T, taxane (either paclitaxel or docetaxel)

**Supplementary Table 3.** Significant differences between preoperative chemotherapy regimens (p<0.05).

|  | **Preoperative TCF vs. FLOT** | | **Preoperative FOx vs. FLOT** | | **Preoperative FOx vs. CF** | |
| --- | --- | --- | --- | --- | --- | --- |
|  | TCF>FLOT | FLOT>TCF | FOx>FLOT | FLOT>FOx | FOx>CF | CF>FOx |
| Grade 1-2 | Anorexia | Diarrhea | Anorexia | Anemia |  | Anemia |
|  | Neutropenia | Fatigue |  | Diarrhea |  | Mucositis |
|  |  | Nausea/vomiting |  | Fatigue |  | Neutropenia |
|  |  | Hepatic toxicity |  | Nausea/vomiting |  | Hepatic toxicity |
|  |  |  |  | Neuropathy |  |  |
|  |  |  |  | Stomatitis |  |  |
|  |  |  |  | Hepatic toxicity |  |  |
| Grade 3-4 | Anemia |  |  | Leukopenia |  | Neutropenia |
|  | Febrile neutropenia |  |  | Neutropenia |  |  |
|  | Anorexia |  |  |  |  |  |

The greater than (>) sign indicates which regimen shows a significantly higher incidence in a specific adverse event compared to the other regimen based on the distinction in the first column grade 1-2 or grade 3-4. For example in column 2 TCF showed a higher incidence in grade 1-2 neutropenia and anorexia compared to FLOT.

Abbreviations: C, cisplatin; F, fluoropyrimidine; L, leucovorin; Ox, oxaliplatin; T, taxane (either paclitaxel or docetaxel)

**Supplementary Table 4.** Significant differences between adjuvant chemotherapy regimens (p<0.05).

|  | **FOx vs. CF** | | **FOx vs. F** | | **FOx vs. F+RT** | | **FOx vs. TF** | |
| --- | --- | --- | --- | --- | --- | --- | --- | --- |
|  | FOx>CF | CF>FOx | FOx>F | F>FOx | FOx>F+RT | F+RT>FOx | FOx>TF | TF>FOx |
| Grade 1-2 | Neuropathy | Stomatitis | Nausea/vomiting | Fatigue | Hepatic toxicity | Fatigue | Neuropathy | Alopecia |
|  |  |  |  |  |  |  | Neutropenia |  |
|  |  |  |  |  |  |  | Thrombocytopenia |  |
| Grade 3-5 |  | Neutropenia | Leukopenia |  |  | Toxicity related death | Leukopenia |  |
|  |  | Stomatitis | Anemia |  |  | Neutropenia | Anemia |  |
|  |  |  | Thrombocytopenia |  |  | Anorexia | Thrombocytopenia |  |
|  |  |  | Neuropathy |  |  | Fatigue | Nausea/vomiting |  |
|  |  |  |  |  |  |  | Neuropathy |  |

The greater than (>) sign indicates which regimen shows a significantly higher incidence in a specific adverse event compared to the other regimen based on the distinction in the first column grade 1-2 or grade 3-5. For example in column 2 FOx showed a higher incidence in grade 1-2 neuropathy compared to CF.

Abbreviations: C, cisplatin; F, fluoropyrimidine; Ox, oxaliplatin; RT, radiotherapy; T, taxane (either paclitaxel or docetaxel)

**Supplementary Table 5.** Significant differences between adjuvant chemotherapy regimens (p<0.05).

|  | **5-FU+RT vs. S1** | | **CAPOX vs. S1** | | **CAPOX vs. 5-FU+RT** | |
| --- | --- | --- | --- | --- | --- | --- |
|  | 5-FU+RT>S1 | S1>5-FU+RT | CAPOX>S1 | S1>CAPOX | CAPOX>5-FU+RT | 5-FU+RT>CAPOX |
| Grade 1-2 | Mucositis | Leukopenia | Hand/foot syndrome | Neutropenia | Hepatic toxicity | Fatigue |
|  |  | Anemia |  | Diarrhea |  |  |
|  |  | Hepatic toxicity |  | Fatigue |  |  |
|  |  |  |  | Stomatitis |  |  |
|  |  |  |  | Hepatic toxicity |  |  |
| Grade 3-5 | Neutropenia |  | Thrombocytopenia | Neutropenia |  | Neutropenia |
|  | Leukopenia |  | Neuropathy |  |  | Anorexia |
|  | Anorexia |  | Nausea/vomiting |  |  | Fatigue |
|  | Fatigue |  | Fatigue |  |  | Toxicity related death |
|  | Mucositis |  | Hand foot syndrome |  |  |  |
|  | Toxicity related death |  |  |  |  |  |

The greater than (>) sign indicates which regimen shows a significantly higher incidence in a specific adverse event compared to the other regimen based on the distinction in the first column grade 1-2 or grade 3-5. For example in column 2, 5-FU+RT showed a higher incidence of mucositis compared to S-1.

Abbreviations: 5-FU, Fluorouracil; CAP, capecitabine; OX, oxaliplatin; RT, radiotherapy

**References**

[1] S.E. Al-Batran, N. Homann, C. Pauligk, T.O. Goetze, J. Meiler, S. Kasper, H.G. Kopp, F. Mayer, G.M. Haag, K. Luley, U. Lindig, W. Schmiegel, M. Pohl, J. Stoehlmacher, G. Folprecht, S. Probst, N. Prasnikar, W. Fischbach, R. Mahlberg, J. Trojan, M. Koenigsmann, U.M. Martens, P. Thuss-Patience, M. Egger, A. Block, V. Heinemann, G. Illerhaus, M. Moehler, M. Schenk, F. Kullmann, D.M. Behringer, M. Heike, D. Pink, C. Teschendorf, C. Lohr, H. Bernhard, G. Schuch, V. Rethwisch, L.F. von Weikersthal, J.T. Hartmann, M. Kneba, S. Daum, K. Schulmann, J. Weniger, S. Belle, T. Gaiser, F.S. Oduncu, M. Guntner, W. Hozaeel, A. Reichart, E. Jager, T. Kraus, S. Monig, W.O. Bechstein, M. Schuler, H. Schmalenberg, R.D. Hofheinz, and F.A. Investigators, Perioperative chemotherapy with fluorouracil plus leucovorin, oxaliplatin, and docetaxel versus fluorouracil or capecitabine plus cisplatin and epirubicin for locally advanced, resectable gastric or gastro-oesophageal junction adenocarcinoma (FLOT4): a randomised, phase 2/3 trial. Lancet 393 (2019) 1948-1957.

[2] T. Aoyama, K. Nishikawa, K. Fujitani, K. Tanabe, S. Ito, T. Matsui, A. Miki, H. Nemoto, K. Sakamaki, T. Fukunaga, Y. Kimura, N. Hirabayashi, and T. Yoshikawa, Early results of a randomized two-by-two factorial phase II trial comparing neoadjuvant chemotherapy with two and four courses of cisplatin/S-1 and docetaxel/cisplatin/S-1 as neoadjuvant chemotherapy for locally advanced gastric cancer. Ann Oncol 28 (2017) 1876-1881.

[3] E. Bajetta, R. Buzzoni, L. Mariani, E. Beretta, F. Bozzetti, G. Bordogna, E. Aitini, S. Fava, G. Schieppati, G. Pinotti, M. Visini, G. Ianniello, and B.M. Di, Adjuvant chemotherapy in gastric cancer: 5-year results of a randomised study by the Italian Trials in Medical Oncology (ITMO) Group. Ann Oncol 13 (2002) 299-307.

[4] E. Bajetta, I. Floriani, M. Di Bartolomeo, R. Labianca, A. Falcone, F. Di Costanzo, G. Comella, D. Amadori, C. Pinto, C. Carlomagno, D. Nitti, B. Daniele, E. Mini, D. Poli, A. Santoro, S. Mosconi, R. Casaretti, C. Boni, G. Pinotti, P. Bidoli, L. Landi, G. Rosati, A. Ravaioli, M. Cantore, F. Di Fabio, E. Aitini, A. Marchet, and I.-S.S. Group, Randomized trial on adjuvant treatment with FOLFIRI followed by docetaxel and cisplatin versus 5-fluorouracil and folinic acid for radically resected gastric cancer. Ann Oncol 25 (2014) 1373-8.

[5] A. Bamias, M. Karina, P. Papakostas, I. Kostopoulos, M. Bobos, G. Vourli, E. Samantas, C. Christodoulou, G. Pentheroudakis, D. Pectasides, M.A. Dimopoulos, and G. Fountzilas, A randomized phase III study of adjuvant platinum/docetaxel chemotherapy with or without radiation therapy in patients with gastric cancer. Cancer Chemother Pharmacol 65 (2010) 1009-21.

[6] M. Di Bartolomeo, R. Buzzoni, L. Mariani, E. Ferrario, D. Katia, A. Gevorgyan, N. Zilembo, R. Bordonaro, A.M. Bochicchio, B. Massidda, A. Ardizzoia, G. Marini, E. Aitini, G. Schieppati, G. Comella, G. Pinotti, S. Palazzo, G. Cicero, E. Bajetta, G. Italian Trial in Medical Oncology, E. Villa, D. Fagnani, G. Reguzzoni, B. Agostana, C. Oliani, B. Kildani, M. Duro, M. Botta, R. Mozzana, and G. Mantovani, Feasibility of sequential therapy with FOLFIRI followed by docetaxel/cisplatin in patients with radically resected gastric adenocarcinoma. A randomized phase III trial.[Erratum appears in Oncology. 2007;73(5-6):406 Note: Ardizzoni, Antonio [corrected to Ardizzoia, Antonio]]. Oncology 71 (2006) 341-6.

[7] O. Bouche, M. Ychou, P. Burtin, L. Bedenne, M. Ducreux, G. Lebreton, J. Baulieux, B. Nordlinger, C. Martin, J.F. Seitz, J.M. Tigaud, E. Echinard, N. Stremsdoerfer, C. Milan, P. Rougier, and G. Federation Francophone de Cancerologie Digestive, Adjuvant chemotherapy with 5-fluorouracil and cisplatin compared with surgery alone for gastric cancer: 7-year results of the FFCD randomized phase III trial (8801). Ann Oncol 16 (2005) 1488-97.

[8] S. Cascinu, R. Labianca, C. Barone, A. Santoro, C. Carnaghi, A. Cassano, G.D. Beretta, V. Catalano, O. Bertetto, S. Barni, L. Frontini, E. Aitini, S. Rota, V. Torri, I. Floriani, C. Italian Group for the Study of Digestive Tract, C. Pozzo, L. Rimassa, S. Mosconi, P. Giordani, A. Ardizzoia, P. Foa, C. Rabbi, S. Chiara, G. Gasparini, M. Nardi, M. Mansutti, E. Arnoldi, E. Piazza, E. Cortesi, F. Pucci, R.R. Silva, A. Sobrero, and A. Ravaioli, Adjuvant treatment of high-risk, radically resected gastric cancer patients with 5-fluorouracil, leucovorin, cisplatin, and epidoxorubicin in a randomized controlled trial. J Natl Cancer Inst 99 (2007) 601-7.

[9] A. Cats, E.P.M. Jansen, N.C.T. van Grieken, K. Sikorska, P. Lind, M. Nordsmark, E. Meershoek-Klein Kranenbarg, H. Boot, A.K. Trip, H.A.M. Swellengrebel, H.W.M. van Laarhoven, H. Putter, J.W. van Sandick, M.I. van Berge Henegouwen, H.H. Hartgrink, H. van Tinteren, C.J.H. van de Velde, M. Verheij, and C. investigators, Chemotherapy versus chemoradiotherapy after surgery and preoperative chemotherapy for resectable gastric cancer (CRITICS): an international, open-label, randomised phase 3 trial. Lancet Oncol 19 (2018) 616-628.

[10] H.M. Chang, K.H. Jung, T.Y. Kim, W.S. Kim, H.K. Yang, K.U. Lee, K.J. Choe, D.S. Heo, Y.J. Bang, and N.K. Kim, A phase III randomized trial of 5-fluorouracil, doxorubicin, and mitomycin C versus 5-fluorouracil and mitomycin C versus 5-fluorouracil alone in curatively resected gastric cancer. Ann Oncol 13 (2002) 1779-85.

[11] J. Chipponi, M. Huguier, D. Pezet, N. Basso, J.M. Hay, P. Quandalle, D. Jaeck, P.L. Fagniez, and A. Gainant, Randomized trial of adjuvant chemotherapy after curative resection for gastric cancer. Am J Surg 187 (2004) 440-5.

[12] L. Cirera, A. Balil, E. Batiste-Alentorn, I. Tusquets, T. Cardona, A. Arcusa, L. Jolis, E. Saigi, I. Guasch, A. Badia, and M. Boleda, Randomized clinical trial of adjuvant mitomycin plus tegafur in patients with resected stage III gastric cancer. J Clin Oncol 17 (1999) 3810-5.

[13] R.C. Coombes, P.S. Schein, C.E. Chilvers, J. Wils, G. Beretta, J.M. Bliss, A. Rutten, D. Amadori, H. Cortes-Funes, and A. Villar-Grimalt, A randomized trial comparing adjuvant fluorouracil, doxorubicin, and mitomycin with no treatment in operable gastric cancer. International Collaborative Cancer Group, Journal of clinical oncology : official journal of the American Society of Clinical Oncology, 1990, pp. 1362-9.

[14] D. Cunningham, W.H. Allum, S.P. Stenning, J.N. Thompson, C.J. Van de Velde, M. Nicolson, J.H. Scarffe, F.J. Lofts, S.J. Falk, T.J. Iveson, D.B. Smith, R.E. Langley, M. Verma, S. Weeden, Y.J. Chua, and M.T. Participants, Perioperative chemotherapy versus surgery alone for resectable gastroesophageal cancer. N Engl J Med 355 (2006) 11-20.

[15] D. Cunningham, S.P. Stenning, E.C. Smyth, A.F. Okines, W.H. Allum, S. Rowley, L. Stevenson, H.I. Grabsch, D. Alderson, T. Crosby, S.M. Griffin, W. Mansoor, F.Y. Coxon, S.J. Falk, S. Darby, K.A. Sumpter, J.M. Blazeby, and R.E. Langley, Peri-operative chemotherapy with or without bevacizumab in operable oesophagogastric adenocarcinoma (UK Medical Research Council ST03): primary analysis results of a multicentre, open-label, randomised phase 2-3 trial. The Lancet Oncology 18 (2017) 357-370.

[16] F. De Vita, F. Giuliani, M. Orditura, E. Maiello, G. Galizia, N. Di Martino, F. Montemurro, G. Carteni, L. Manzione, S. Romito, V. Gebbia, F. Ciardiello, G. Catalano, G. Colucci, and M. Gruppo Oncologico Italia, Adjuvant chemotherapy with epirubicin, leucovorin, 5-fluorouracil and etoposide regimen in resected gastric cancer patients: a randomized phase III trial by the Gruppo Oncologico Italia Meridionale (GOIM 9602 Study). Ann Oncol 18 (2007) 1354-8.

[17] F. Di Costanzo, S. Gasperoni, L. Manzione, G. Bisagni, R. Labianca, S. Bravi, E. Cortesi, P. Carlini, R. Bracci, S. Tomao, L. Messerini, A. Arcangeli, V. Torri, D. Bilancia, I. Floriani, M. Tonato, R. Italian Oncology Group for Cancer, A. Dinota, G. Strafiuso, E. Corgna, S. Porrozzi, C. Boni, E. Rondini, A. Giunta, B. Monzio Compagnoni, F. Biagioni, M. Cesari, G. Fornarini, F. Nelli, M. Carboni, F. Cognetti, M.R. Enzo, A. Piga, A. Romiti, A. Olivetti, L. Masoni, M. De Stefanis, A. Dalla Mola, S. Camera, F. Recchia, S. De Filippis, L. Scipioni, S. Zironi, G. Luppi, M. Italia, S. Banducci, A. Pisani Leretti, B. Massidda, M.T. Ionta, A. Nicolosi, R. Canaletti, B. Biscottini, F. Grigniani, F. Di Costanzo, R. Rovei, E. Croce, R. Carroccio, G. Gilli, C. Cavalli, A. Olgiati, U. Pandolfi, R. Rossetti, G. Natalini, P. Foa, S. Oldani, L. Bruno, S. Cascinu, G. Catalano, V. Catalano, F. Lungarotti, A. Farris, M.G. Sarobba, M. Trignano, A. Muscogiuri, F. Francavilla, F. Figoli, M. Leoni, G. Papiani, G. Orselli, M. Antimi, V. Bellini, A. Cabassi, A. Contu, A. Pazzola, M. Frignano, E. Lastraioli, M. Saggese, D. Bianchini, L. Antonuzzo, M. Mela, and R. Camisa, Adjuvant chemotherapy in completely resected gastric cancer: a randomized phase III trial conducted by GOIRC. J Natl Cancer Inst 100 (2008) 388-98.

[18] N. Fazio, R. Biffi, R. Maibach, S. Hayoz, S. Thierstein, P. Brauchli, J. Bernhard, R. Stupp, B. Andreoni, G. Renne, C. Crosta, R. Morant, A. Chiappa, F. Luca, M.G. Zampino, O. Huber, A. Goldhirsch, F. de Braud, A.D. Roth, U. Pace, S. Cenciarelli, S. Pozzi, E. Bertani, S. Mura, K. Lorizzo, G. Di Meglio, D. Ravizza, S. Boselli, M. Matter, M. Richter, S. Monfardini, C. Dittrich, M. Hafner, M. Clemens, and S. Crowe, Preoperative versus postoperative docetaxel-cisplatin- fluorouracil (TCF) chemotherapy in locally advanced resectable gastric carcinoma: 10-year follow-up of the SAKK 43/99 phase III trial. Ann Oncol 27 (2016) 668-673.

[19] W.M. Feng, C.W. Tang, H.H. Guo, Y. Bao, and M.Y. Fei, Prolonged adjuvant capecitabine chemotherapy improved survival of stage IIIA gastric cancer after D2 gastrectomy. Biomed Pharmacother 72 (2015) 140-3.

[20] C.S. Fuchs, D. Niedzwiecki, H.J. Mamon, J.E. Tepper, X. Ye, R.S. Swanson, P.C. Enzinger, D.G. Haller, T. Dragovich, S.R. Alberts, G.A. Bjarnason, C.G. Willett, L.L. Gunderson, R.M. Goldberg, A.P. Venook, D. Ilson, E. O'Reilly, K. Ciombor, D.J. Berg, J. Meyerhardt, and R.J. Mayer, Adjuvant Chemoradiotherapy With Epirubicin, Cisplatin, and Fluorouracil Compared With Adjuvant Chemoradiotherapy With Fluorouracil and Leucovorin After Curative Resection of Gastric Cancer: Results From CALGB 80101 (Alliance). J Clin Oncol 35 (2017) 3671-3677.

[21] H.H. Hartgrink, C.J. van de Velde, H. Putter, I. Songun, M.E. Tesselaar, E.K. Kranenbarg, J.E. de Vries, J.A. Wils, J. van der Bijl, J.H. van Krieken, and G. Cooperating Investigators of The Dutch Gastric Cancer, Neo-adjuvant chemotherapy for operable gastric cancer: long term results of the Dutch randomised FAMTX trial. Eur J Surg Oncol 30 (2004) 643-9.

[22] M. Imano, T. Itoh, T. Satou, Y. Sogo, H. Hirai, H. Kato, A. Yasuda, Y.F. Peng, M. Shinkai, T. Yasuda, H. Imamoto, K. Okuno, H. Shiozaki, and H. Ohyanagi, Prospective randomized trial of short-term neoadjuvant chemotherapy for advanced gastric cancer. Eur J Surg Oncol 36 (2010) 963-8.

[23] Y.K. Kang, H.M. Chang, J.H. Yook, M.H. Ryu, I. Park, Y.J. Min, D.Y. Zang, G.Y. Kim, D.H. Yang, S.J. Jang, Y.S. Park, J.L. Lee, T.W. Kim, S.T. Oh, B.K. Park, H.Y. Jung, and B.S. Kim, Adjuvant chemotherapy for gastric cancer: a randomised phase 3 trial of mitomycin-C plus either short-term doxifluridine or long-term doxifluridine plus cisplatin after curative D2 gastrectomy (AMC0201). Br J Cancer 108 (2013) 1245-51.

[24] T.H. Kim, S.R. Park, K.W. Ryu, Y.W. Kim, J.M. Bae, J.H. Lee, I.J. Choi, Y.J. Kim, and D.Y. Kim, Phase 3 trial of postoperative chemotherapy alone versus chemoradiation therapy in stage III-IV gastric cancer treated with R0 gastrectomy and D2 lymph node dissection. Int J Radiat Oncol Biol Phys 84 (2012) e585-92.

[25] J.E. Krook, M.J. O'Connell, H.S. Wieand, R.W. Beart, Jr., J.E. Leigh, J.W. Kugler, J.F. Foley, D.M. Pfeifle, and D.I. Twito, A prospective, randomized evaluation of intensive-course 5-fluorouracil plus doxorubicin as surgical adjuvant chemotherapy for resected gastric cancer. Cancer 67 (1991) 2454-8.

[26] J. Kulig, P. Kolodziejczyk, M. Sierzega, L. Bobrzynski, J. Jedrys, T. Popiela, J. Dadan, M. Drews, A. Jeziorski, M. Krawczyk, T. Starzynska, and G. Wallner, Adjuvant chemotherapy with etoposide, adriamycin and cisplatin compared with surgery alone in the treatment of gastric cancer: a phase III randomized, multicenter, clinical trial. Oncology 78 (2010) 54-61.

[27] H.C. Kwon, M.C. Kim, K.H. Kim, J.S. Jang, S.Y. Oh, S.H. Kim, K.A. Kwon, S. Lee, H.S. Lee, and H.J. Kim, Adjuvant chemoradiation versus chemotherapy in completely resected advanced gastric cancer with D2 nodal dissection. Asia Pac J Clin Oncol 6 (2010) 278-85.

[28] J.J. Lee, S.Y. Kim, I. Shin, K.S. Cho, H.Z. Joo, and C. Yoon, Randomized Phase III Trial of Cisplatin, Epirubicin, Leucovorin, 5-Fluorouracil (PELF) Combination versus 5-fluorouracil Alone as Adjuvant Chemotherapy in Curative Resected Stage III Gastric Cancer, Cancer Research and Treatment, 2004, pp. 140-5.

[29] C.K. Lee, M. Jung, H.S. Kim, I. Jung, D.B. Shin, S.Y. Kang, D.Y. Zang, K.H. Kim, M.H. Lee, B.S. Kim, K.H. Lee, J.H. Cheong, W.J. Hyung, S.H. Noh, H.C. Chung, and S.Y. Rha, S-1 Based Doublet as an Adjuvant Chemotherapy for Curatively Resected Stage III Gastric Cancer: Results from the Randomized Phase III POST Trial. Cancer Res 51 (2019) 1-11.

[30] T. Leong, B.M. Smithers, K. Haustermans, M. Michael, V. Gebski, D. Miller, J. Zalcberg, A. Boussioutas, M. Findlay, R.L. O'Connell, J. Verghis, D. Willis, T. Kron, M. Crain, W.K. Murray, F. Lordick, C. Swallow, G. Darling, J. Simes, and R. Wong, TOPGEAR: A Randomized, Phase III Trial of Perioperative ECF Chemotherapy with or Without Preoperative Chemoradiation for Resectable Gastric Cancer: Interim Results from an International, Intergroup Trial of the AGITG, TROG, EORTC and CCTG. Ann Surg Oncol 24 (2017) 2252-2258.

[31] M. Lise, D. Nitti, A. Marchet, T. Sahmoud, M. Buyse, N. Duez, M. Fiorentino, J.G. Dos Santos, R. Labianca, P. Rougier, and et al., Final results of a phase III clinical trial of adjuvant chemotherapy with the modified fluorouracil, doxorubicin, and mitomycin regimen in resectable gastric cancer. J Clin Oncol 13 (1995) 2757-63.

[32] T. Nakajima, A. Nashimoto, M. Kitamura, T. Kito, T. Iwanaga, K. Okabayashi, and M. Goto, Adjuvant mitomycin and fluorouracil followed by oral uracil plus tegafur in serosa-negative gastric cancer: a randomised trial. Gastric Cancer Surgical Study Group. Lancet 354 (1999) 273-7.

[33] T. Nakajima, T. Kinoshita, A. Nashimoto, M. Sairenji, T. Yamaguchi, J. Sakamoto, T. Fujiya, T. Inada, M. Sasako, Y. Ohashi, and G. National Surgical Adjuvant Study of Gastric Cancer, Randomized controlled trial of adjuvant uracil-tegafur versus surgery alone for serosa-negative, locally advanced gastric cancer. Br J Surg 94 (2007) 1468-76.

[34] B. Neri, G. Cini, F. Andreoli, B. Boffi, D. Francesconi, R. Mazzanti, F. Medi, A. Mercatelli, S. Romano, L. Siliani, R. Tarquini, and R. Moretti, Randomized trial of adjuvant chemotherapy versus control after curative resection for gastric cancer: 5-year follow-up. Br J Cancer 84 (2001) 878-80.

[35] D. Nitti, J. Wils, J.G. Dos Santos, G. Fountzilas, P.F. Conte, C. Sava, A. Tres, R.C. Coombes, D. Crivellari, A. Marchet, E. Sanchez, J.M. Bliss, J. Homewood, M.L. Couvreur, E. Hall, B. Baron, E. Woods, M. Emson, E. Van Cutsem, M. Lise, E.G. Group, and Iccg, Randomized phase III trials of adjuvant FAMTX or FEMTX compared with surgery alone in resected gastric cancer. A combined analysis of the EORTC GI Group and the ICCG. Ann Oncol 17 (2006) 262-9.

[36] S.H. Noh, S.R. Park, H.K. Yang, H.C. Chung, I.J. Chung, S.W. Kim, H.H. Kim, J.H. Choi, H.K. Kim, W. Yu, J.I. Lee, D.B. Shin, J. Ji, J.S. Chen, Y. Lim, S. Ha, Y.J. Bang, and C.t. investigators, Adjuvant capecitabine plus oxaliplatin for gastric cancer after D2 gastrectomy (CLASSIC): 5-year follow-up of an open-label, randomised phase 3 trial. Lancet Oncol 15 (2014) 1389-96.

[37] S.H. Park, T.S. Sohn, J. Lee, D.H. Lim, M.E. Hong, K.M. Kim, I. Sohn, S.H. Jung, M.G. Choi, J.H. Lee, J.M. Bae, S. Kim, S.T. Kim, J.O. Park, Y.S. Park, H.Y. Lim, and W.K. Kang, Phase III trial to compare adjuvant chemotherapy with capecitabine and cisplatin versus concurrent chemoradiotherapy in gastric cancer: Final report of the adjuvant chemoradiotherapy in stomach tumors trial, including survival and subset analyses, J Clin Oncol, 2015, pp. 3130-6.

[38] M. Sasako, S. Sakuramoto, H. Katai, T. Kinoshita, H. Furukawa, T. Yamaguchi, A. Nashimoto, M. Fujii, T. Nakajima, and Y. Ohashi, Five-year outcomes of a randomized phase III trial comparing adjuvant chemotherapy with S-1 versus surgery alone in stage II or III gastric cancer. J Clin Oncol 29 (2011) 4387-93.

[39] C. Schuhmacher, S. Gretschel, F. Lordick, P. Reichardt, W. Hohenberger, C.F. Eisenberger, C. Haag, M.E. Mauer, B. Hasan, J. Welch, K. Ott, A. Hoelscher, P.M. Schneider, W. Bechstein, H. Wilke, M.P. Lutz, B. Nordlinger, E. Van Cutsem, J.R. Siewert, and P.M. Schlag, Neoadjuvant chemotherapy compared with surgery alone for locally advanced cancer of the stomach and cardia: European Organisation for Research and Treatment of Cancer randomized trial 40954. J Clin Oncol 28 (2010) 5210-8.

[40] G.K. Schwartz, K. Winter, B.D. Minsky, C. Crane, P.J. Thomson, P. Anne, H. Gross, C. Willett, and D. Kelsen, Randomized phase II trial evaluating two paclitaxel and cisplatin-containing chemoradiation regimens as adjuvant therapy in resected gastric cancer (RTOG-0114), Journal of clinical oncology : official journal of the American Society of Clinical Oncology, 2009, pp. 1956-62.

[41] S.R. Smalley, J.K. Benedetti, D.G. Haller, S.A. Hundahl, N.C. Estes, J.A. Ajani, L.L. Gunderson, B. Goldman, J.A. Martenson, J.M. Jessup, G.N. Stemmermann, C.D. Blanke, and J.S. Macdonald, Updated analysis of SWOG-directed intergroup study 0116: a phase III trial of adjuvant radiochemotherapy versus observation after curative gastric cancer resection. J Clin Oncol 30 (2012) 2327-33.

[42] A.A. Tentes, S.K. Markakidis, C. Karanikiotis, A. Fiska, I.K. Tentes, V.G. Manolopoulos, and T. Dimitriou, Intraarterial chemotherapy as an adjuvant treatment in locally advanced gastric cancer. Langenbecks Arch Surg 391 (2006) 124-9.

[43] M. Terashima, Y. Iwasaki, J. Mizusawa, H. Katayama, K. Nakamura, H. Katai, T. Yoshikawa, Y. Ito, M. Kaji, Y. Kimura, M. Hirao, M. Yamada, A. Kurita, M. Takagi, N. Boku, T. Sano, M. Sasako, and J.C.O.G. Stomach Cancer Study Group, Randomized phase III trial of gastrectomy with or without neoadjuvant S-1 plus cisplatin for type 4 or large type 3 gastric cancer, the short-term safety and surgical results: Japan Clinical Oncology Group Study (JCOG0501). Gastric Cancer 02 (2019) 02.

[44] N. Tsavaris, K. Tentas, P. Kosmidis, N. Mylonakis, N. Sakelaropoulos, C. Kosmas, B. Lisaios, A. Soumilas, D. Mandrekas, A. Tsetis, and C. Klonaris, A randomized trial comparing adjuvant fluorouracil, epirubicin, and mitomycin with no treatment in operable gastric cancer. Chemotherapy 42 (1996) 220-6.

[45] A. Tsuburaya, K. Yoshida, M. Kobayashi, S. Yoshino, M. Takahashi, N. Takiguchi, K. Tanabe, N. Takahashi, H. Imamura, N. Tatsumoto, A. Hara, K. Nishikawa, R. Fukushima, I. Nozaki, H. Kojima, Y. Miyashita, K. Oba, M. Buyse, S. Morita, and J. Sakamoto, Sequential paclitaxel followed by tegafur and uracil (UFT) or S-1 versus UFT or S-1 monotherapy as adjuvant chemotherapy for T4a/b gastric cancer (SAMIT): a phase 3 factorial randomised controlled trial. Lancet Oncol 15 (2014) 886-93.

[46] T. Tsujinaka, H. Shiozaki, M. Inoue, H. Furukawa, M. Hiratsuka, N. Kikkawa, M. Takami, T. Suzuki, and M. Monden, Evaluation of effectiveness of chemotherapy in patients with gastric cancer after curative resection. Int J Clin Oncol 5 (2000) 372-379.

[47] K. Xue, X. Ying, Z. Bu, A. Wu, Z. Li, L. Tang, L. Zhang, Y. Zhang, Z. Li, and J. Ji, Oxaliplatin plus S-1 or capecitabine as neoadjuvant or adjuvant chemotherapy for locally advanced gastric cancer with D2 lymphadenectomy: 5-year follow-up results of a phase II<b>-</b>III randomized trial. Chinese Journal of Cancer Research 30 (2018) 516-525.

[48] M. Ychou, V. Boige, J.P. Pignon, T. Conroy, O. Bouche, G. Lebreton, M. Ducourtieux, L. Bedenne, J.M. Fabre, B. Saint-Aubert, J. Geneve, P. Lasser, and P. Rougier, Perioperative chemotherapy compared with surgery alone for resectable gastroesophageal adenocarcinoma: an FNCLCC and FFCD multicenter phase III trial. J Clin Oncol 29 (2011) 1715-21.

[49] T. Yoshikawa, S. Morita, K. Tanabe, K. Nishikawa, Y. Ito, T. Matsui, K. Fujitani, Y. Kimura, J. Fujita, T. Aoyama, T. Hayashi, H. Cho, A. Tsuburaya, Y. Miyashita, and J. Sakamoto, Survival results of a randomised two-by-two factorial phase II trial comparing neoadjuvant chemotherapy with two and four courses of S-1 plus cisplatin (SC) and paclitaxel plus cisplatin (PC) followed by D2 gastrectomy for resectable advanced gastric cancer. Eur J Cancer 62 (2016) 103-11.

[50] T. Yoshikawa, M. Terashima, J. Mizusawa, S. Nunobe, Y. Nishida, T. Yamada, M. Kaji, N. Fukushima, S. Hato, Y. Choda, H. Yabusaki, K. Yoshida, S. Ito, A. Takeno, T. Yasuda, Y. Kawachi, H. Katayama, H. Fukuda, N. Boku, T. Sano, and M. Sasako, Four courses versus eight courses of adjuvant S-1 for patients with stage II gastric cancer (JCOG1104 [OPAS-1]): an open-label, phase 3, non-inferiority, randomised trial. Lancet Gastroenterol Hepatol 4 (2019) 208-216.

[51] C. Yu, R. Yu, W. Zhu, Y. Song, and T. Li, Intensity-modulated radiotherapy combined with chemotherapy for the treatment of gastric cancer patients after standard D1/D2 surgery, Journal of cancer research and clinical oncology, 2012, pp. 255-9.

[52] X.L. Zhang, H.J. Shi, S.Z. Cui, Y.Q. Tang, and M.C. Ba, Prospective, randomized trial comparing 5-FU/LV with or without oxaliplatin as adjuvant treatment following curative resection of gastric adenocarcinoma.[Erratum appears in Eur J Surg Oncol. 2013 May;39(5):525]. Eur J Surg Oncol 37 (2011) 466-72.

[53] W.H. Zhao, S.F. Wang, W. Ding, J.M. Sheng, Z.M. Ma, L.S. Teng, M. Wang, F.S. Wu, and B. Luo, Apoptosis induced by preoperative oral 5'-DFUR administration in gastric adenocarcinoma and its mechanism of action, World J Gastroenterol, 2006, pp. 1356-61.

[54] Q. Zhao, Y. Li, J. Huang, L. Fan, B. Tan, Y. Tian, P. Yang, Z. Jiao, X. Zhao, Z. Zhang, D. Wang, and Y. Liu, Short-term curative effect of S-1 plus oxaliplatin as perioperative chemotherapy for locally advanced gastric cancer: a prospective comparison study. Pharmazie 72 (2017) 236-240.

[55] W.G. Zhu, D.F. Xua, J. Pu, C.D. Zong, T. Li, G.Z. Tao, F.Z. Ji, X.L. Zhou, J.H. Han, C.S. Wang, C.H. Yu, J.G. Yi, X.L. Su, and J.X. Ding, A randomized, controlled, multicenter study comparing intensity-modulated radiotherapy plus concurrent chemotherapy with chemotherapy alone in gastric cancer patients with D2 resection. Radiother Oncol 104 (2012) 361-6.
