# Supplementary material for: COMplot, A Graphical Presentation of Complication Profiles and Adverse Effects for the Curative Treatment of Gastric Cancer: A Systematic Review and Meta-Analysis
Source: Front Oncol. 2019 Jul 25;9:684. doi: 10.3389/fonc.2019.00684 (PMC6677173; doi:10.3389/fonc.2019.00684)
Supplement: Supplementary file 2 [file Data_Sheet_2.docx]

**Supplementary figures preoperative and postoperative related toxicity and surgery related morbidity/mortality during perioperative treatment**


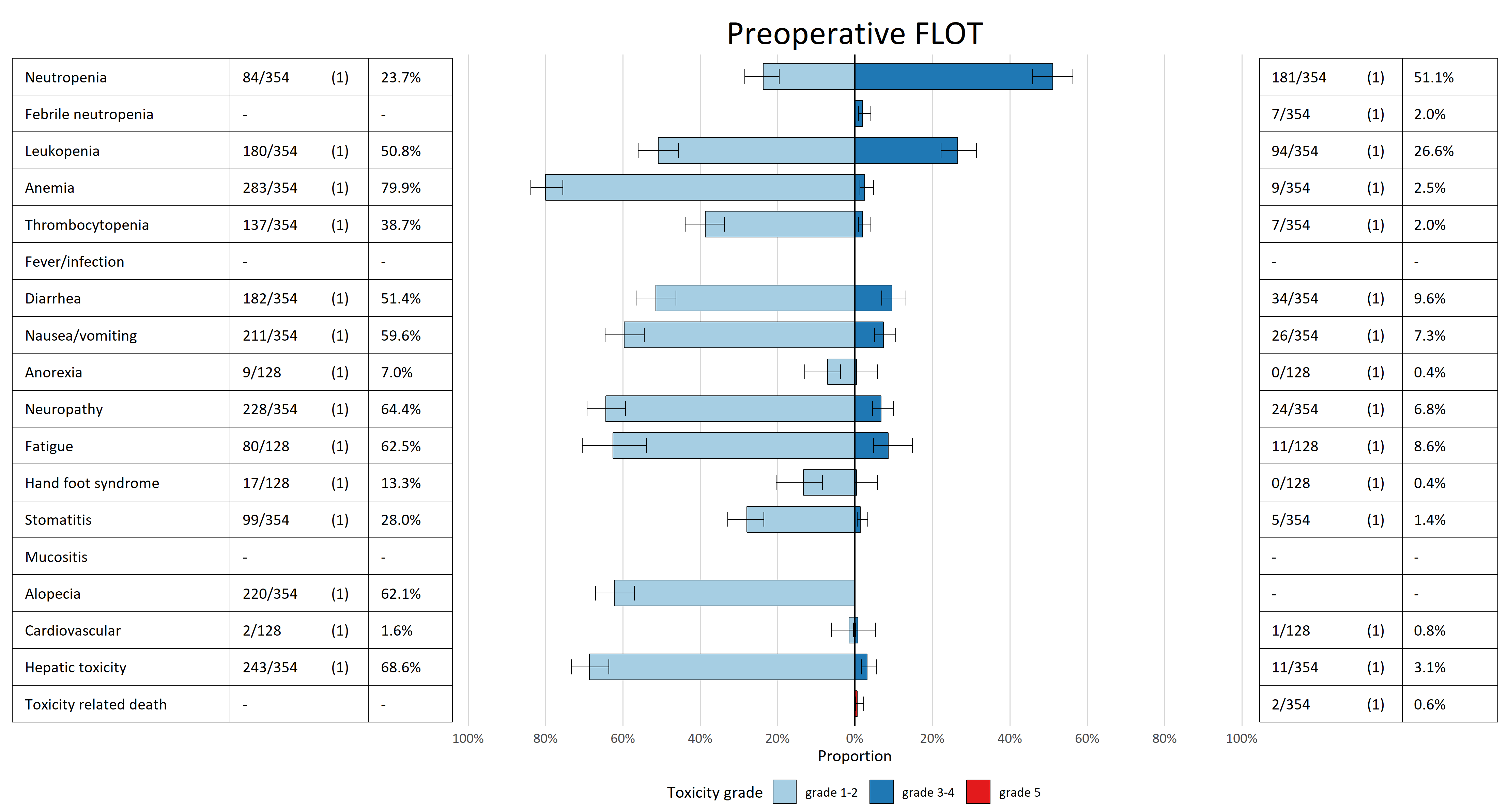


FLOT: 5-FU, leucovorin, oxaliplatin, taxane


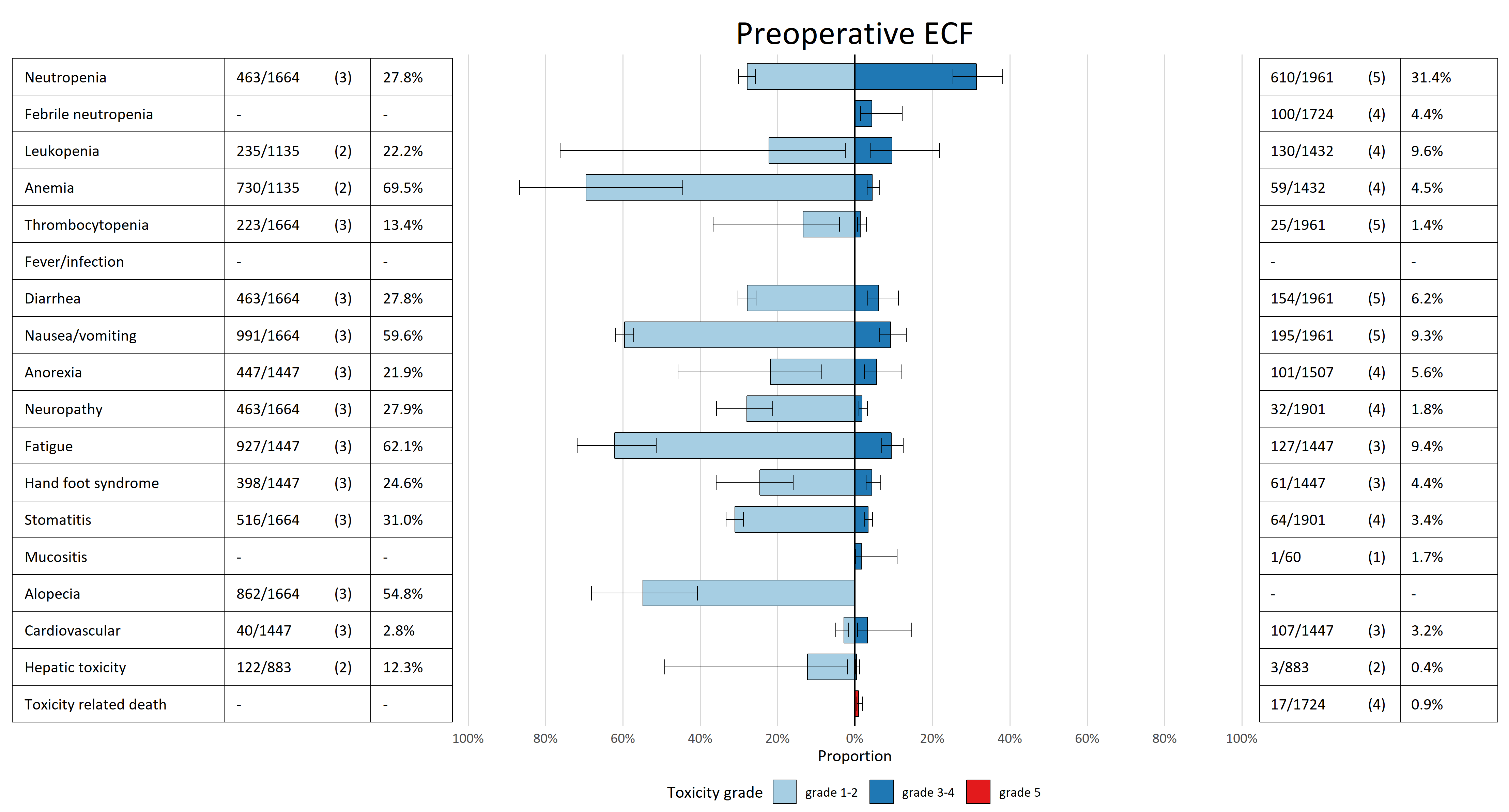


ECF: epirubicin, cisplatin, fluoropyrimidine


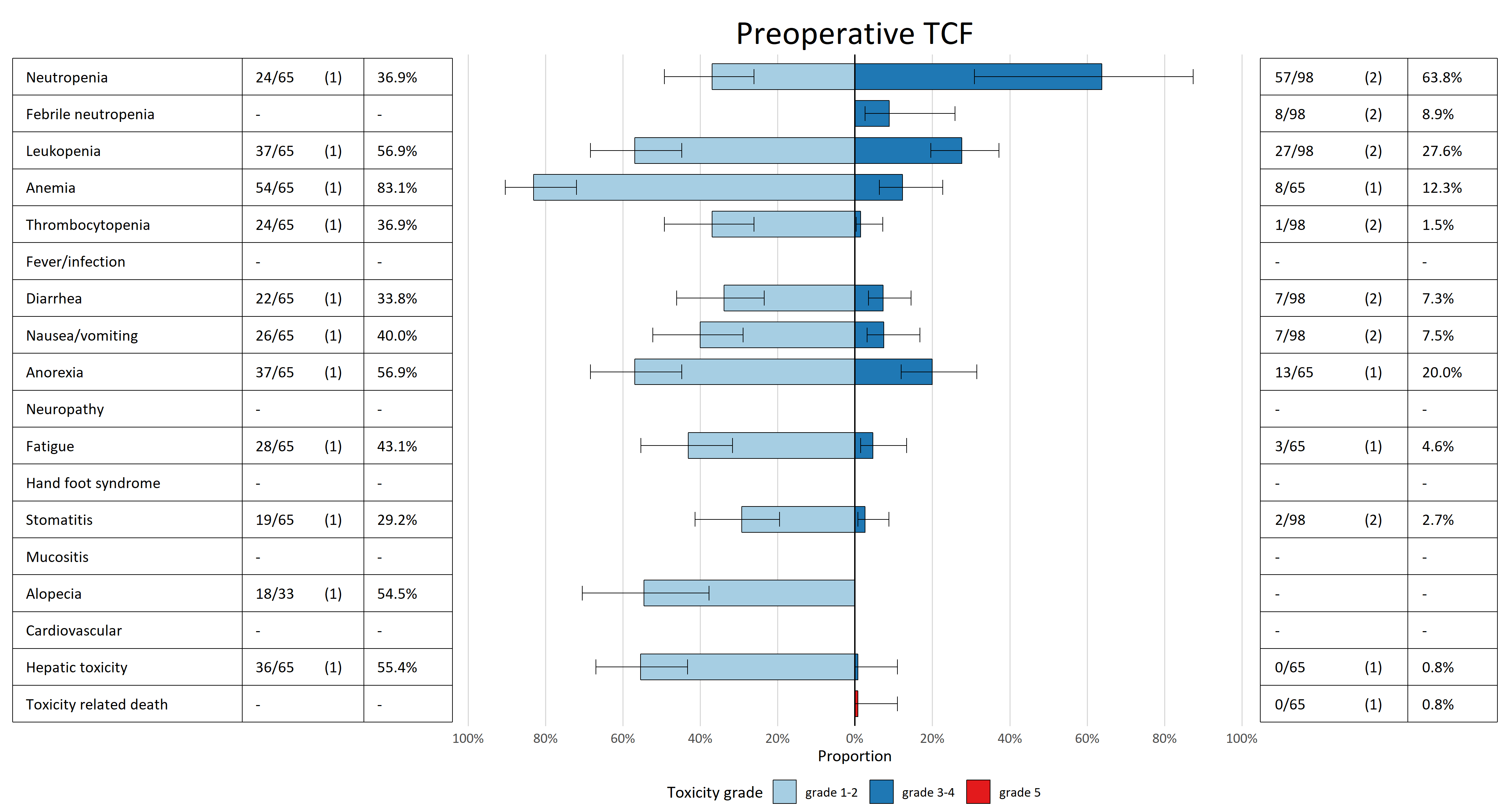


TCF: taxane, cisplatin, fluoropyrimidine


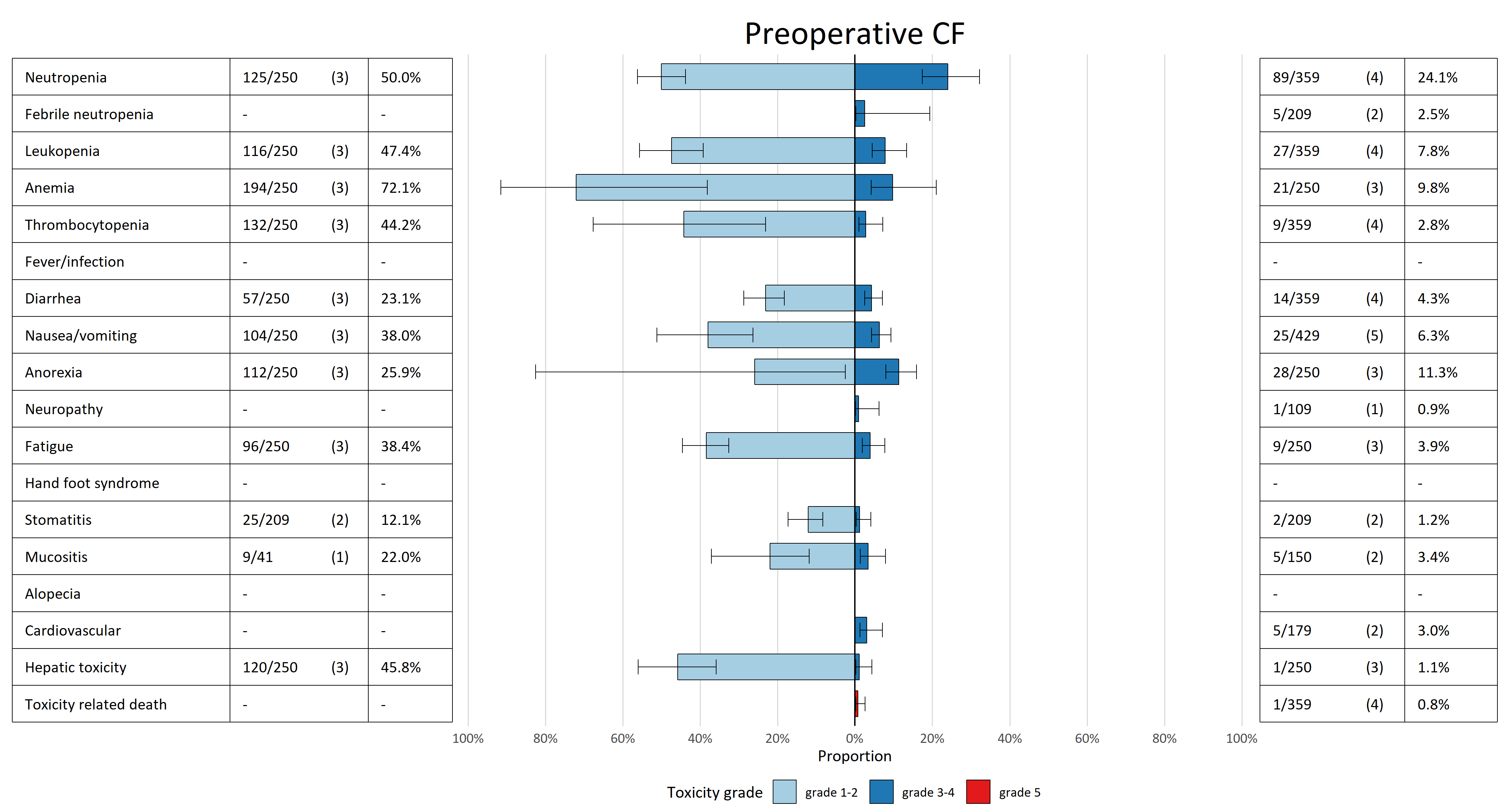


CF: cisplatin, fluoropyrimidine


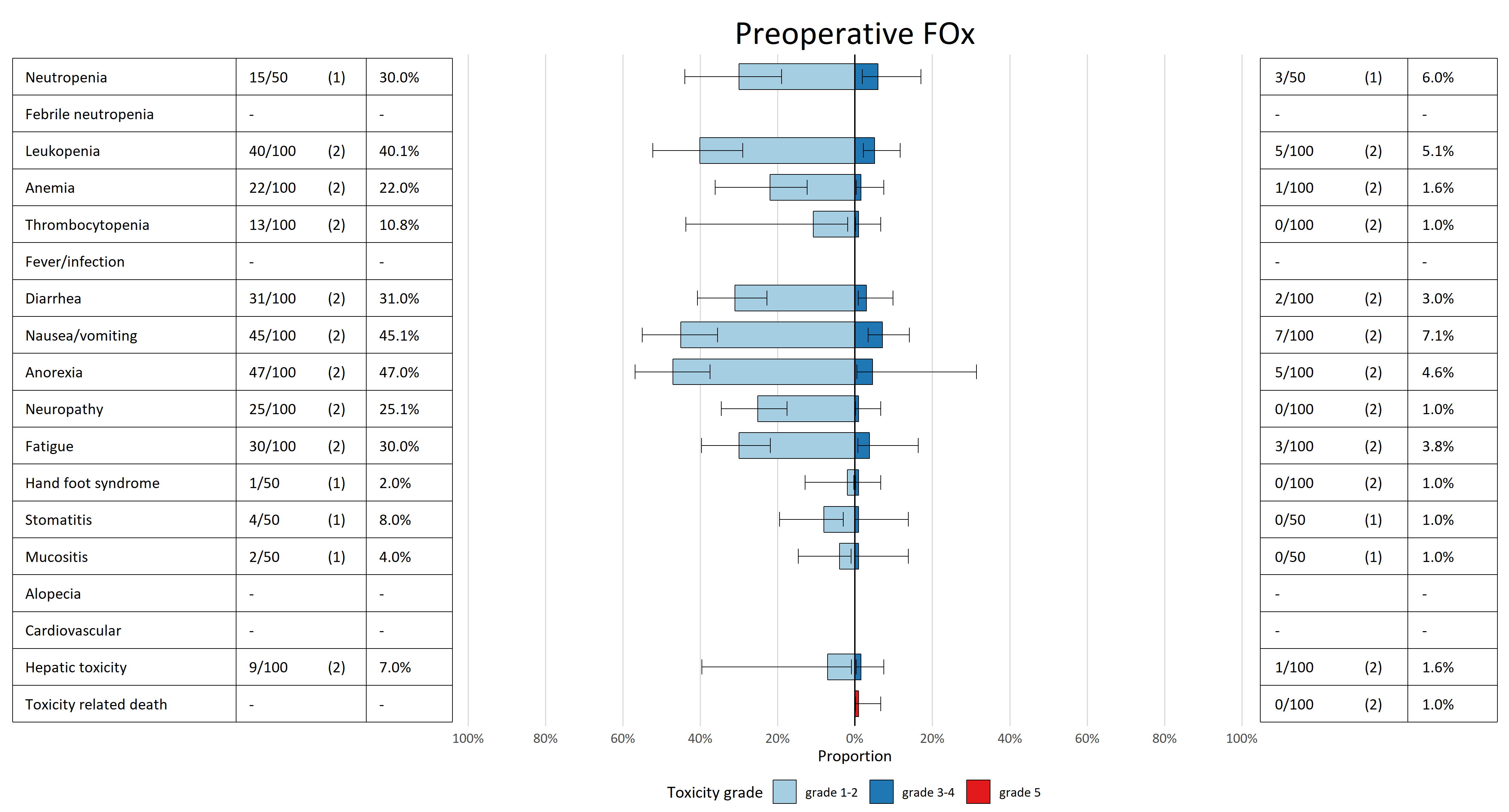


FOx: fluoropyrimidine, oxaliplatin


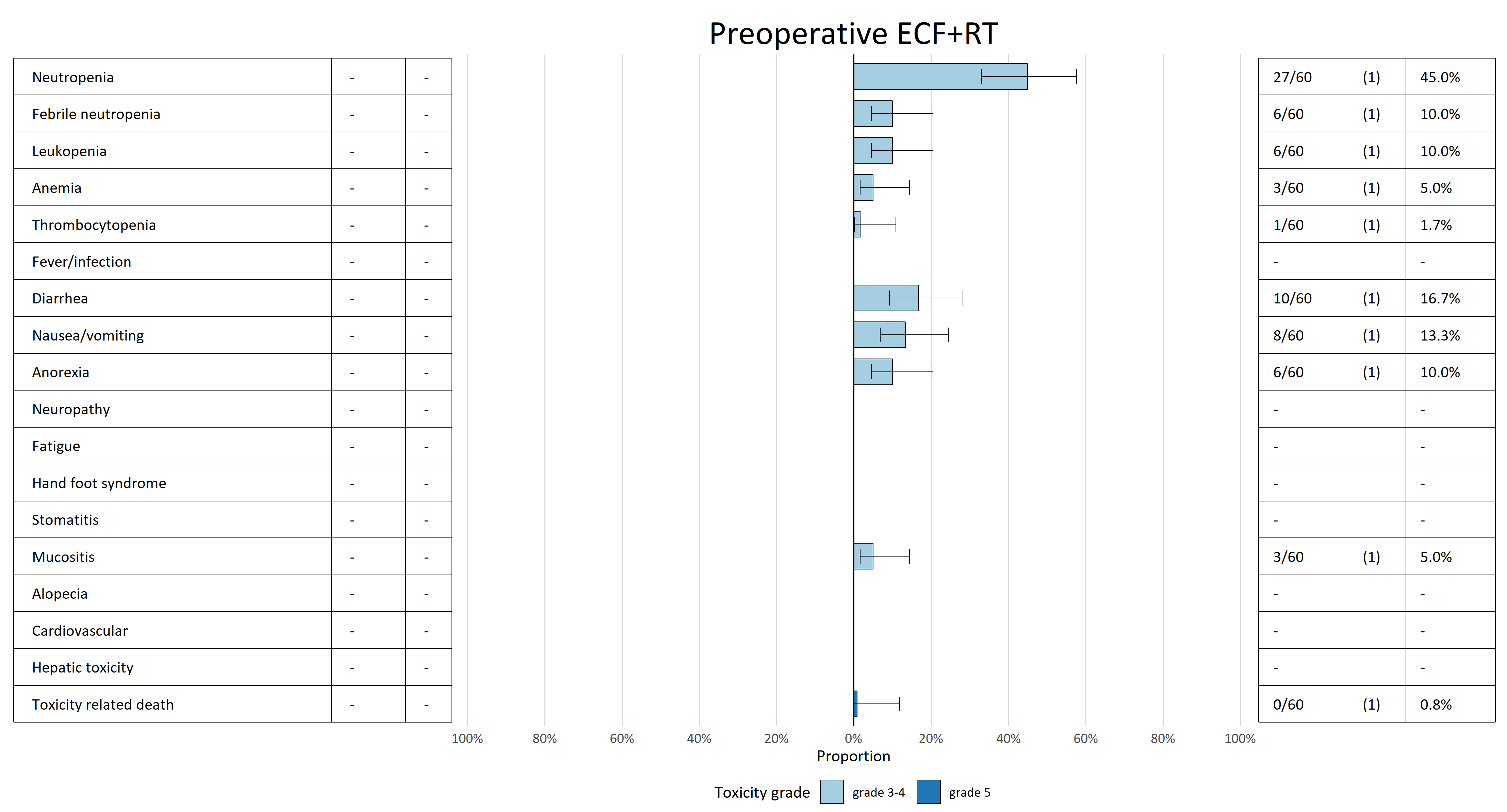


ECF+RT: epirubicin, cisplatin, fluoropyrimidine, radiotherapy


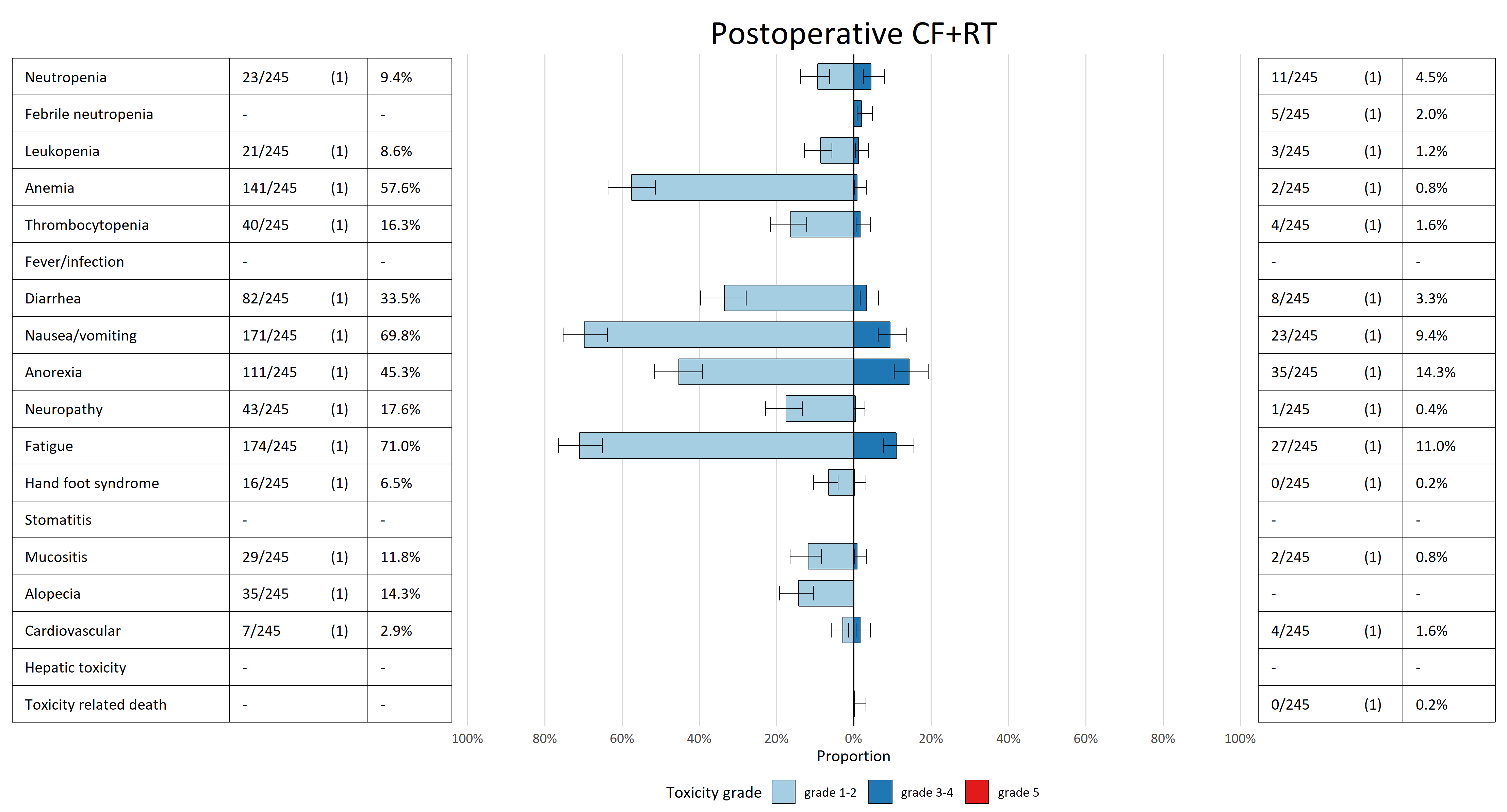


CF+RT: cisplatin, fluoropyrimidine, radiotherapy


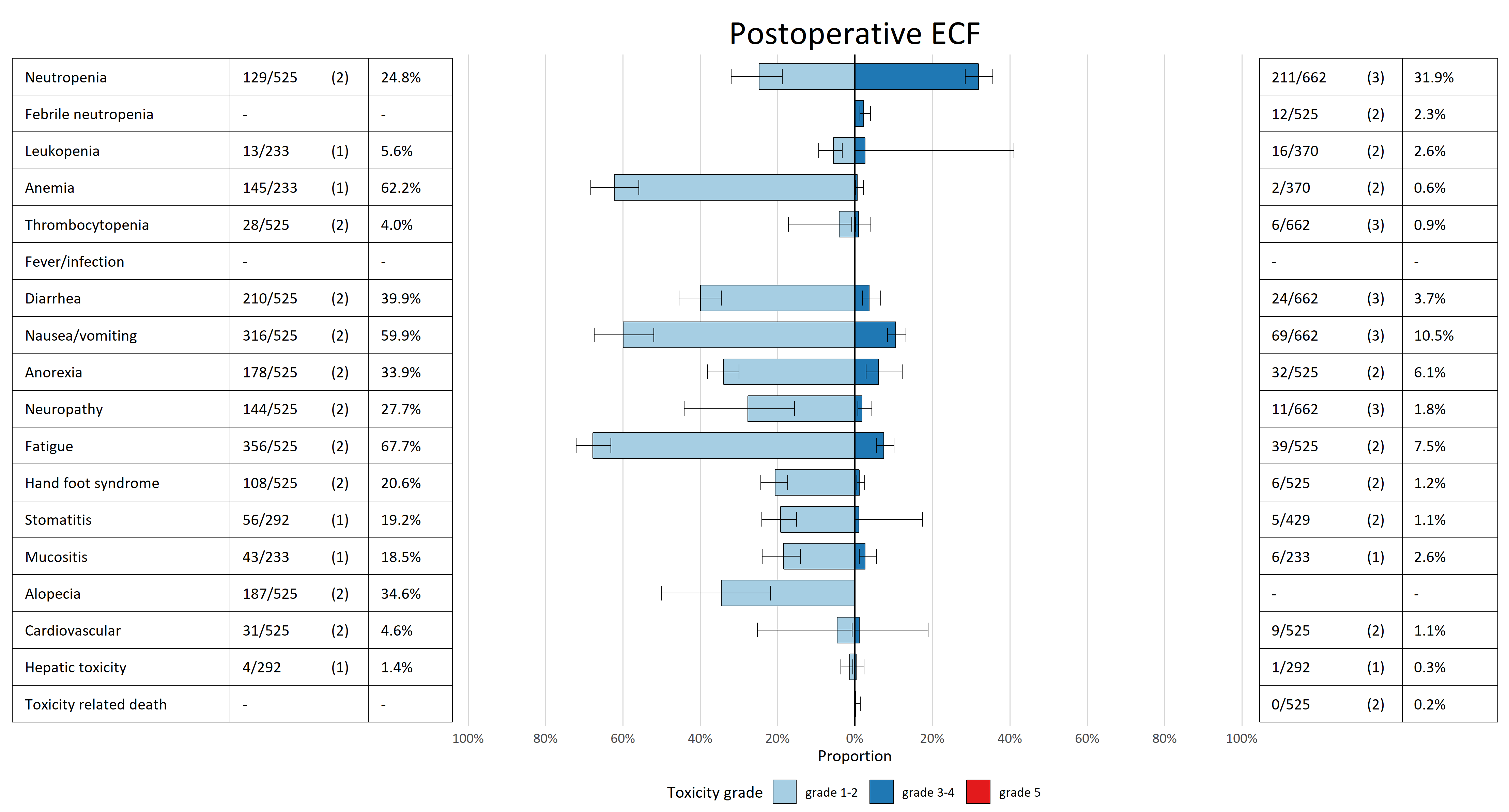


ECF: epirubicin, cisplatin, fluoropyrimidine


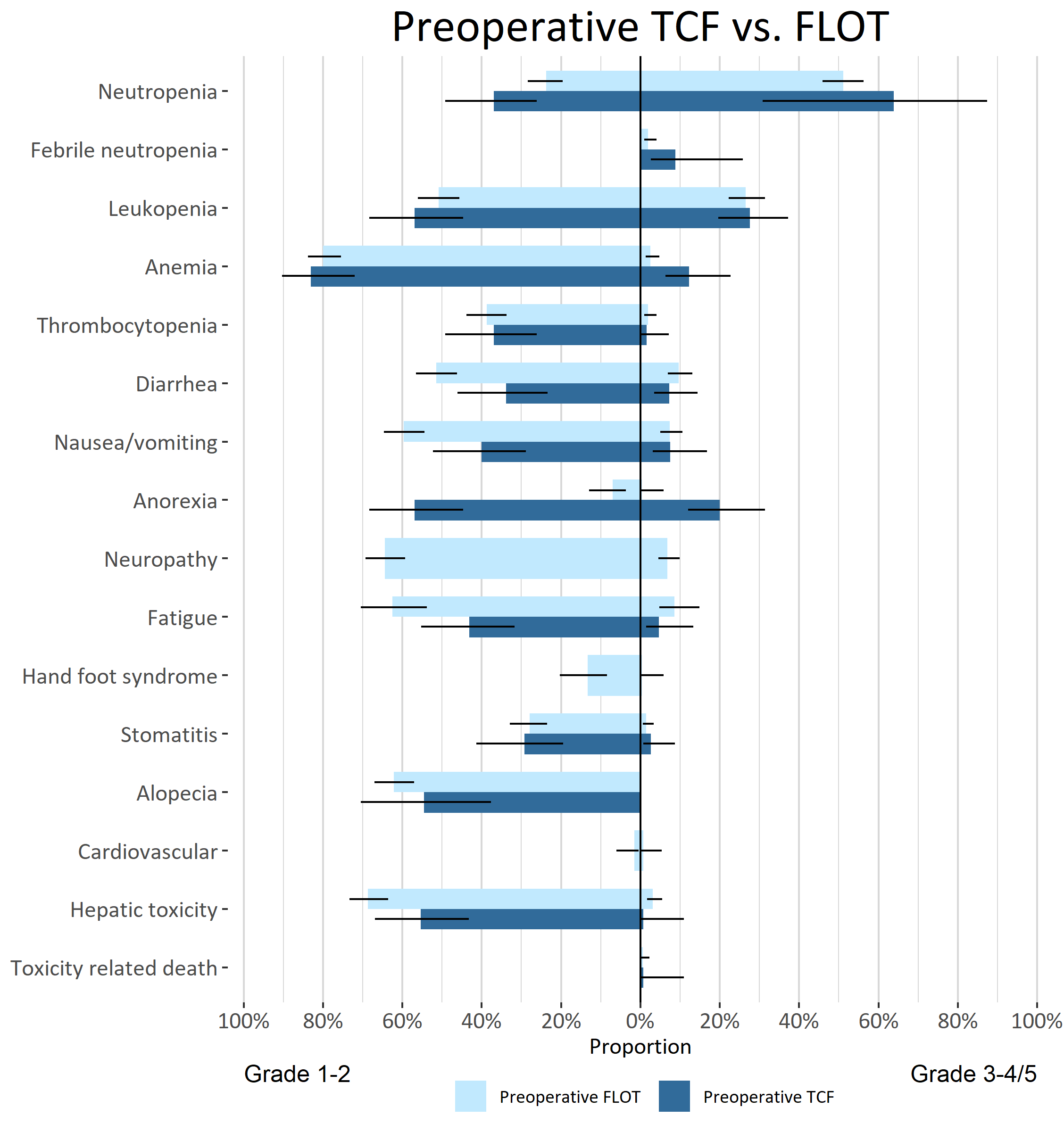


TCF: taxane, cisplatin, fluoropyrimidine; FLOT: 5-FU, leucovorin, oxaliplatin, taxane


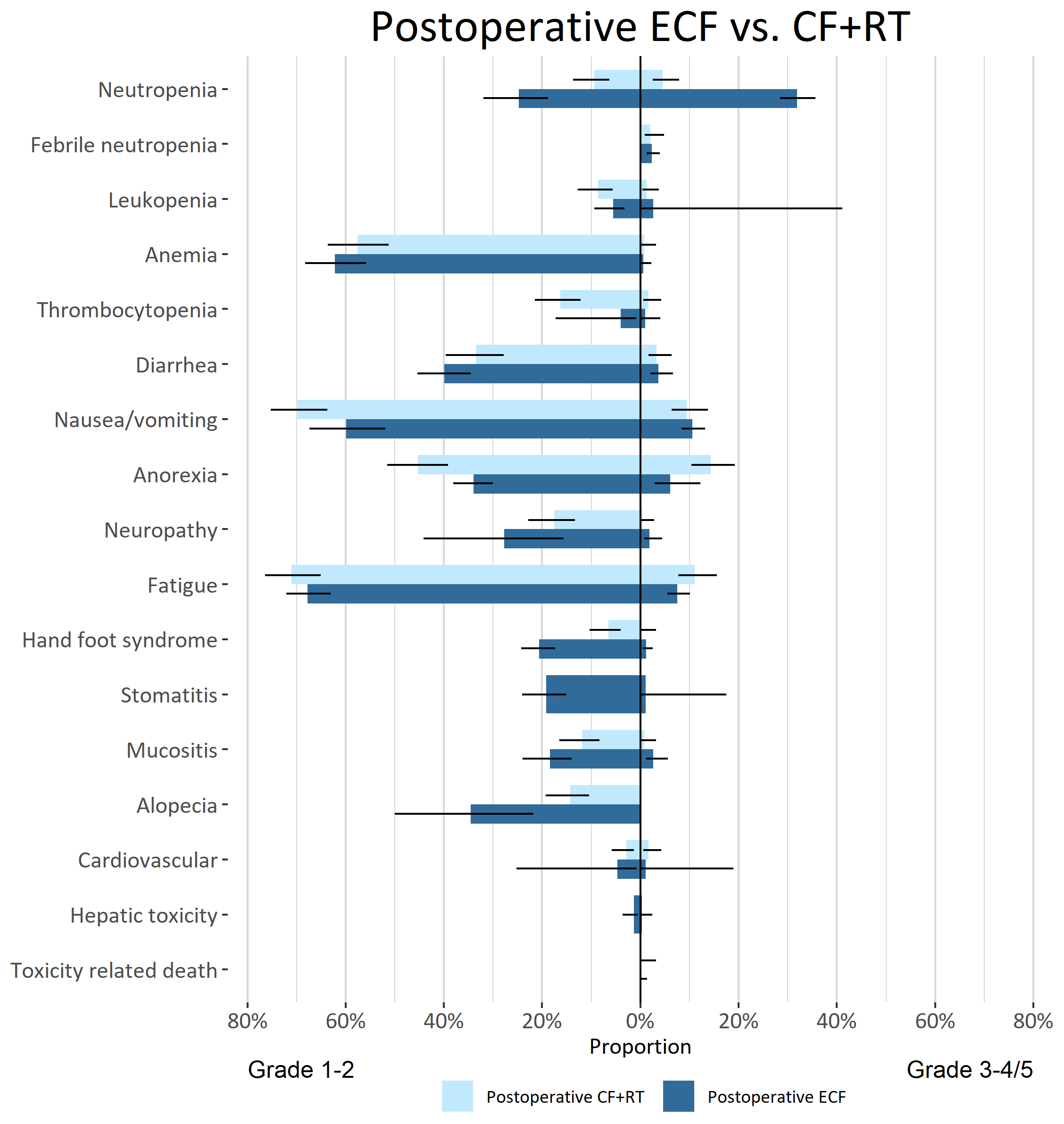


ECF: epirubicin, cisplatin, fluoropyrimidine; CF+RT: cisplatin, fluoropyrimidine, radiotherapy


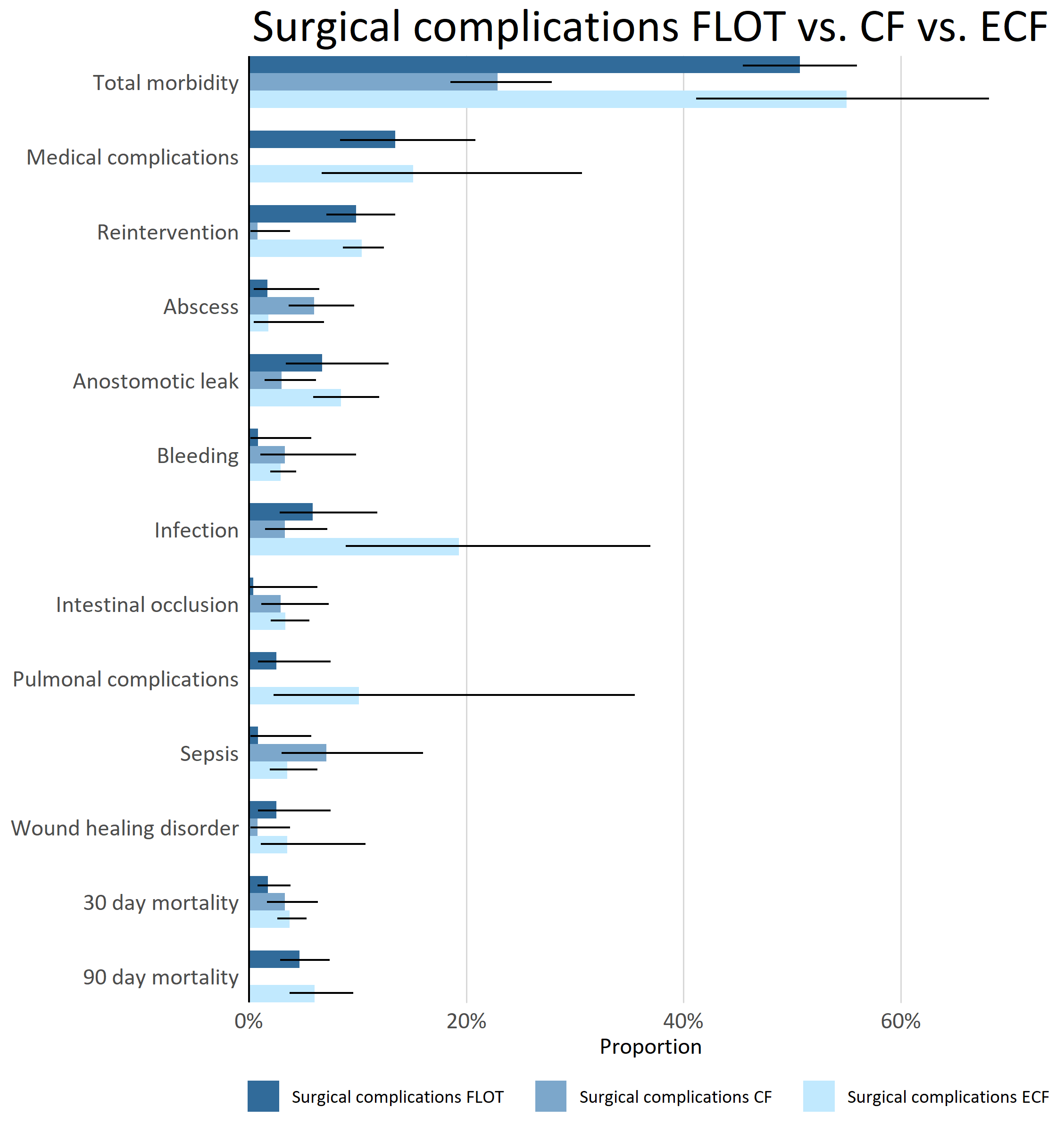


FLOT: 5-FU, leucovorin, oxaliplatin, taxane; CF: cisplatin, fluoropyrimidine; ECF: epirubicin, cisplatin, fluoropyrimidine

**Supplementary Figures adjuvant regimens**


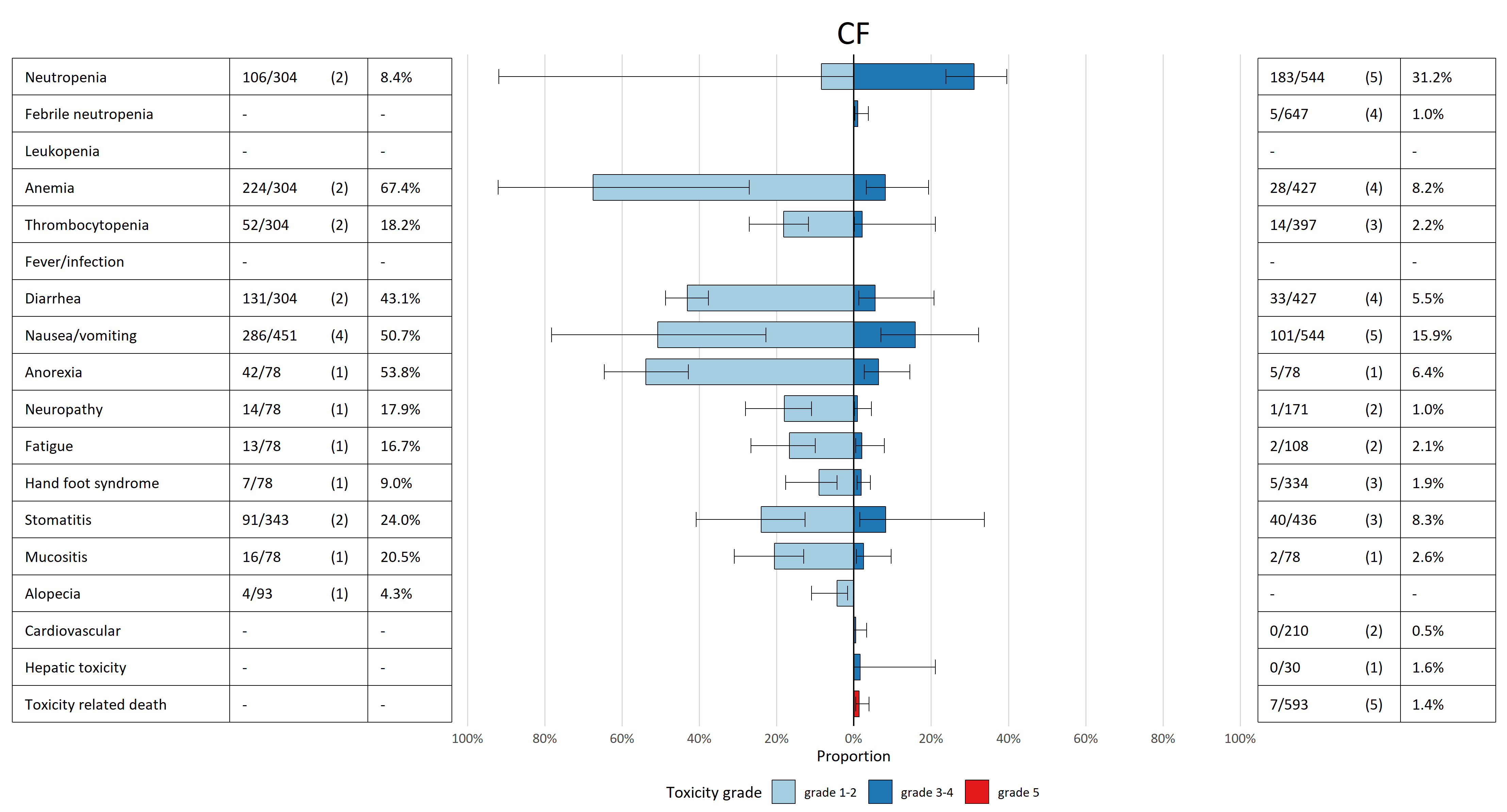


CF: cisplatin, fluoropyrimidine


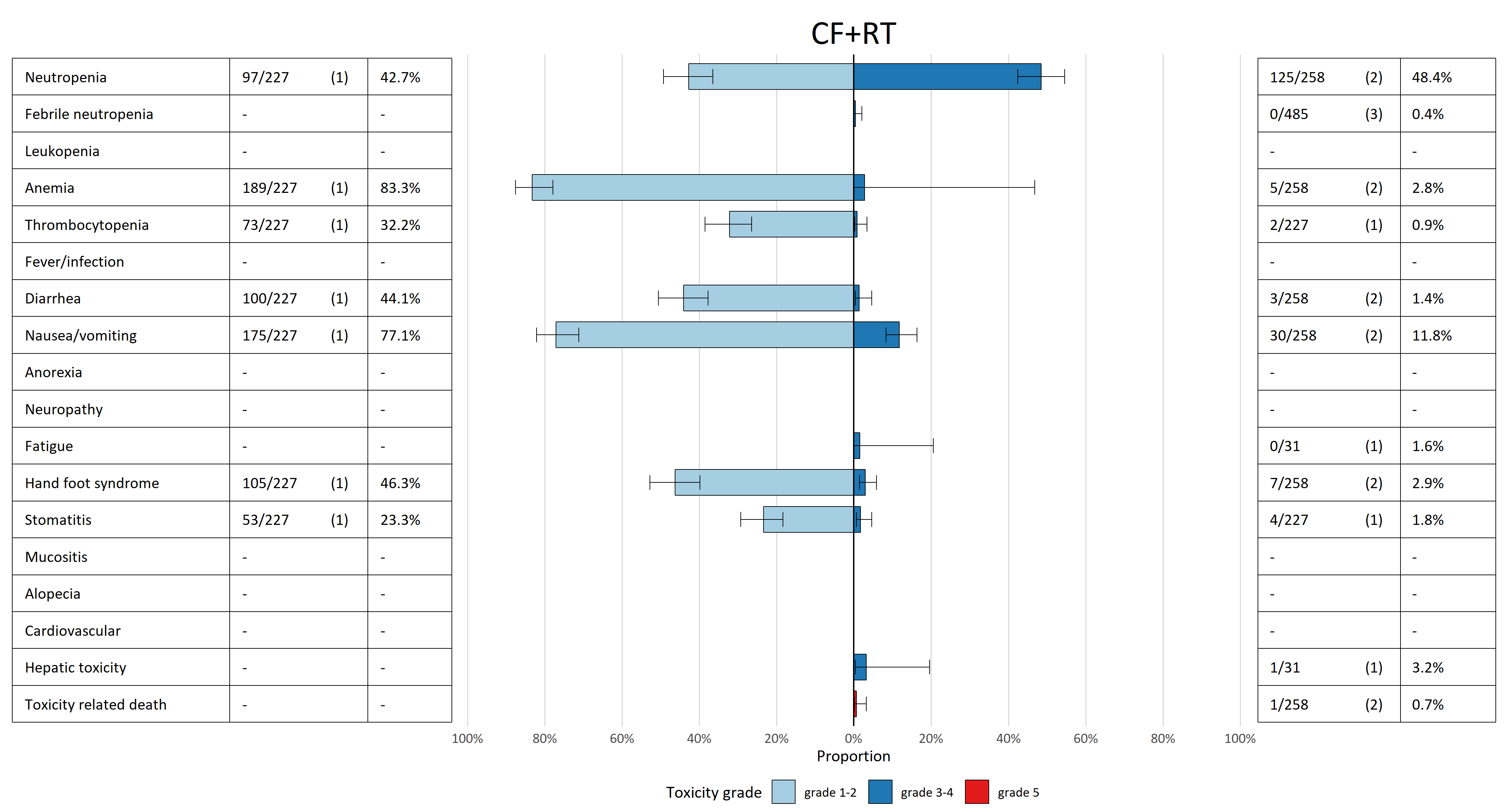


CF+RT: cisplatin, fluoropyrimidine, radiotherapy


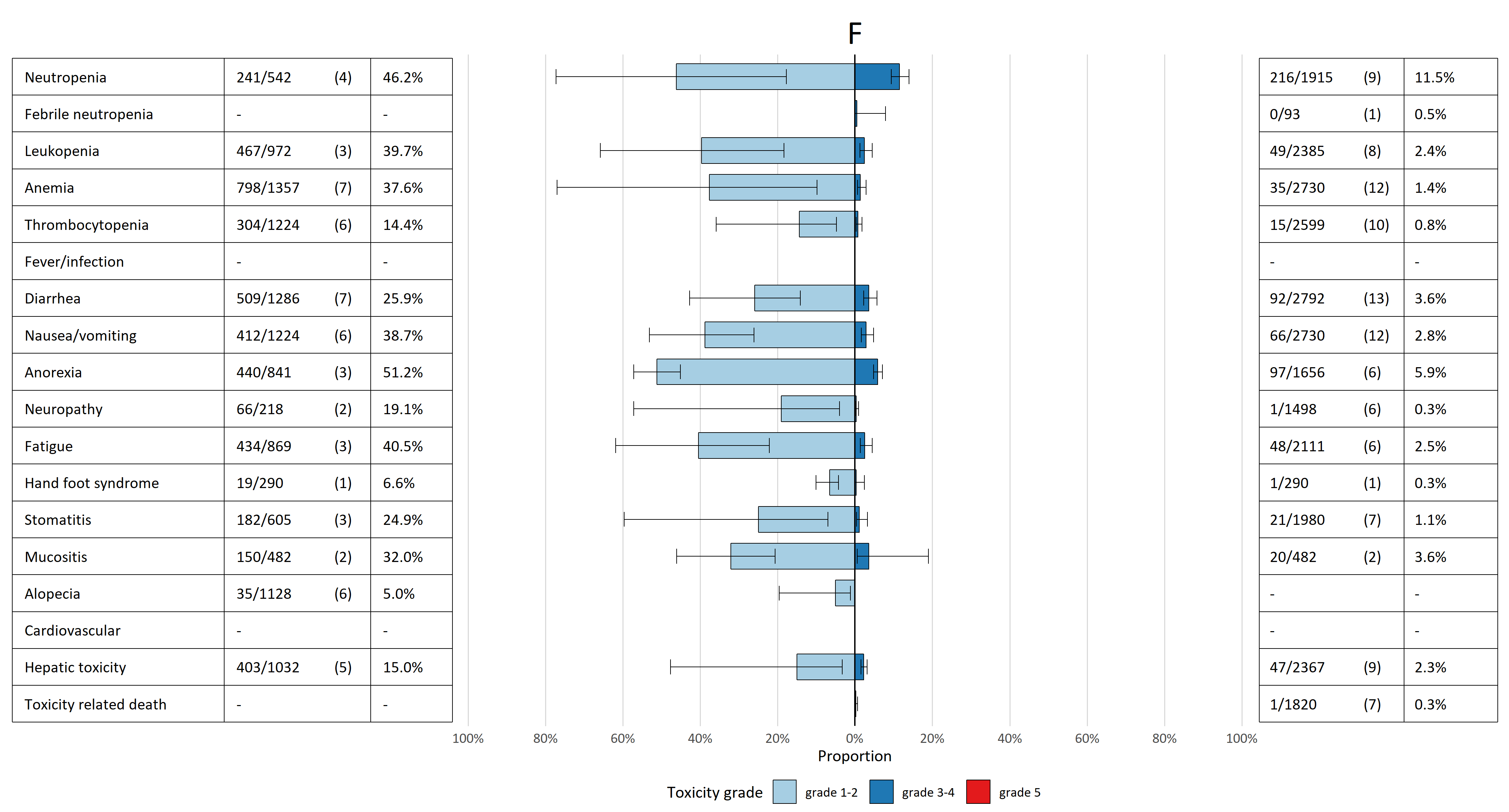


F: fluoropyrimidine


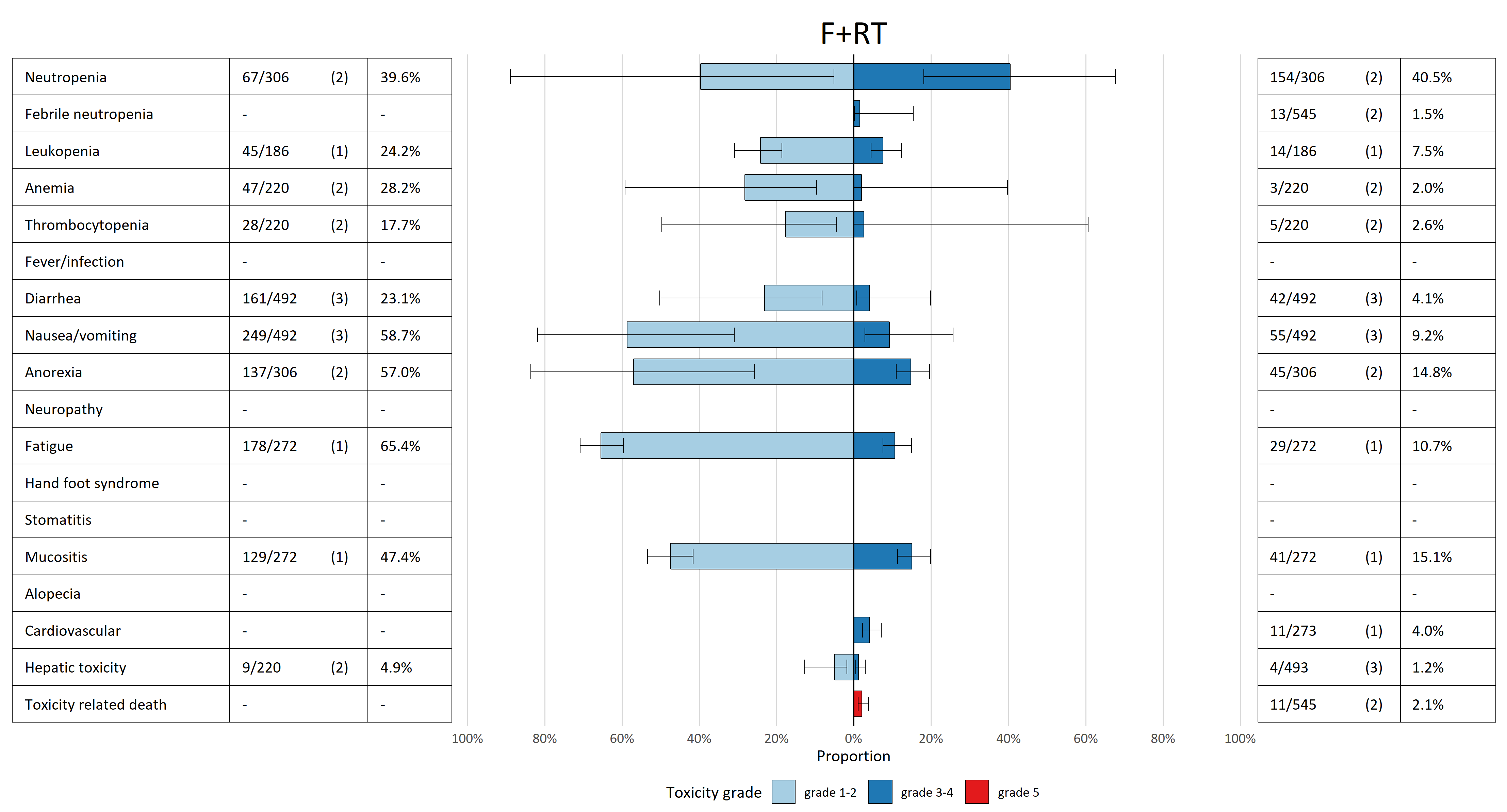


F+RT: fluoropyrimidine, radiotherapy


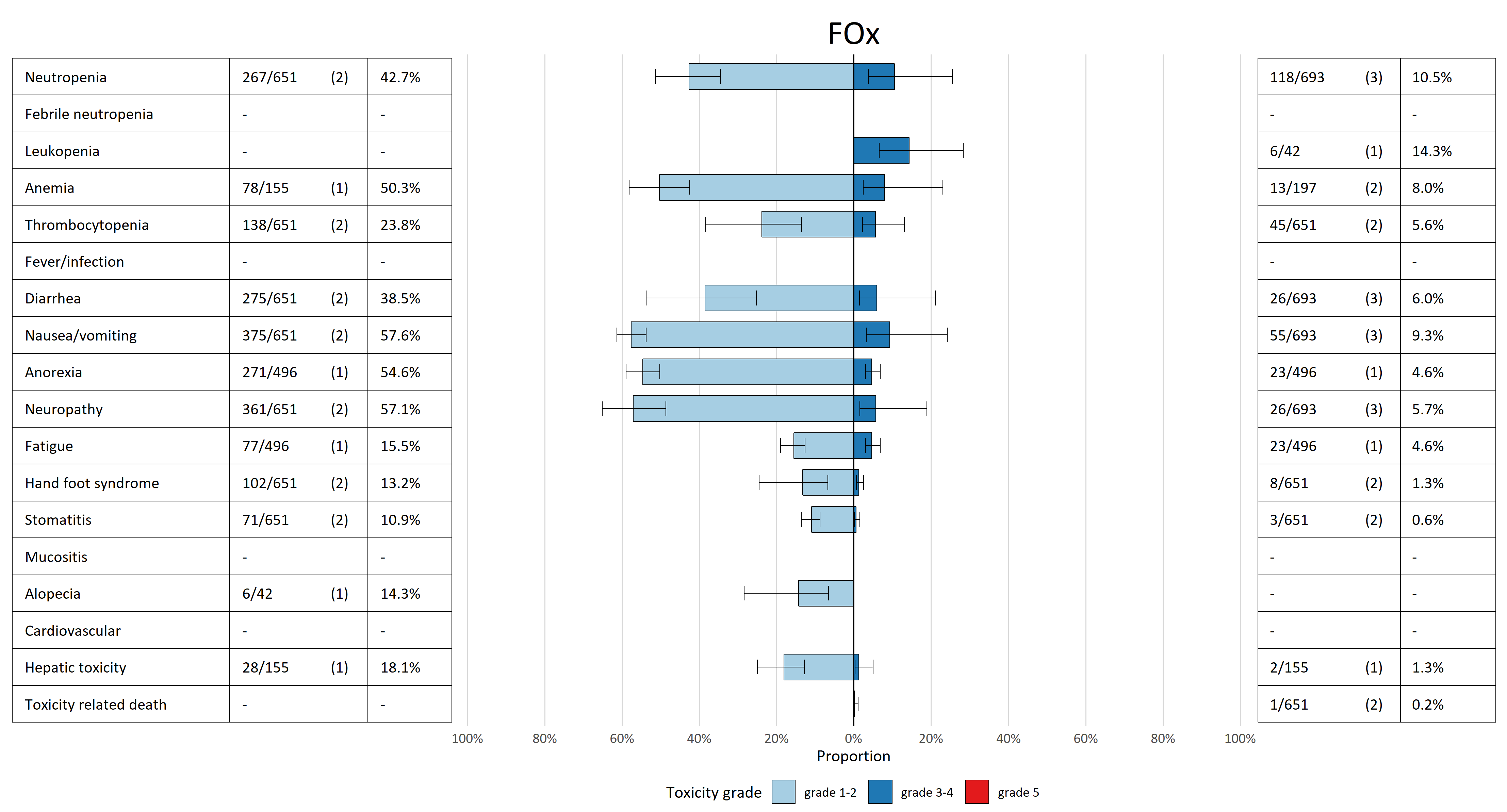


FOx: fluoropyrimidine, oxaliplatin


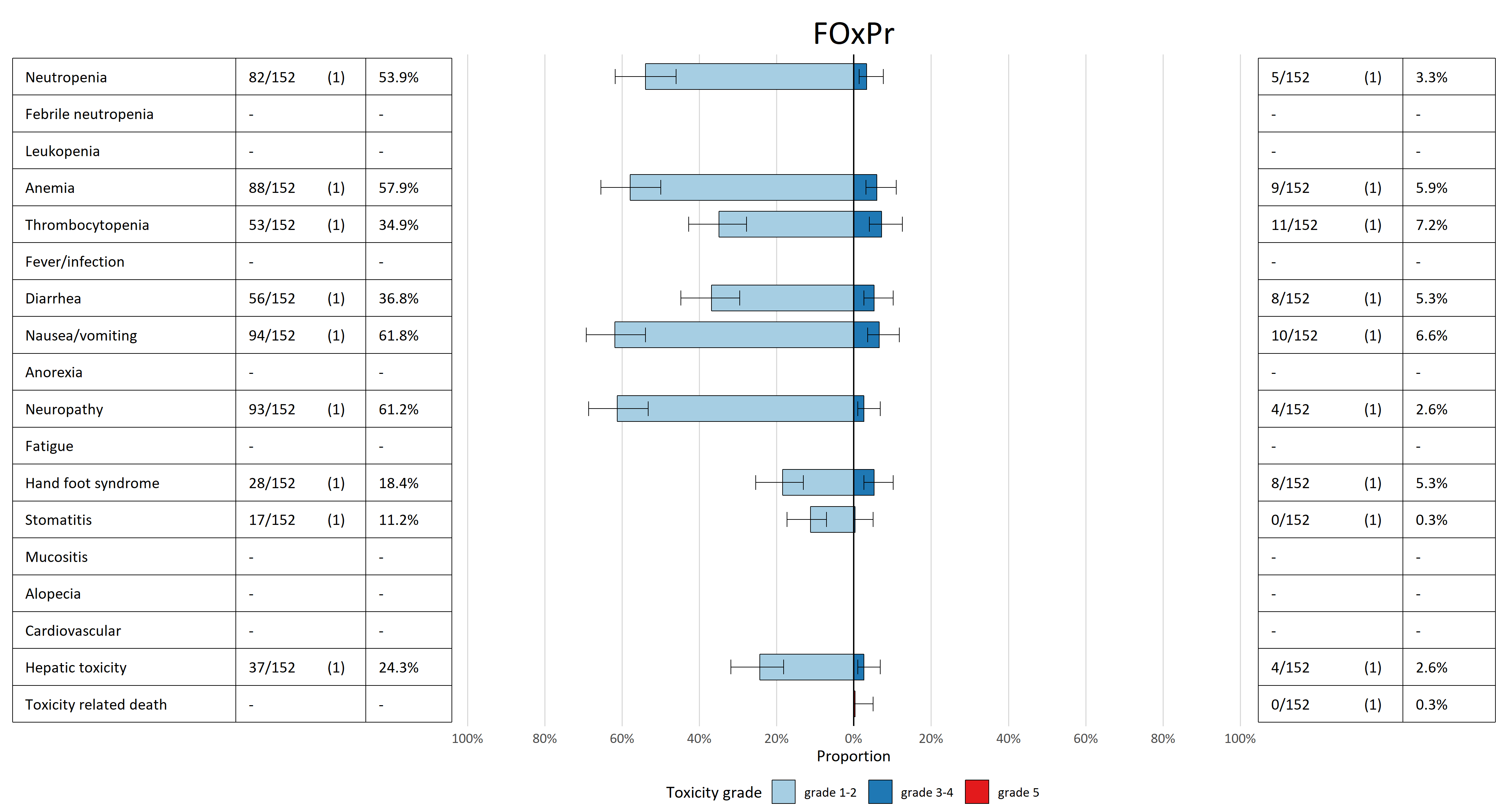


FOxpr: fluoropyrimidine, oxaliplatin, prolonged (1 year fluoropyrimidine treatment)


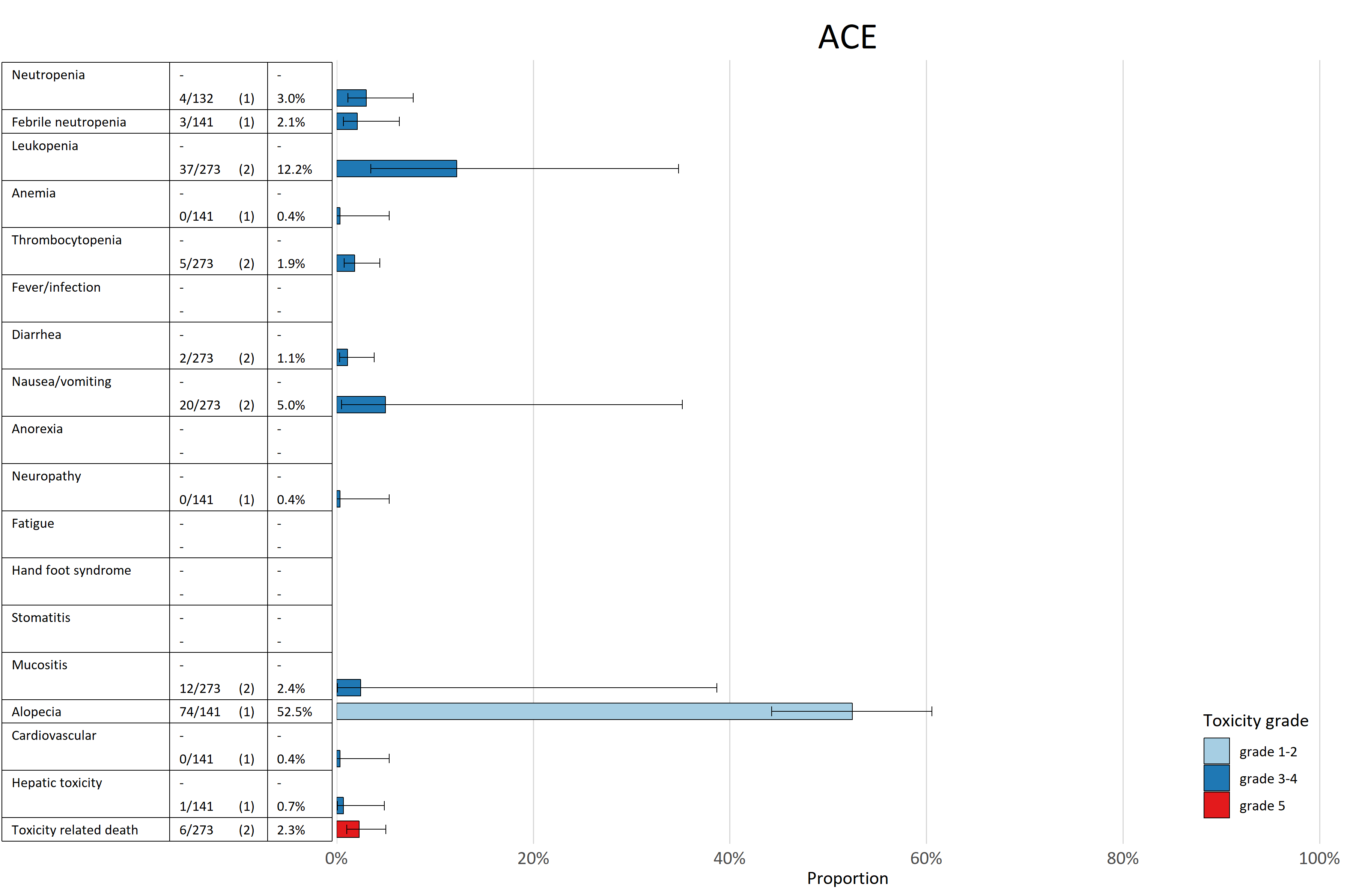


ACE: anthracycline, cisplatin, etoposide


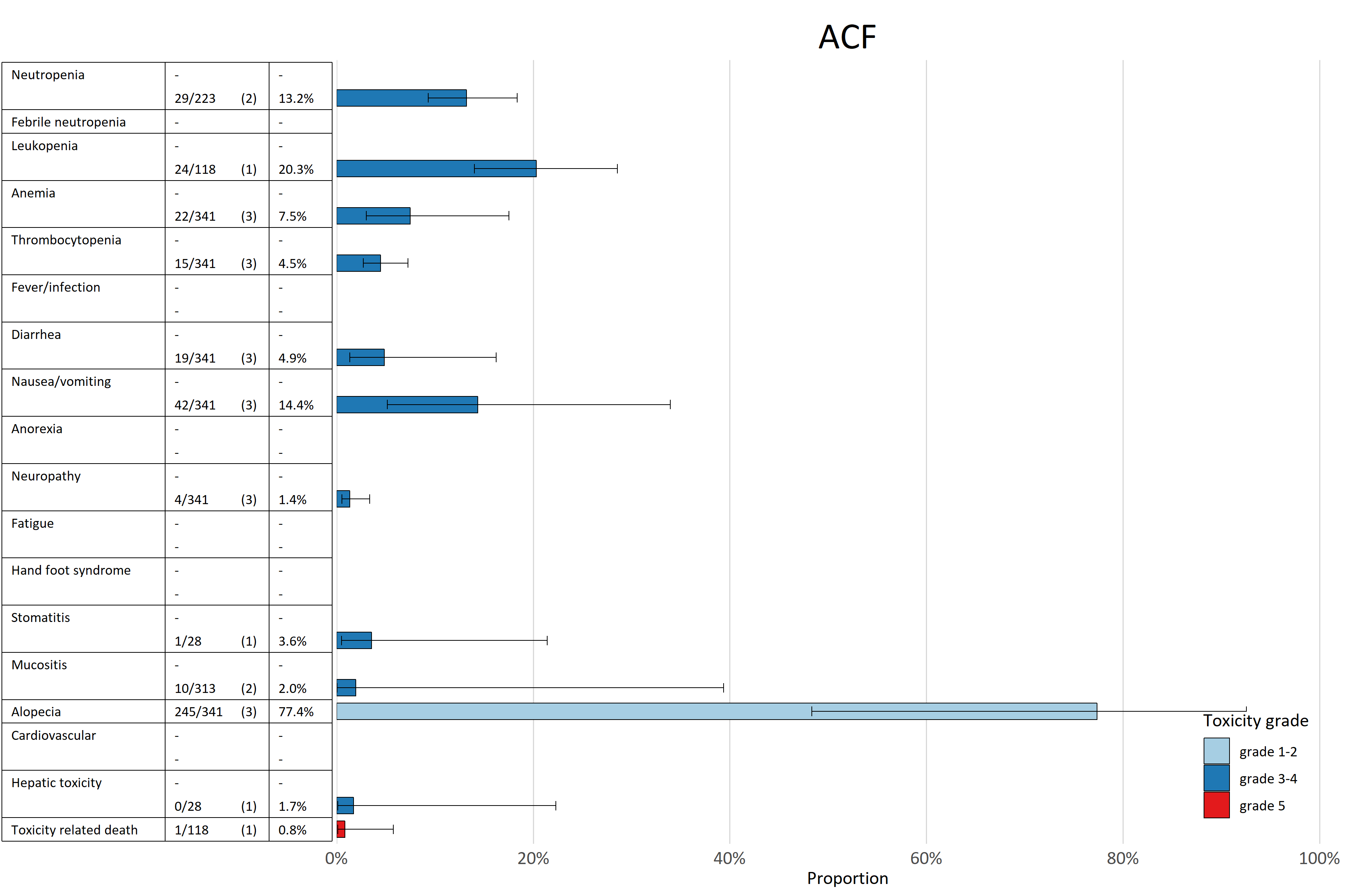


ACF: anthracycline, cisplatin, fluoropyrimidine


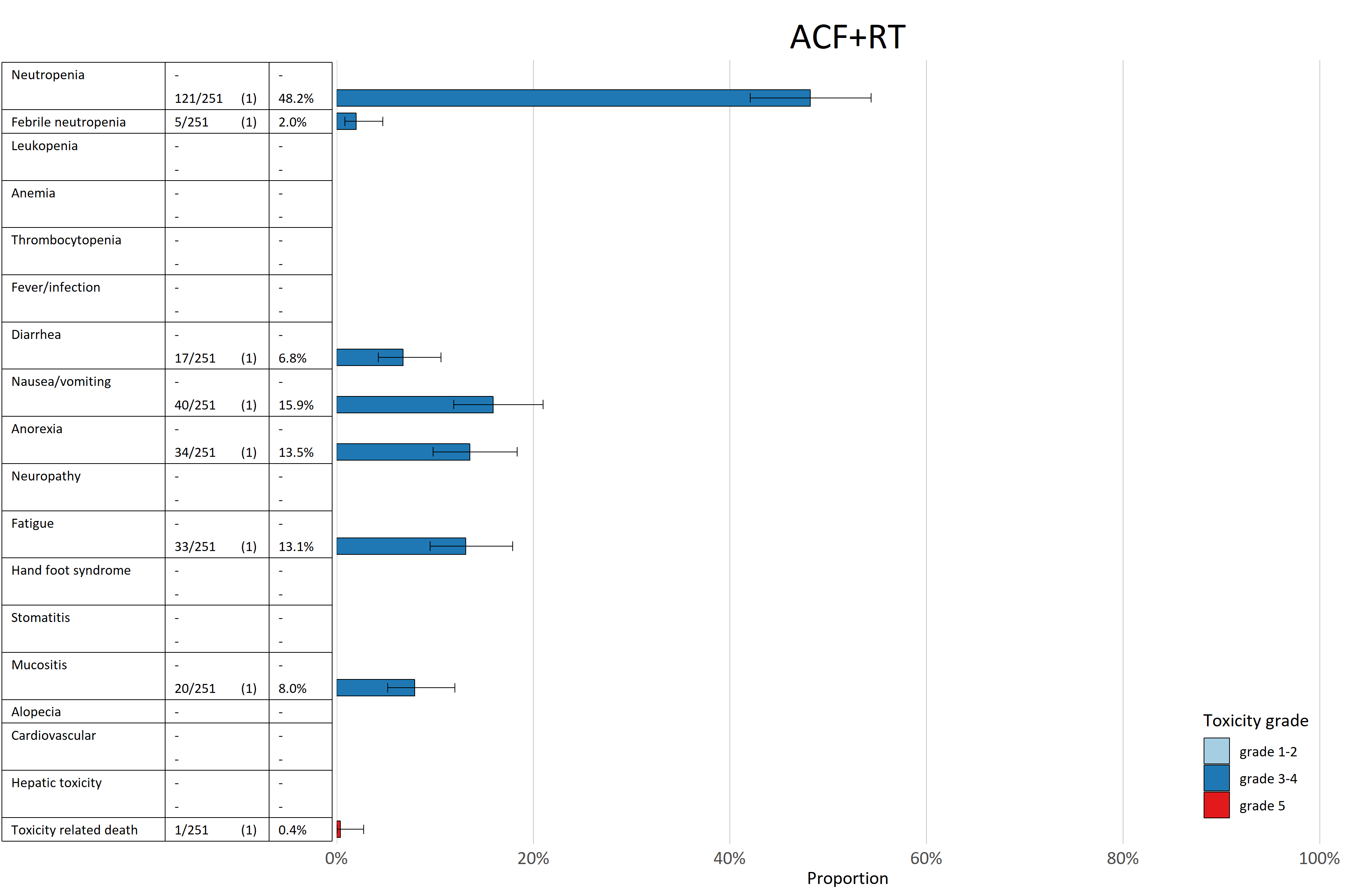


ACF+RT: anthracycline, cisplatin, fluoropyrimidine, radiotherapy


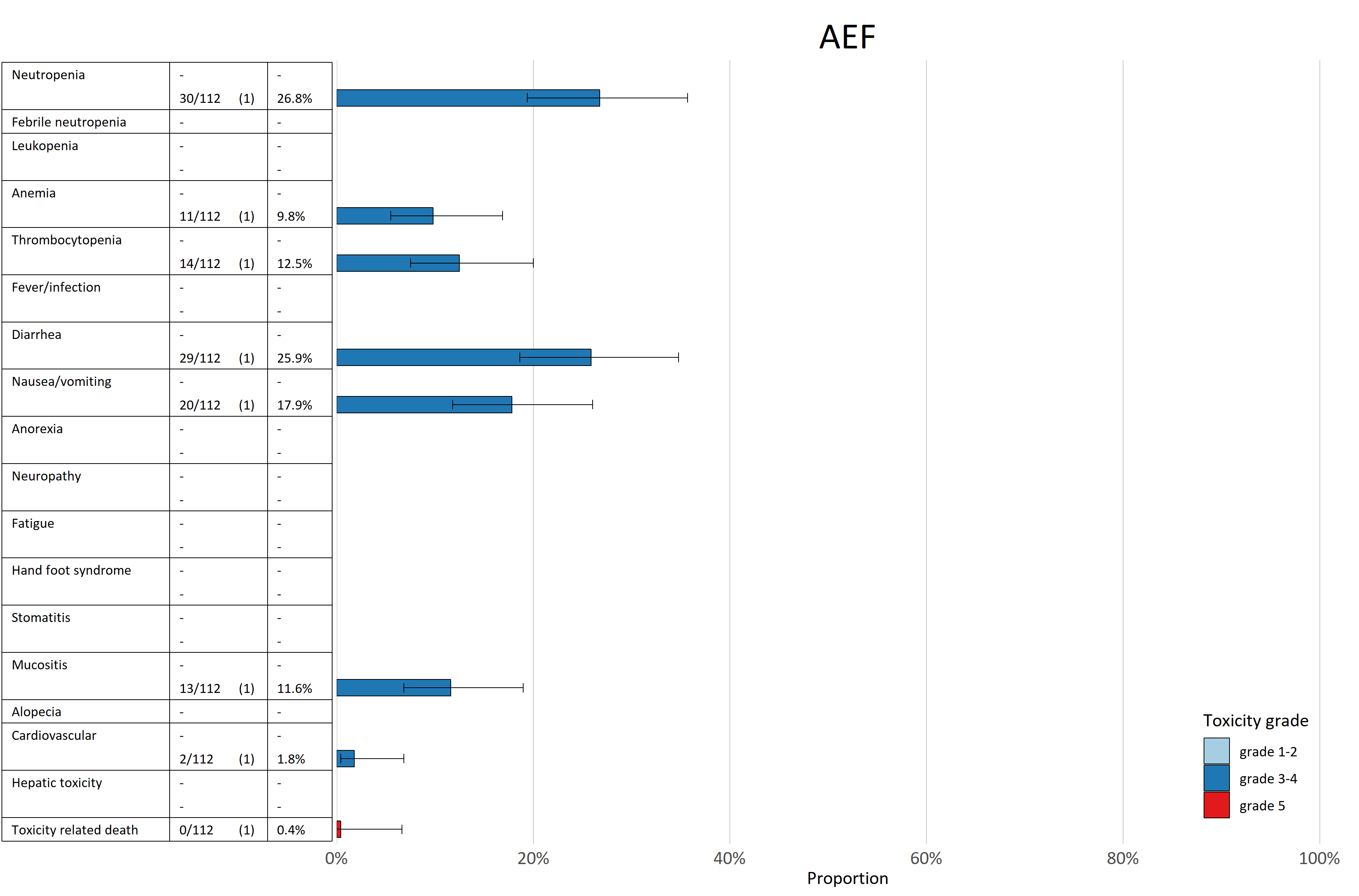


AEF: anthracycline, etoposide, fluoropyrimidine


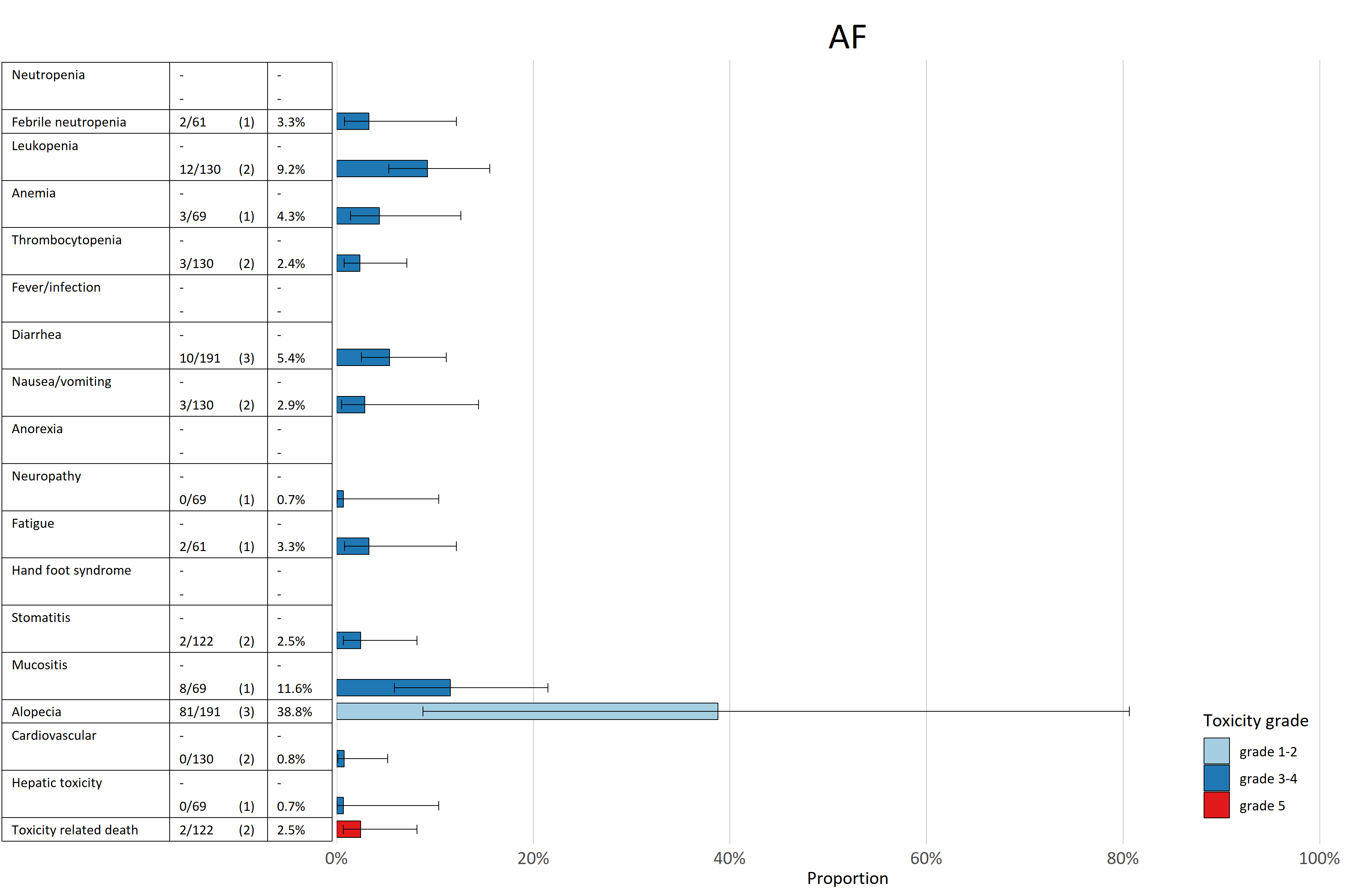


AF: anthracycline, fluoropyrimidine


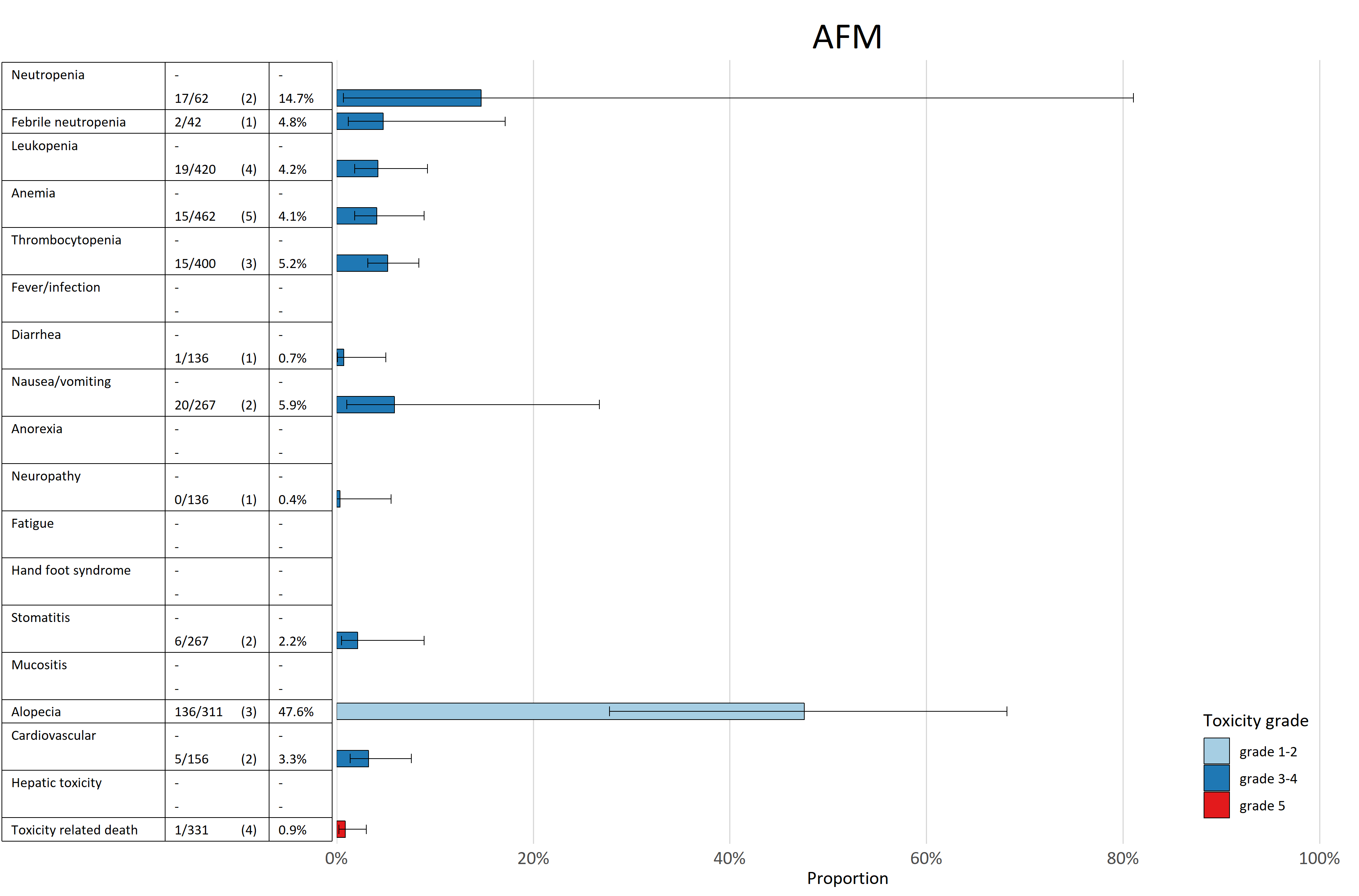


AFM: anthracycline, fluoropyrimidine, mitomycin C


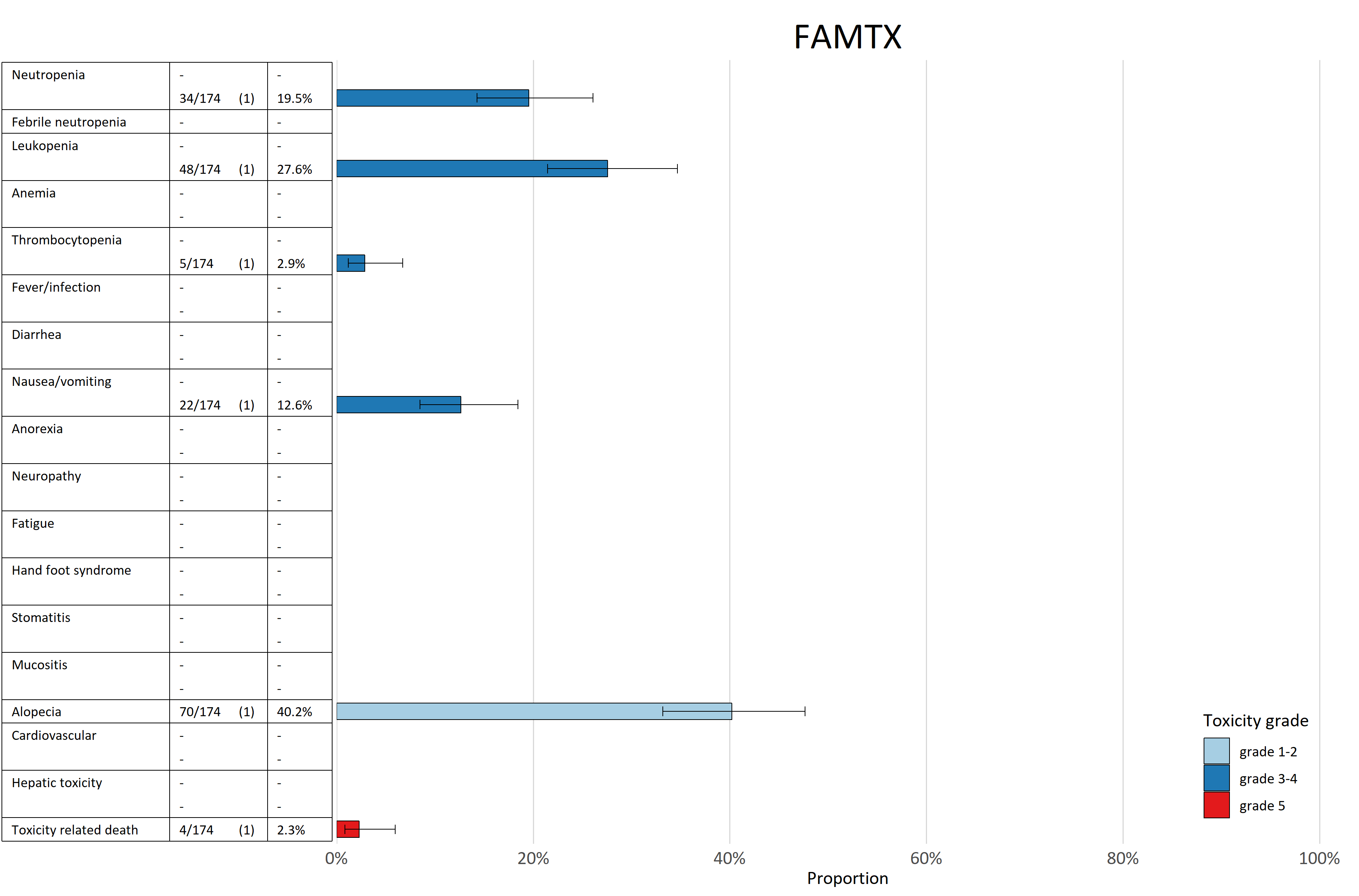


FAMTX: fluoropyrimidine, anthracycline, methotrexate


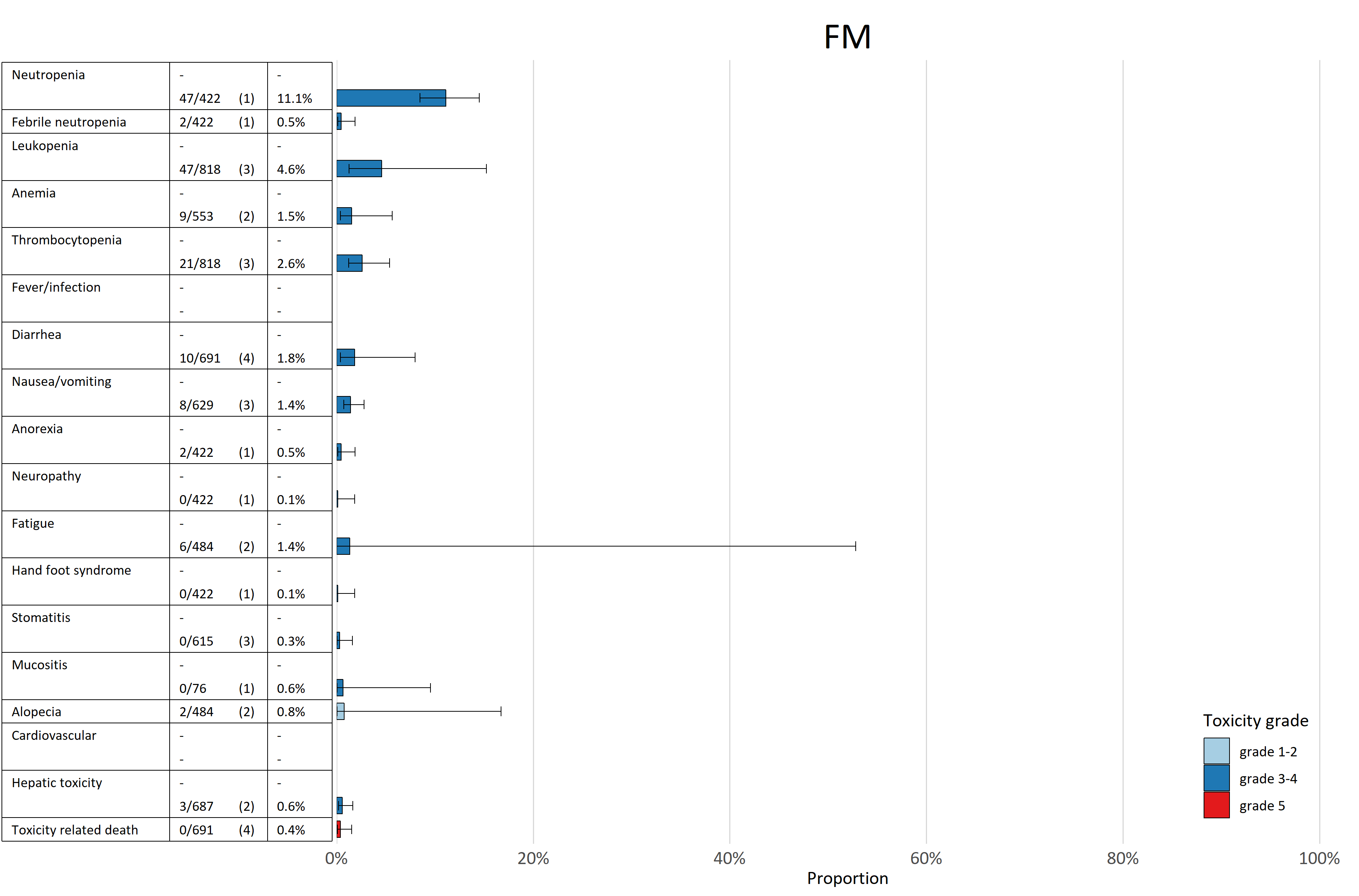


FM: fluoropyrimidine, mitomycin C


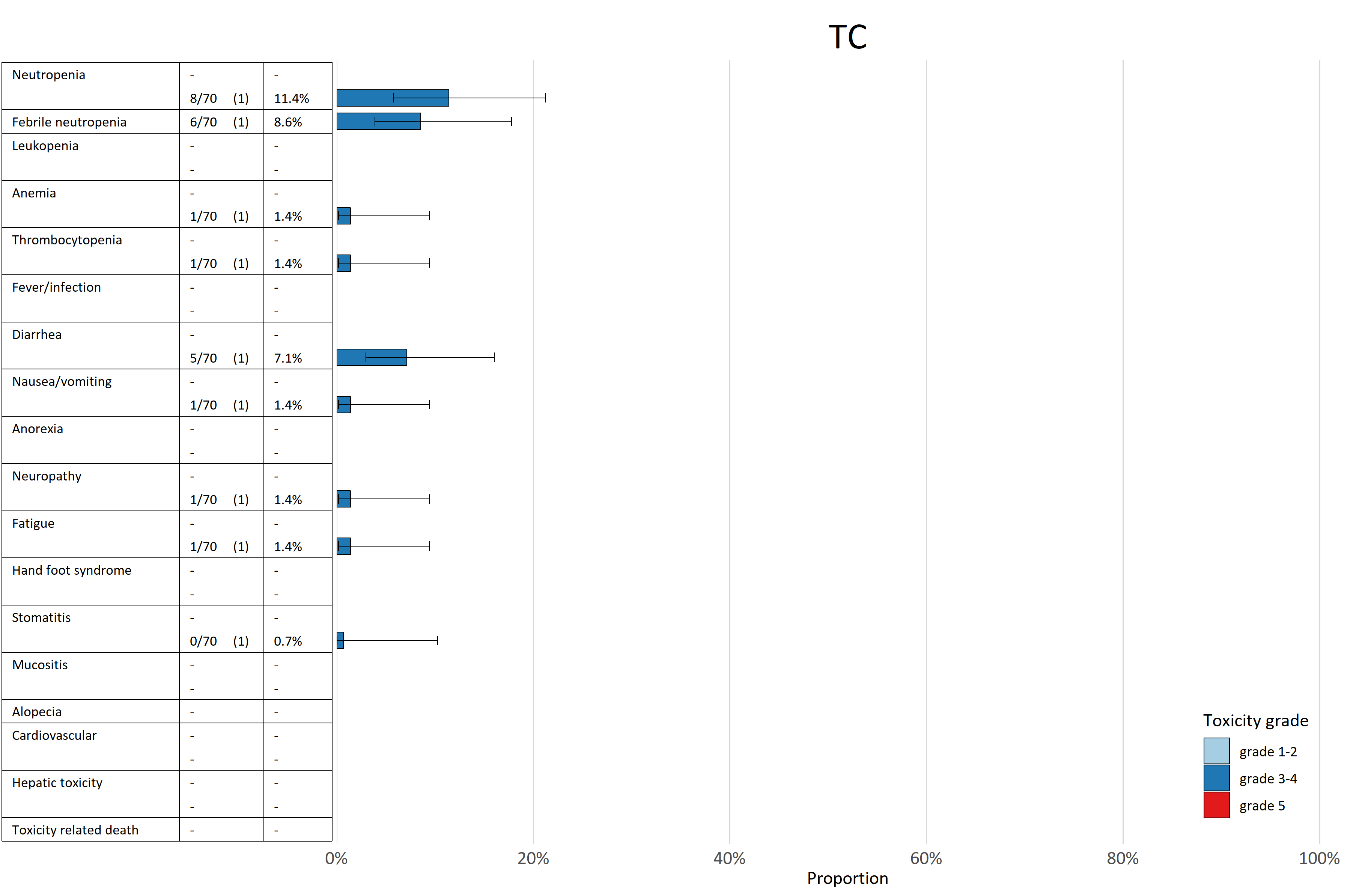


TC: taxane, cisplatin


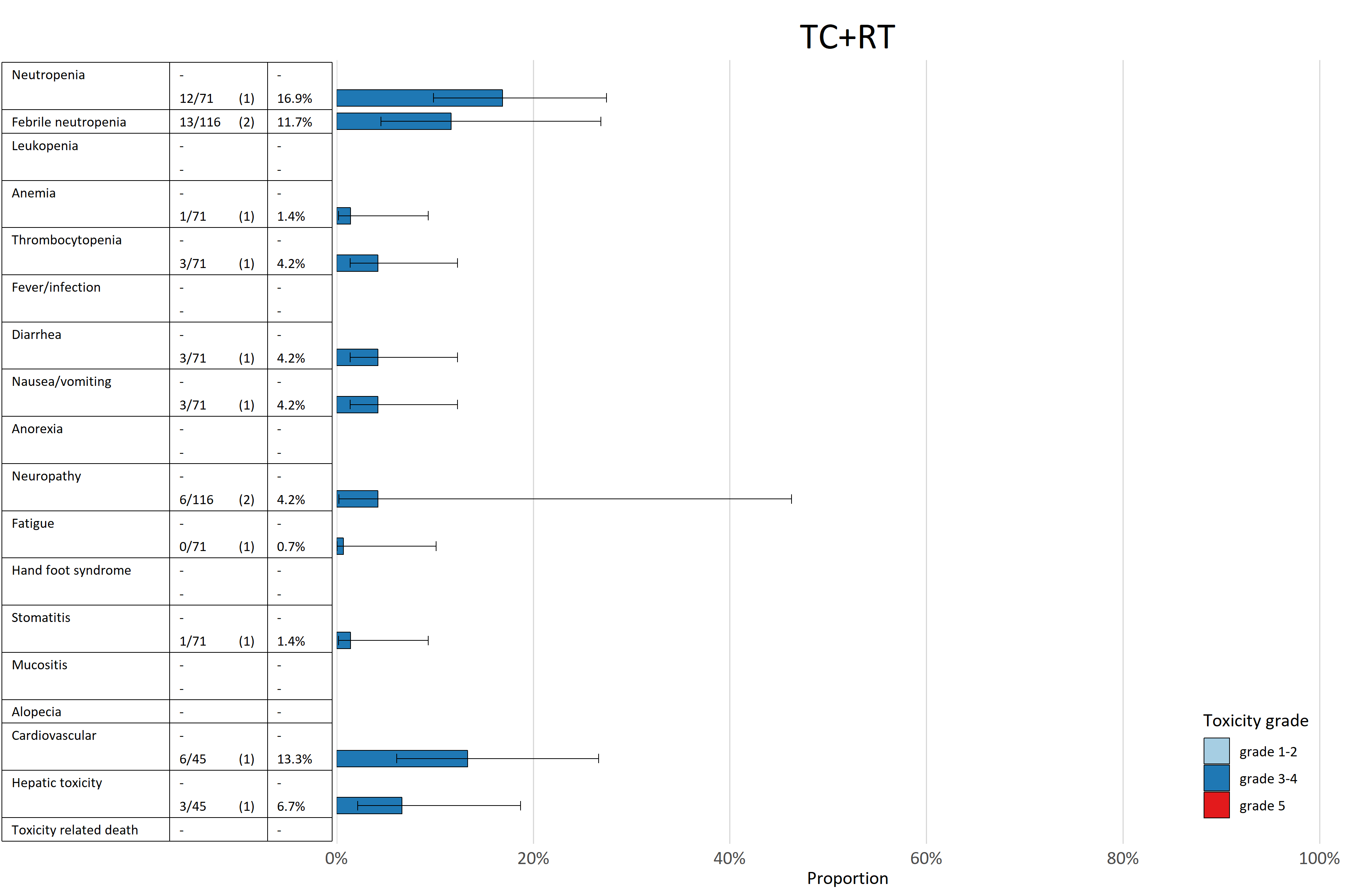


TC+RT: taxane, cisplatin, radiotherapy


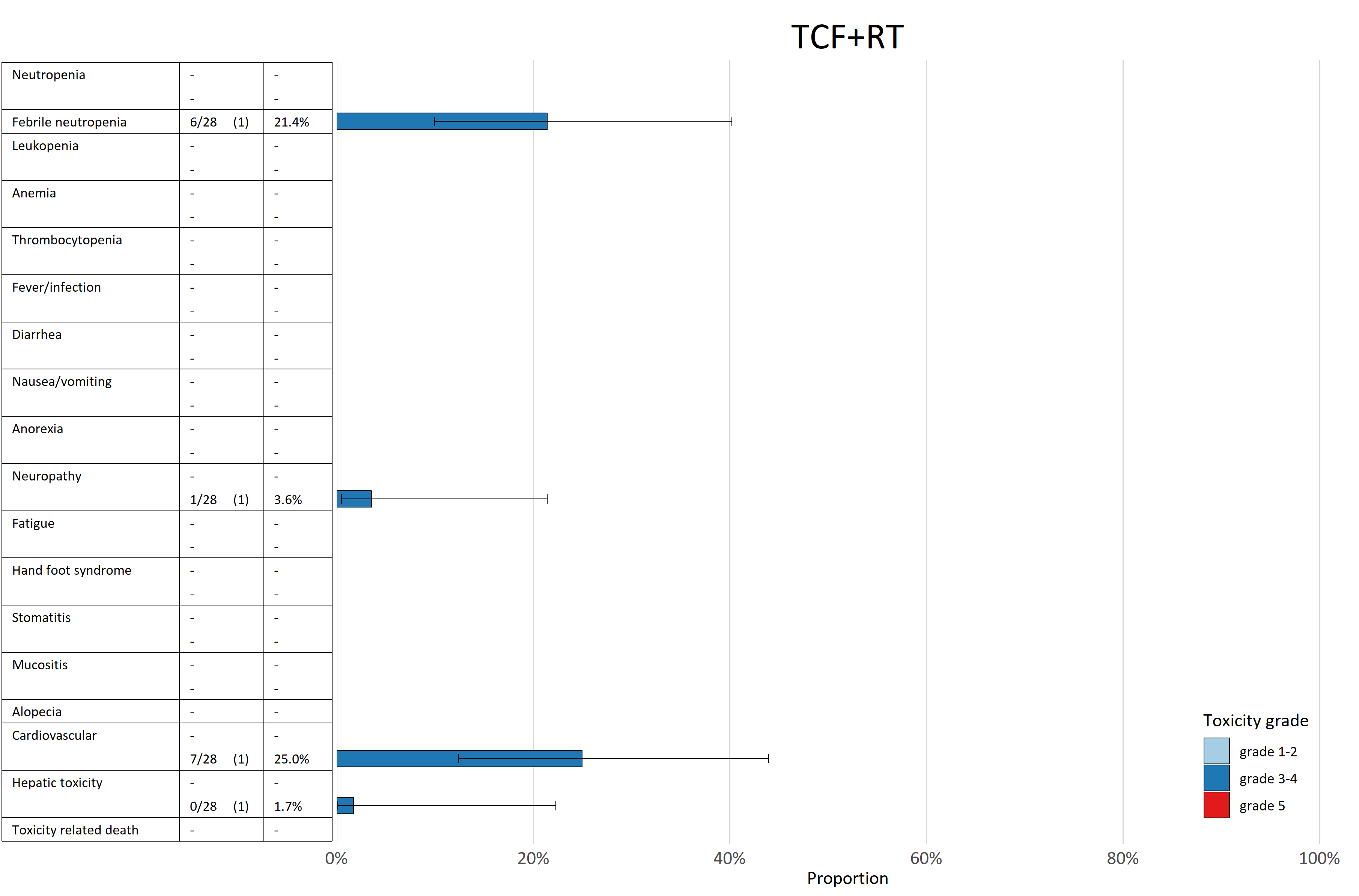


TCF+RT: taxane, cisplatin, fluoropyrimidine, radiotherapy


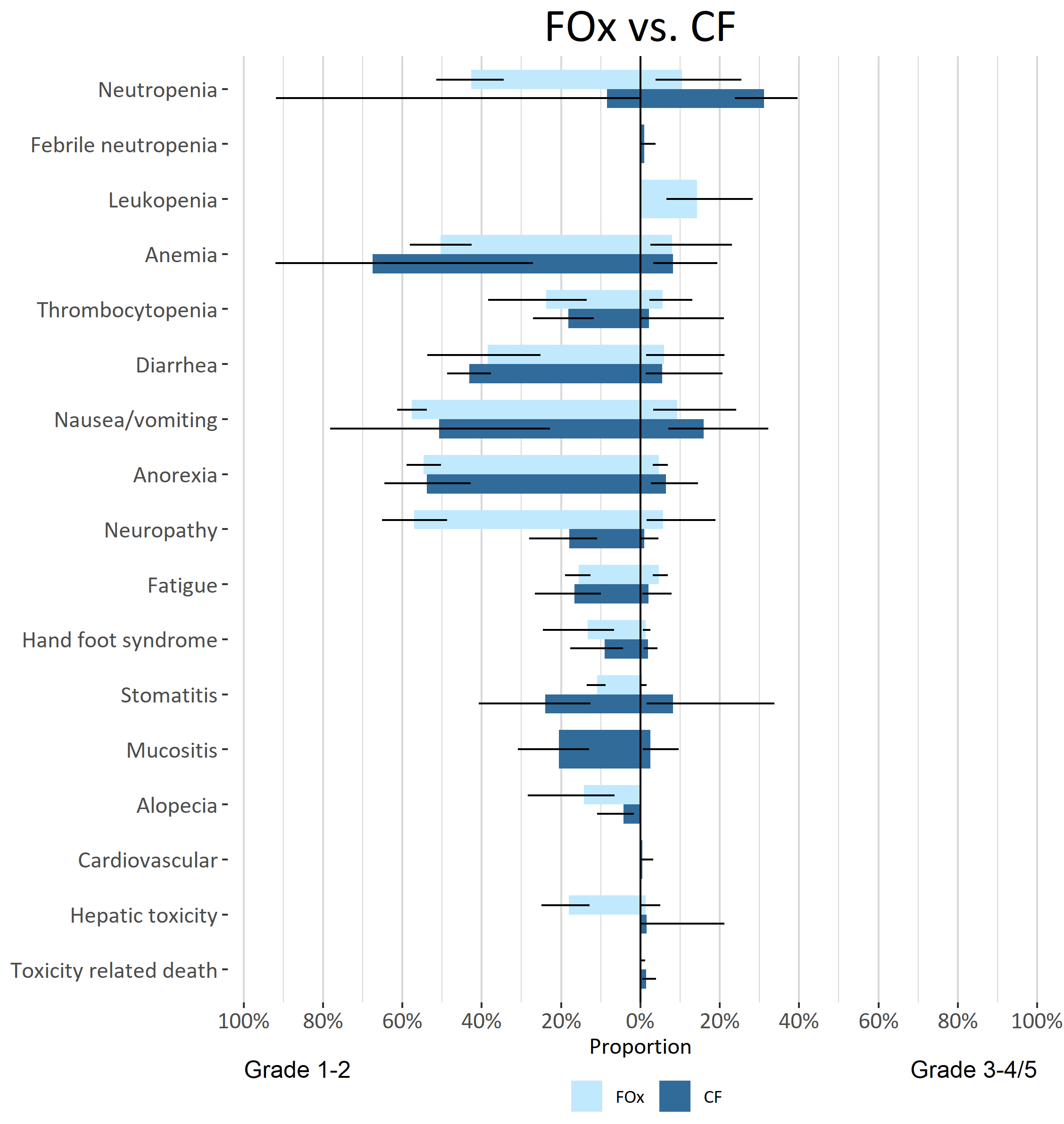


FOx: fluoropyrimidine, oxaliplatin; CF: cisplatin, fluoropyrimidine


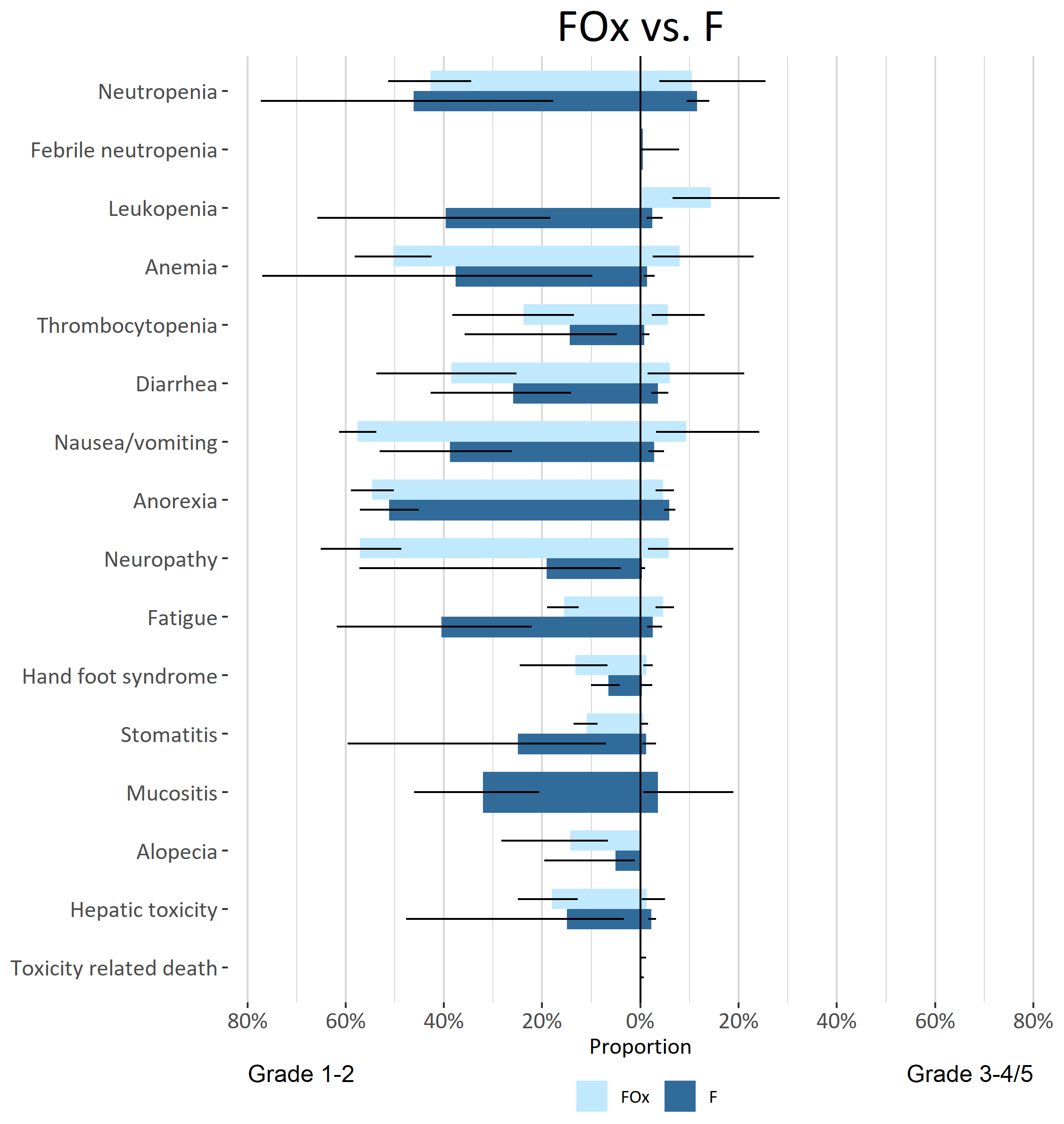


FOx: fluoropyrimidine, oxaliplatin; F: fluoropyrimidine


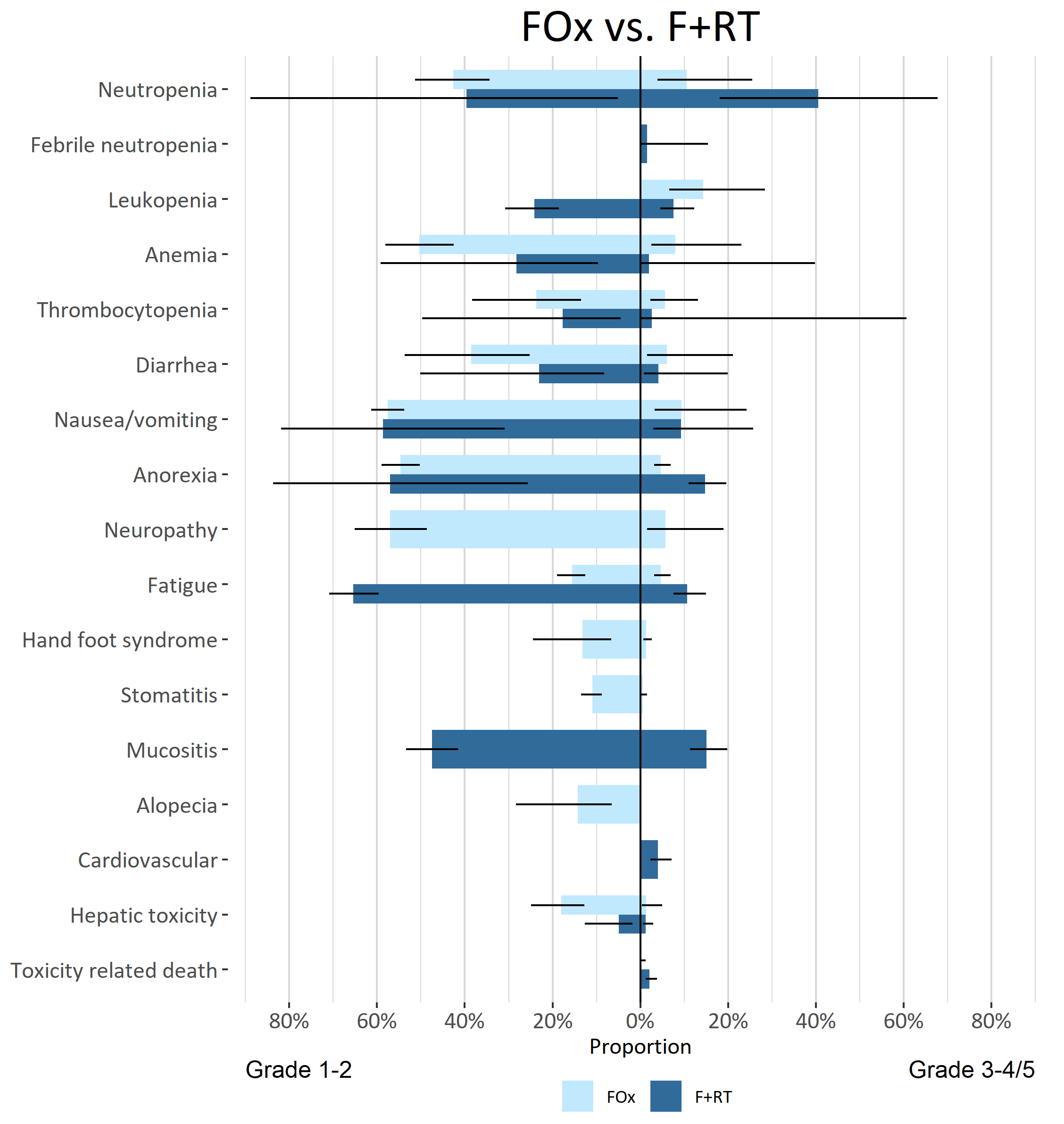


FOx: fluoropyrimidine, oxaliplatin; F+RT: fluoropyrimidine, radiotherapy


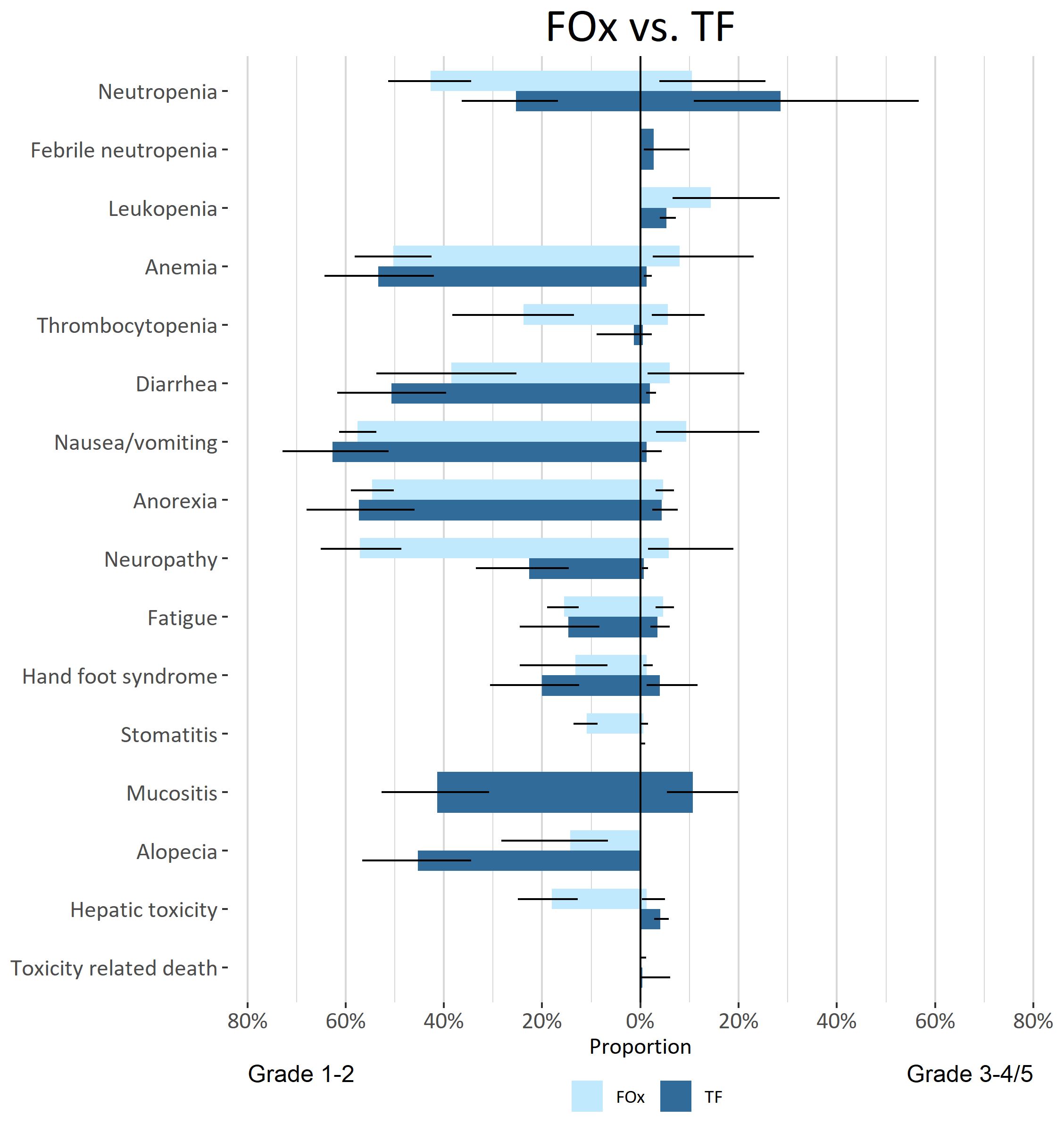


FOx: fluoropyrimidine, oxaliplatin; TF: taxane, fluoropyrimidine


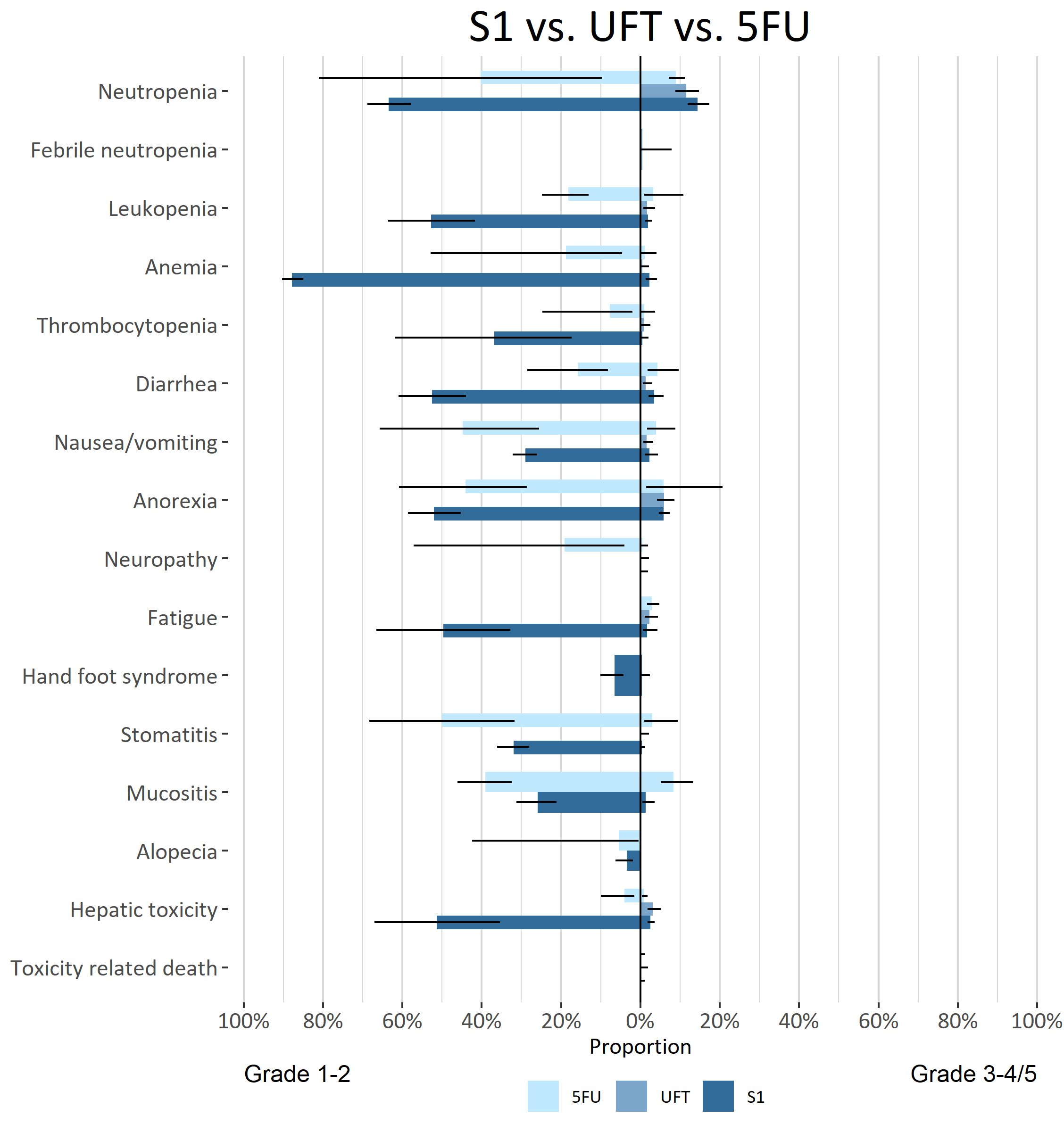


S-1: tegafur/gimeracil/oteracil; UFT: tegafur/uracil; 5-FU: fluoropyrimidine
